# Design, Synthesis and Biological Evaluation of Nitrate Derivatives of Sauropunol A and B as Potent Vasodilatory Agents

**DOI:** 10.3390/molecules24030583

**Published:** 2019-02-06

**Authors:** Lu Lu, Xuemin Rao, Rigang Cong, Chenxi Zhang, Zhimei Wang, Jinyi Xu, Genzoh Tanabe, Osamu Muraoka, Xiaoming Wu, Weijia Xie

**Affiliations:** 1 State Key Laboratory of Natural Medicines and Department of Medicinal Chemistry, China Pharmaceutical University, 24 Tong Jia Xiang, Nanjing 210009, China; 18851739997@163.com (L.L.); raochzh@163.com (X.R.); zcxciscy@163.com (C.Z.); 15295772843@163.com (Z.W.); jinyixu@cpu.edu.cn (J.X.); 2 National Certified Enterprise Technology Center, Disha Pharmaceutical Group Co., Ltd., Weihai 264205, China; crg1981@126.com; 3 Faculty of Pharmacy, Kinki University, 3-4-1 Kowakae, Higashi-Osaka, Osaka 577-8502, Japan; g-tanabe@phar.kindai.ac.jp (G.T.); muraoka@phar.kindai.ac.jp (O.M.)

**Keywords:** vasodilatory agents, NO releasing capacity, sauropunol A and B, NO donor, *Sauropus rostratus*

## Abstract

A group of nitrate derivatives of naturally occurring sauropunol A and B were designed and synthesized. Nitric oxide (NO) releasing capacity and vasodilatory capacity studies were performed to explore the structure-activity relationship of resulted nitrates. Biological evaluation of these compounds revealed that most of the synthesized mononitrate derivatives demonstrated superior releasing capacity than isosorbide mononitrate (ISMN), and **2MNS-6** even demonstrated stronger NO releasing capacity than isosorbide dinitrate (ISDN). Two dinitrates, **DNS-1** and **DNS-2**, showed higher NO releasing capacity than ISDN. Evaluation of inhibitory activities to the contractions in mesenteric artery rings revealed that **2MNS-8** and **DNS-2** showed stronger vasorelaxation activities than ISDN. High level of NO and soluble guanylyl cyclase (sGC) may be essential for the potent vasodilatory effect of **DNS-2**. The vasodilatory effects of **DNS-2** may result from cellular signal transduction of NO-sGC-cGMP. **DNS-2** was found to be the most potent sauropunol-derived nitrate vasodilatory agent for further pharmaceutical investigation against cardiovascular diseases.

## 1. Introduction

Nitric oxide has long been considered as a critical cellular signaling molecule related to different chemical and biological responses within the human body [1,2,3]. Since NO is extensively involved in numerous physiological and pathological processes, the manipulation of its biosynthesis as well as the administration of NO-releasing agents has emerged to be an effective way for the treatment of various human diseases such as cardiovascular disorders [4], neurodegeneration [5,6], inflammation [7], cancer [8,9,10,11], microorganism infection [12,13] and some immune diseases [14,15]. As a common method for drug design, molecular hybridization was usually used to introduce NO-releasing part, also known as NO donor, into an existing chemical entity. The resulting hybrid compounds usually present: (i) increased bioactivities compared to the parent compounds; (ii) new biological activities which were not observed in the parent compounds; (iii) less side effects than parent compounds [16,17,18,19,20].

**Figure 1:**
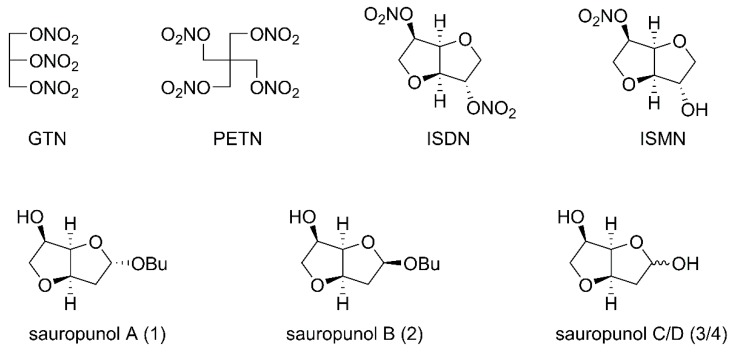
Structures of classical nitrate vasodilators and sauropunol A–D isolated from leaves of *Sauropus rostratus*.

NO donors have been used for many years in treatment of various clinical indications such as angina pectoris and coronary disease. Organic nitrate vasodilators such as gluceryl trinitrate (GTN), pentaerythritol tetranitrate (PETN), ISDN and ISMN are the oldest and the most successfully used NO donors in clinical application (Figure 1) [21]. However, one of the limitations of these drugs based on low dose nitrates is that they cannot conquer angina, and only show vein diastolic functions [22]. Thus, design and discovery of new NO-releasing compounds with stronger activity seems to be necessary.

*Sauropus rostratus* is the only reported plant with promising therapeutic value in the genus *Sauropus*. It was used in traditional Chinese medicine to treat cough, constipation and bronchitis [23]. Previous reports revealed that aqueous and alcohol extracts of *Sauropus rostratus* exhibited potent biological activities, including antibacterial, anti-inflammatory, analgesic and free radical-scavenging effects [24,25,26]. However, the detailed pharmaceutical investigation such as structure identification, organic synthesis and biological evaluation of single constituents of *Sauropus rostratus* was limited until a group of 2-deoxy-3,6-anhydro hexofuranoside derivatives **1**–**4** (Figure 1) were identified and isolated from leaves of *Sauropus rostratus* in 2014 [27]. Anhydro sugars constitute a specific and distinctive category of carbohydrates with intriguing physical, chemical and biological properties and thus, have attracted considerable attention from different chemical and pharmaceutical researchers, including our group [28,29,30]. Based on our recently developed synthetic strategy to construct 3,6-anhydro monosaccharides [31], the four naturally occurring 2-deoxy-3,6-anhydro hexofuranoside analogs **1**–**4** were synthesized and named by us [32]. The subsequent in vivo biological evaluation revealed that one of these anhydro sugars, sauropunol B, exhibited anti-inflammatory activity which is comparable with that of indomethacin [32]. In the meantime, the backbone structure similarity between sauropunol A–D and ISDN as well as ISMN prompted us to introduce NO donors into their structures, aiming at discovering new nitric oxide-releasing compounds as potential vasodilatory agents. Thus, in this study, a group of nitrate derivatives of sauropunol A and B were designed and synthesized. The NO-releasing abilities of these compounds were then tested in vitro and the vasorelaxation activities of these compounds were evaluated using isolated rat mesenteric arterial rings to shed light on the potential pharmaceutical applications of these naturally derived compounds for cardiovascular diseases.

## 2. Results

### 2.1. Chemistry

**Figure 2:**
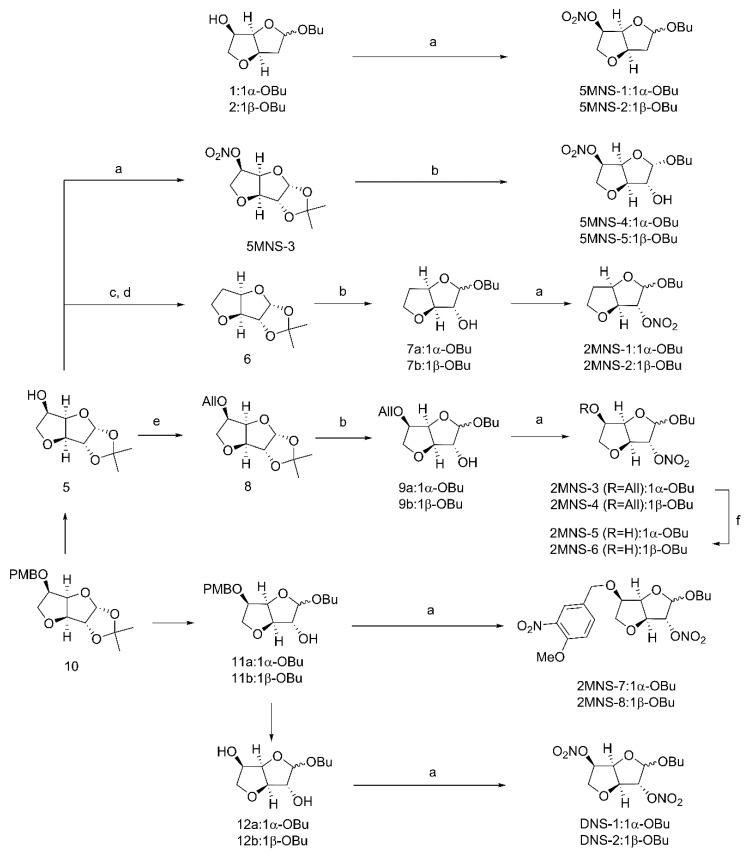
Preparation of nitrate derivatives. Reagents and conditions: (**a**) HNO_3_, Ac_2_O, 0 °C; (**b**) *p*-TSA, *n*-BuOH, rt; (**c**) PTC-Cl, DMAP, MeCN, rt; (**d**) AIBN, Bu_3_SnH, toluene, 130 °C; (**e**) 3-bromopropene, NaH, DMF, 0 °C-rt; (**f**) 10% Pd/C, *p*-TSA, MeOH, 50 °C.

Natural products **1**/**2** previously synthesized by our group [32] were directly treated with fuming nitric acid to give target 5-mononitrate derivatives **5MNS-1** and **5MNS-2** (Figure 2) [33]. In the other route, secondary alcohol **5** [32] was treated with fuming nitric acid to provide **5MNS-3**. Deacetonization of **5MNS-3** and the subsequent glycosidation were conducted to give target 5MNS-4 and **5MNS-5** [34]. In the meantime, **5** was subjected to a Barton-McCombie reaction to give intermediate **6** [35,36]. In a similar manner, **6** was transferred to a pair of anomers **7a**/**7b**, which were then directly subjected to nitration using fuming nitric acid to give target 5-deoxy-2-mononitrate derivatives **2MNS-1** and **2MNS-2**.

Also starting from **5**, the 5-hydroxyl group was first protected by an allyl group. The resulting intermediate **8** was transferred to a pair of anomers **9a**/**9b**, which were then directly subjected to nitration using fuming nitric acid to give the target 2-mononitrate derivatives **2MNS-3** and **2MNS-4**. Selective removal of the allylic protection of **2MNS-3** and **2MNS-4** provided another two 2-mononitrate derivatives **2MNS-5** and **2MNS-6**. Interestingly, when we conducted nitration reactions upon the secondary alcohols **11a**/**11b** [32], a nitro group was simultaneously attached on the phenyl ring moiety to give **2MNS-7** and **2MNS-8** as another two 2-mononitrate derivatives. Finally, diols **12a**/**12b** [32] which upon nitration reaction were converted to the target 2,5-dinitrate derivatives **DNS-1** and **DNS-2**.

### 2.2. In Vitro Nitro Oxide Releasing Capacities

**Figure 3:**
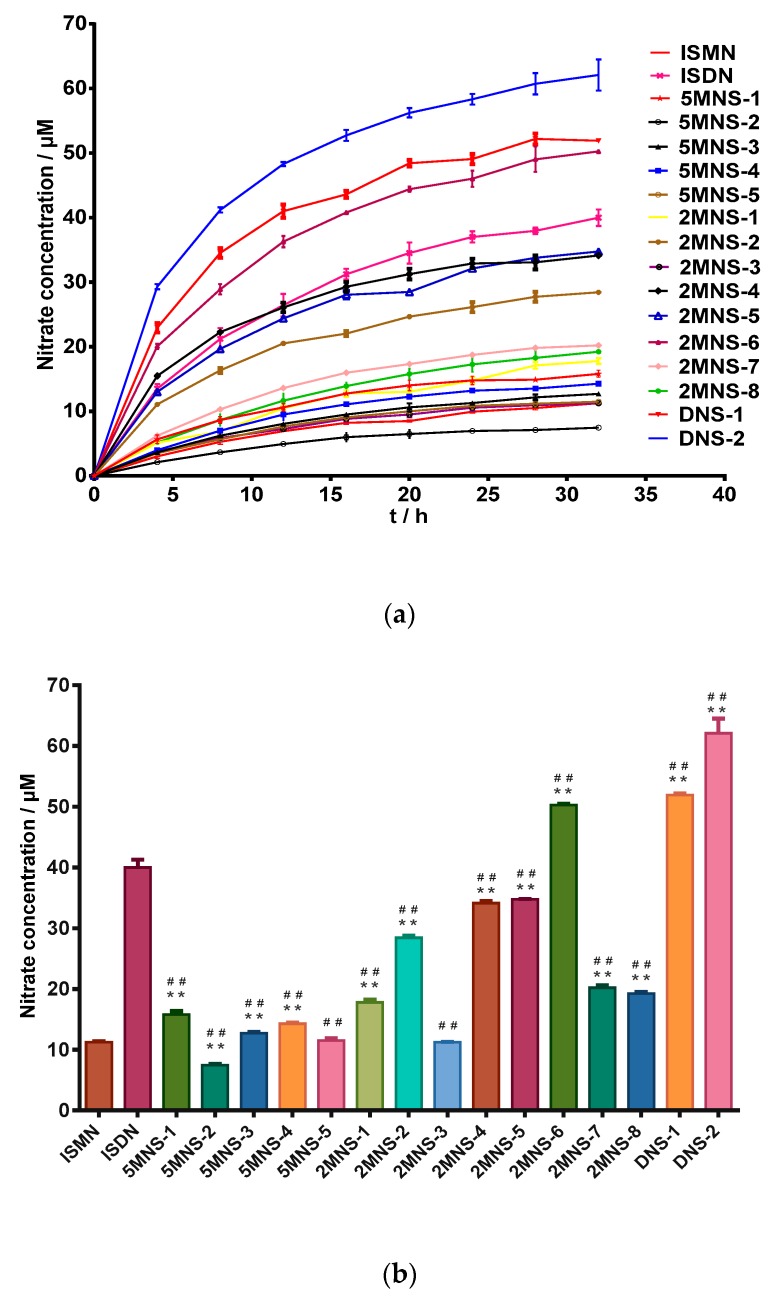
(**a**) level of nitrite for the test compounds (100 μM) over duration of 32 h by Griess assay. (**b**) Nitrate concentration for the test compounds (100 μM) at 32 h by Griess assay. Data are expressed as the mean ± SD (*n* = 6). * *p* < 0.05, ** *p* < 0.01 vs. ISMN, ^#^ *p* < 0.05, ^##^ *p* < 0.01 vs. ISDN.

In vitro NO releasing capacities of all synthesized NO donors were first measured by a Griess reaction [37,38,39]. As expected, all synthesized sauropunol-type nitrate derivatives showed NO releasing capacities in a time-dependent manner, as shown in Figure 3.

In this evaluation, ISMN and ISDN were used as positive controls. To our delight, the two synthesized 2,5-dinitrate derivatives **DNS-1** and **DNS-2** showed higher NO releasing capacities than ISDN and most of synthesized mononitrate derivatives demonstrated superior NO releasing capacities than ISMN. The 2-mononitrate derivative **2MNS-6** even demonstrated superior NO releasing capacity than ISDN. The above evidence indicated that the presence of a carbohydrate structure (in the case of sauropunol-type nitrates) may further enhance the NO-releasing potency of the resulted nitrates when compared with ISMN and ISDN bearing similar bicyclic isosorbide skeletons. It was not surprising to find that 2,5-dinitrate derivatives **DNS-1** and **DNS-2** exhibited higher NO releasing quantities than all the other mononitrate derivatives (*p* < 0.05).

### 2.3. Vasodilatory Effects on Isolated Rat Mesenteric Arterial Rings

Vasodilation is the main mechanism of anti-angina agents. The reduction in blood pressure caused by a NO donor vasodilator leads to a decrease in myocardial oxygen consumption. In addition, the dilation of coronary arteries and reduction of cardiac preload lead to an increase of myocardial oxygen supply. Moreover, release of NO can protect ischemic cardiomyocytes and inhibit the formation of thrombus. The subtle blood pressure variation of peripheral resistance vessels can result in great blood pressure variation of mesenteric artery [40,41]. Thus, isolated mesenteric arterial rings were used to examine the vasodilatory effects of sauropunol-type nitrate derivatives. Contractions were induced by phenylephrine or KCl in mesenteric artery rings [42,43].

After precontraction of mesenteric artery rings with phenylephrine, nitrate derivatives were added to the bath when sustained contractions were obtained. The preliminary vasdilatory effects of nitrate derivatives on the mesenteric vascular rings in vitro were obtained and relaxation rates were showed in Table 1. The synthesized 2,5-dinitrate **DNS-2** showed stronger vasorelaxation activities than ISDN. Some mononitrate derivatives such as **5MNS-1**, **5MNS-2**, **5MNS-3**, **5MNS-4**, **2MNS-3**, **2MNS-4**, **2MNS-7** and **2MNS-8** showed stronger vasorelaxation activities than ISMN. Even mononitrates such as **5MNS-1**, **5MNS-3**, **2MNS-4**, **2MNS-8** showed stronger vasorelaxation activities than ISDN. **5MNS-2**, **2MNS-3**, **2MNS-7** and **DNS-1** showed vasodilatory activities equivalent to that of ISDN. It can be easily observed that the synthesized 2,5-dinitrate derivative **DNS-2** with the highest NO releasing capacity consistently showed the strongest vasodilatory effect.

In the next biological evaluation, nitrate derivatives with relaxation rates of more than 30% were selected to investigate their dose-response relationships. Thus, all potent sauropunol-type nitrate derivatives showed vasodilatory capacities in a dose-dependent manner (see Supplementary Materials). As demonstrated in Table 2 left column, **DNS-2** and **2MNS-8** were the most potent vasodilatory agent with IC_50_ of 6.02 μM, and 6.94 μM. 2-mononitrates **2MNS-3** showed stronger vasodilatory potency than ISDN with IC_50_ of 12.08 μM. 2-mononitrates **2MNS-4** showed almost the same vasodilatory potency compared to ISDN.

In the meantime, the vasodilatory capacities were also tested on the contractions induced by KCl in mesenteric artery rings. In a similar procedure, the preliminary vasdilatory effects of nitrate derivatives on the mesenteric vascular rings in vitro were obtained and the relaxation rates were showed in Table 1. Two synthesized 2,5-dinitrate derivatives **DNS**-**1** and **DNS-2** showed stronger potential vasorelaxation activities than ISDN. Most mononitrate derivatives such as **5MNS-1**, **5MNS-2**, **5MNS-3**, **5MNS-4**, **2MNS-4**, **2MNS-6**, **2MNS-7** and **2MNS-8** showed higher vasorelaxation activities than ISMN. Even mononitrates such as **2MNS**-**6**, **2MNS**-**7** and **2MNS**-**8** showed more potent vasorelaxation activities than ISDN. It could be easily observed that **2MNS**-**7**, **2MNS**-**8**, **DNS**-**1** and **DNS**-**2** showed relatively higher vasodilatory effects.

Similarly, nitrate derivatives with relaxation rate more than 30% were selected to investigate their dose-response relationship. Thus, five potent sauropunol-type nitrate derivatives also showed vasodilatory capacities in a dose-dependent manner (see Supplementary Materials). IC_50_ values were showed in Table 2 right column. In this examination, **2MNS**-**7** and **2MNS**-**8** exhibited the most potent vasodilatory activities with IC_50_ of 5.94 μM and 5.52 μM. **DNS**-**1** and **DNS**-**2** displayed high vasodilatory potency with IC_50_ of 11.14 μM and 10.03 μM which were comparable with ISDN.

### 2.4. Effects of ODQ and PITO on Vasodilatory Effects of DNS-2

In order to investigate whether **DNS-2** display vasodilatory effect through cellular signal transduction of NO-sGC-cGMP, 2-phenyl-4,4,5,5-tetramethylimidazoline-1-oxyl 3-oxide (PTIO) and 1H-[1,2,4]oxadiazolo[4,3-a]quinoxalin-1-one (ODQ) were utilized in this experiment. PTIO is a stable radical scavenger for nitric oxide. It has significant inhibitory activity against NO biological actions without affecting NO synthase [44,45,46]. ODQ is a highly selective, irreversible, heme-site inhibitor of sGC [47,48].

**Figure 4:**
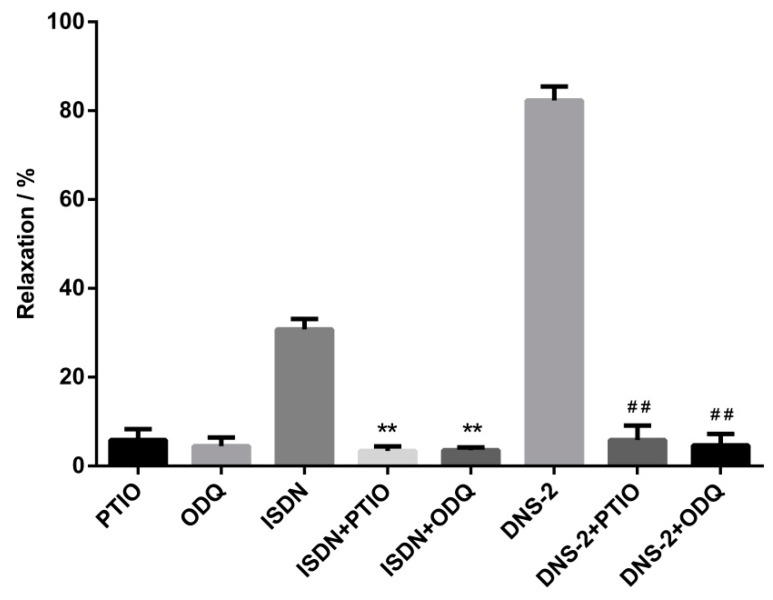
Inhibitory effects of DNS-2 (30 μM) in the presence of PTIO (100 μM) or ODQ (10 μM) or PTIO (100 μM) + ODQ (10 μM) on the contractions induced by phenylephrine (1 μM) in mesenteric artery rings. Data are expressed as the mean ± SD (*n* = 3). ** *p* < 0.01 vs. ISDN, ^##^ *p* < 0.01 vs. DNS-2.

After pre-contraction of mesenteric artery rings with phenylephrine, the contracted rings were pretreated with or without PTIO, ODQ for 15 min, and then treated with or without **DNS**-**2**. The vasdilatory effect of **DNS**-**2** on the mesenteric vascular rings in vitro was obtained and relaxation rates were showed in Figure 4. In this biological evaluation, the relaxation rates of **DNS**-**2** was reduced from 82.30 ± 3.16% to 5.86 ± 3.25% when NO scavenger PTIO was presented. The relaxation rate of **DNS**-**2** was reduced to 4.67 ± 2.57% when specific sGC inhibitor ODQ was presented.

## 3. Discussion

In an in vitro nitro oxide releasing assay, some 2-mononitrate derivatives are more likely to release NO than 5-mononitrate derivatives that bearing similar skeletons as **2MNS**-**1**, **2MNS**-**2**, **2MNS**-**5** and **2MNS**-**6** showed stronger NO releasing capacities than **5MNS**-**1**, **5MNS**-**2**, **5MNS**-**4** and **5MNS**-**5** (*p* < 0.05). **DNS**-**2** displayed stronger NO releasing capacity than **DNS**-**1**, indicating that the β-anomer rather than the α-anomer of 2,5-dinitrate derivatives can more easily release NO (*p* < 0.05). The same phenomenon was also observed for 2-mononitrate derivatives (*p* < 0.05). On the other hand, the reverse situation was encountered in the case of 5-mononitrate derivatives, as the α-anomers **5MNS**-**1** and **5MNS**-**4** showed greater NO releasing abilities than their β-counterparts **5MNS**-**2** and **5MNS**-**5**, respectively (*p* < 0.05). The performance of **5MNS**-**4**, **5MNS**-**5**, **2MNS**-**5** and **2MNS**-**6** indicating that 2-mononitrates other than 5-mononitrates more easily release NO (*p* < 0.05).

In the study of inhibitory effects on the contractions induced by phenylephrine in mesenteric artery rings, anomeric stereochemistry also played an important role in vasodilatory activities of each compounds. As for 2,5-dinitrates, the β-anomer **DNS**-**2** showed greater vasodilatory effect than its α-counterpart **DNS**-**1** (*p* < 0.05). Similarly, the β-anomers of 2-mononitrates showed greater or almost equal vasodilatory effects than their α-counterparts. In the case of 5-mononitrates, **5MNS**-**1** and **5MNS**-**4** displayed stronger vasodilatory effects than **5MNS**-**2** and **5MNS**-**5** indicating that α-anomers rather than β-anomers of 5-mononitrates tend to possess stronger vasodilatory effects (*p* < 0.05). The above findings concerning the effects of anomeric stereochemistry on bioactivities were consistent with those obtained in previous NO releasing experiments. In addition, there is an interesting phenomenon in case of 2-mononitrate derivatives that compounds **2MNS**-**3**, **2MNS**-**4**, **2MNS**-**7** and **2MNS**-**8** possessing allyl groups or *p*-methoxybenzyl groups showed stronger vasodilatory effects than compounds **2MNS**-**1**, **2MNS**-**2**, **2MNS**-**5** and **2MNS**-**6** (*p* < 0.05) indicating that sterically hindered substitution at 5-C position may play a positive role to increase vasodilatory effects.

In the study of inhibitory effects on the contractions induced by KCl in mesenteric artery rings, similar structure-activity relationship of 2-mononitrate and 5-mononitrate derivatives could be concluded for compared with those in former studies. In addition, there is an interesting phenomenon in the case of 2-mononitrate derivatives that **2MNS**-**7** and **2MNS**-**8** possessing *p*-methoxybenzyl groups showed unexpectedly potent vasodilatory effects in this test, comparable with those of **DNS**-**1** and **DNS**-**2** (*p* > 0.05).

In the study to verify the contributions of NO and sGC on the vasodilatoy effect of **DNS**-**2**, the results showed that treatment with PTIO or ODQ did not affect contracted mesenteric artery rings whereas the introduction of **DNS**-**2** alone remarkably vasodilated pre-contracted mesenteric artery rings. In sharp contrast, pretreatment with PTIO or ODQ significantly disminished the vasodilatory effect of **DNS**-**2**. The results indicated that the high level of NO and sGC may be essential for the potent vasodilatory effect of **DNS**-**2**.

In addition, compounds **2MNS**-**7** and **2MNS**-**8** which exhibited unexpected high vasodilatory effects produced relatively lower levels of NO. It is known that in vivo NO production from organic nitrates involves both enzymatic and nonenzymatic mechanisms. In particular, for organic nitrate esters (such as ISDN, ISMN, and GTN) mechanisms involving cytochrome P450 enzymes and glutathione S-transferase has been postulated [41]. Thus, further mechanistic studies rather than preliminary nitric oxide releasing assays on compounds **2MNS**-**7** and **2MNS**-**8** will be necessary in our next investigation. Among all nitrate derivatives, **DNS**-**2** showed higher NO releasing capacity and stronger vasodilatory effects against contractions induced by both phenylephrine and KCl in mesenteric artery rings, indication **DNS**-**2** was the most ideal sauropunol-derived nitrate against cardiovascular diseases.

## 4. Materials and Methods

### 4.1. General Information

Flash column chromatography purifications (medium pressure liquid chromatography) were carried out using silica gel 60 (200–400 mesh, Huanghai, Yantai, PR China). NMR spectra were collected on an AV-300 spectrometer (300 MHz ^1^H, 75 MHz ^13^C, Bruker Corp., Karlsruhe, Germany) using CDCl_3_ as solvent. ^1^H-NMR chemical shifts are recorded in parts per million (ppm, δ) relative to tetramethylsilane (δ 0.00 ppm) with the solvent resonance as an internal standard (CDCl_3_ δ 7.26 ppm). ^13^C-NMR chemical shifts are reported in ppm with the solvent peak (CDCl_3_ δ 77.0 ppm) as the internal standard. Coupling constants (*J*) are given in Hz. Low- and High Resolution Mass measurements was performed on a QTOF 6520 mass spectrometer (Agilent Technologies China, Beijing, China) with electron spray ionization (ESI) as the ion source. Optical rotations were determined with a DIP-370 digital polarimeter (JASCO, Shanghai, PR China). Column chromatography was effected over Silysia silica gel BW-200 (Fuji, Japan). The IR spectra were measured on an Impact 410 Fourier transform infrared spectrometer (Nicolet, Guangzhou, PR China).

### 4.2. Chemistry

#### 4.2.1. Synthesis of *Butyl 2-deoxy-3,6-anhydro-5-O-nitro-α-D-arabinohexofuranoside* (**5MNS**-**1**)

Fuming nitric acid (0.5 mL) was added to acetic anhydride (2 mL) at 0 °C. After stirring for 10 min, the solution of **1** (200 mg, 0.99 mmol) in Ac_2_O (2 mL) was added into the acetic anhydride solution of fuming nitric acid. The mixture was stirred at 0 °C for 15 min and then poured into ice-water and extracted with EtOAc. The organic layer was washed with brine, dried with Na_2_SO_4_ and then concentrated under reduced pressure to give a crude product, which on column chromatography (hexane/EtOAc:20:1) gave **5MNS**-**1** (250 mg, 80%) as a colorless oil. [α]_D_^25^ = +92.45 (c = 0.11 in CHCl_3_); IR (neat, cm^−1^): 3440, 3132, 2961, 2874, 2361, 1643, 1613, 1454, 1400, 1282, 1137, 1119, 1075, 1004, 955, 861, 753, 619, 568, 537, 516; ^1^H-NMR (CDCl_3_) δ 5.41–5.15 (m, 2H), 5.01–4.70 (m, 2H), 3.95 (dd, *J* = 5.4, 2.7 Hz, 2H), 3.78–3.52 (m, 1H), 3.42–3.38 (m, 1H), 2.24–2.12 (m, 2H), 1.66–1.48 (m, 2H), 1.34 (q, *J* = 7.4 Hz, 2H), 0.91 (t, *J* = 7.3 Hz, 3H); ^13^C-NMR (CDCl_3_) δ 106.2, 82.2, 80.2, 79.2, 67.9, 67.2, 39.2, 31.1, 18.7, 13.3. HR-ESIMS *m*/*z* [M + H]^+^: calcd for C_10_H_18_NO_6_: 248.1056; found:248.1058.

#### 4.2.2. Synthesis of *Butyl 2-deoxy-3,6-anhydro-5-O-nitro-β-D-arabinohexofuranoside* (**5MNS**-**2**)

Fuming nitric acid (0.5 mL) was added to acetic anhydride (2 mL) at 0 °C. After stirring for 10 min, the solution of **2** (200 mg, 0.99 mmol) in Ac_2_O (2 mL) was added into the acetic anhydride solution of fuming nitric acid. The mixture was stirred at 0 °C for 15 min and then poured into ice-water and extracted with EtOAc. The organic layer was washed with brine, dried with Na_2_SO_4_ and then concentrated under reduced pressure to give a crude product, which on column chromatography (hexane/EtOAc:20:1) gave **5MNS**-**2** (250 mg, 80%) as a colorless oil. [α]_D_^25^ = +58.27 (c = 0.16 in CHCl_3_); IR (neat, cm^−1^): 3440, 3132, 2962, 1639, 1454, 1400, 1282, 1251, 1137, 1121, 1071, 1036, 999, 955, 863, 619, 537, 517; ^1^H-NMR (CDCl_3_): δ 5.29–5.10 (m, 2H), 4.90 (t, *J* = 5.5 Hz, 1H), 4.85–4.77 (m, 1H), 4.20–4.10 (m, 1H), 4.08–3.99 (m, 1H), 3.86–3.75 (m, 1H), 3.42–3.30 (m, 1H), 2.27–2.12 (m, 2H), 1.62–1.54 (m, 2H), 1.46–1.33 (m, 2H), 0.93 (t, *J* = 7.3 Hz, 3H); ^13^C-NMR (CDCl_3_): δ 105.7, 83.0, 81.2, 80.7, 68.2, 66.5, 41.0, 31.5, 19.4, 13.9. HR-ESIMS *m*/*z* [M + H]^+^: calcd for C_10_H_18_NO_6_: 248.1056; found:248.1056.

#### 4.2.3. Synthesis of *1,2-O-Isopropylidene-3,6-anhydro-5-O-nitro-α-d-glucofuranoside* (**5MNS**-**3**)

Fuming nitric acid (0.5 mL) was added to acetic anhydride (2 mL) at 0 °C. After stirring for 10 min, the solution of **5** (200 mg, 0.99 mmol) in Ac_2_O (2 mL) was added into the acetic anhydride solution of fuming nitric acid. The mixture was stirred at 0 °C for 15 min and then poured into ice-water and extracted with EtOAc. The organic layer was washed with brine, dried with Na_2_SO_4_ and then concentrated under reduced pressure to give a crude product, which on column chromatography (hexane/EtOAc: 20:1) gave **5MNS**-**3** (250 mg, 80%) as a colorless oil. [α]_D_^25^ = +54.17 (c = 0.15 in CHCl_3_); IR (neat, cm^−1^): 3003, 2944, 2891, 1641, 1410, 1385, 1375, 1283, 1233, 1204, 1163, 1125, 1096, 1069, 1008, 904, 894, 863, 844, 830, 593, 539; ^1^H-NMR (CDCl_3_) δ 5.96 (d, *J* = 3.5 Hz, 1H), 5.35–5.26 (m, 1H), 5.09 (t, *J* = 4.4 Hz, 1H), 4.64 (d, *J* = 3.5 Hz, 1H), 4.57 (d, *J* = 4.2 Hz, 1H), 4.02 (dd, *J* = 10.2, 6.3 Hz, 1H), 3.93 (dd, *J* = 10.2, 5.2 Hz, 1H), 1.50 (s, 3H), 1.35 (s, 3H). ^13^C-NMR (CDCl_3_) δ 113.2, 107.5, 85.8, 84.2, 81.1, 80.7, 68.3, 27.5, 26.7.h-ESIMS *m*/*z* [M + H]^+^: calcd for C_9_H_14_NO_7_: 248.0692; found: 248.0694.

#### 4.2.4. Synthesis of *Butyl 3,6-anhydro-5-O-nitro-α-d-glucofuranoside* (**5MNS**-**4**) and *Butyl 3,6-anhydro-5-O-nitro-β-d-glucofuranoside* (**5MNS**-**5**)

To a solution of **5MNS**-**3** (200 mg, 0.81 mmol) in *n*-BuOH (7 mL) was added *p*-TSA·H_2_O (0.6 g) at room temperature. After being stirred for 48 h at room temperature, the reaction mixture was concentrated under reduced pressure to give a colorless residue, which was then subjected to column chromatography (hexane/EtOAc:8/1→hexane/EtOAc:5/1) to give compounds **5MNS**-**4** (35 mg, 16%) and **5MNS**-**5** (110 mg, 52%) as colorless oils. **5MNS-4**: [α]_D_^25^ = +92.28 (c = 0.14 in CHCl_3_); IR (neat, cm^−1^): 3441, 3132, 2962, 2874, 1642, 1604, 1454, 1401, 1282, 1138, 1119, 1070, 1003, 955, 862, 615, 568, 535, 516; ^1^H-NMR (CDCl_3_): δ 5.29–5.18 (m, 1H), 5.18–5.11 (m, 1H), 4.93 (t, *J* = 5.4 Hz, 1H), 4.59–4.43 (m, 1H), 4.19–4.07 (m, 1H), 4.08–3.88 (m, 2H), 3.84–3.73 (m, 1H), 3.59–3.48 (m, 1H), 2.73 (d, *J* = 6.3 Hz, 1H), 1.69–1.51 (m, 2H), 1.43–1.28 (m, 2H), 0.91 (t, *J* = 7.3 Hz, 3H). ^13^C-NMR (CDCl_3_) δ 104.4, 88.0, 80.2, 78.6, 76.2, 69.3, 68.1, 31.5, 19.1, 13.8. HR-ESIMS *m*/*z* [M + H]^+^: calcd for C_10_H_18_NO_7_: 264.1005; found: 264.1015. **5MNS**-**5**: [α]_D_^25^ = +92.28 (c = 0.14 in CHCl_3_); IR (neat, cm^−1^): 3430, 3132, 2963, 1640, 1614, 1454, 1401, 1282, 1120, 1069, 1004, 953, 861, 783, 751, 617, 537, 517; ^1^H-NMR (CDCl_3_): δ 5.27–5.16 (m, 1H), 5.14–5.05 (m, 1H), 5.01 (s, 1H), 4.52 (d, *J* = 5.2 Hz, 1H), 4.21 (s, 1H), 4.12–3.98 (m, 2H), 3.88–3.75 (m, 1H), 3.44–3.31 (m, 1H), 2.83 (s, 1H), 1.63–1.50 (m, 2H), 1.45–1.30 (m, 2H), 0.92 (t, *J* = 7.3 Hz, 3H). ^13^C-NMR (CDCl_3_) δ 110.4, 88.5, 81.2, 80.4, 80.2, 68.5, 67.5, 31.3, 19.3, 13.9. HR-ESIMS *m*/*z* [M + H]^+^: calcd for C_10_H_18_NO_7_: 264.1005; found: 264.1010.

#### 4.2.5. Synthesis of *Butyl 5-deoxy-3,6-anhydro-α-d-glucofuranoside* (**7a**) and *Butyl 5-deoxy-3,6-anhydro-β-d-glucofuranoside* (**7b**)

To a solution of **6** (200 mg, 1.08 mmol) in *n*-BuOH (7 mL) was added *p*-TSA·H_2_O (0.6 g) at room temperature. After being stirred for 48 h at room temperature, the reaction mixture was concentrated under reduced pressure to give a colorless residue, which was then subjected to column chromatography (hexane/EtOAc:8/1→hexane/EtOAc:5/1) to give compounds **7a** (39 mg, 18%) and **7b** (107 mg, 49%) as colorlessoils. **7a**: [α]_D_^25^ = +117.62 (c = 0.15 in CHCl_3_); ^1^H-NMR (CDCl_3_) δ 5.06 (d, *J* = 4.6 Hz, 1H), 4.78 (t, *J* = 5.0 Hz, 1H), 4.40 (dd, *J* = 4.8, 2.4 Hz, 1H), 4.08 (s, 1H), 3.94 (td, *J* = 8.3, 2.0 Hz, 1H), 3.87–3.66 (m, 2H), 3.56–3.45 (m, 1H), 2.75 (d, *J* = 7.9 Hz, 1H), 2.09–1.97 (m, 1H), 1.94–1.80 (m, 1H), 1.66–1.55 (m, 2H), 1.47–1.32 (m, 2H), 0.93 (t, *J* = 7.3 Hz, 3H).^13^C-NMR (CDCl_3_) δ 102.3, 88.4, 80.4, 77.3, 67.7, 66.4, 32.6, 31.1, 18.8, 13.3. HR-ESIMS *m*/*z* [M + H]^+^: calcd for C_10_H_19_O_4_: 203.1205; found: 203.1205. **7b**: [α]_D_^25^ = + 21.51 (c = 0.10 in CHCl_3_); ^1^H-NMR (CDCl_3_) δ 5.00 (t, *J* = 5.2 Hz, 1H), 4.94 (s, 1H), 4.36 (d, *J* = 4.8 Hz, 1H), 4.18 (s, 1H), 4.08–3.96 (m, 1H), 3.94–3.85 (m, 1H), 3.74–3.64 (m, 1H), 3.45–3.34 (m, 1H), 2.65 (s, 1H), 2.17–2.05 (m, 1H), 2.05–1.88 (m, 1H), 1.62–1.49 (m, 2H), 1.41–1.31 (m, 2H), 0.91 (t, *J* = 7.3 Hz, 3H). ^13^C-NMR (CDCl_3_) δ 109.0, 87.5, 83.9, 79.9, 67.6, 67.4, 33.7, 31.0, 18.8, 13.4. HR-ESIMS *m*/*z* [M + H]^+^: calcd for C_10_H_19_O_4_: 203.1205; found: 203.1210.

#### 4.2.6. Synthesis of *Butyl 5-deoxy3,6-anhydro-2-O-nitro-α-d-glucofuranoside* (**2MNS**-**1**) and *Butyl 5-deoxy-3,6-anhydro-2-O-nitro-β-d-glucofuranoside* (**2MNS**-**2**)

Fuming nitric acid (0.5 mL) was added to acetic anhydride (2 mL) at 0 °C. After stirring for 10 min, the solution of **7a** (200 mg, 0.99 mmol) in Ac_2_O (2 mL) was added into the acetic anhydride solution of fuming nitric acid. The mixture was stirred at 0 °C for 15 min and then poured into ice-water and extracted with EtOAc. The organic layer was washed with brine, dried with Na_2_SO_4_ and then concentrated under reduced pressure to give a crude product, which on column chromatography (hexane/EtOAc:20:1) gave **2MNS**-**1** (198 mg, 81%) as a colorless oil. [α]_D_^25^ = +119.32 (c = 0.09 in CHCl_3_); IR (neat, cm^−1^): 3440, 3335, 3132, 2961, 2874, 1642, 1613, 1454, 1440, 1283, 1138, 1120, 1070, 1044, 1018, 954, 857, 825, 624, 537, 516, 427. ^1^H-NMR (CDCl_3_) δ 5.45–5.19 (m, 1H), 4.98–4.92 (m, 1H), 4.78 (t, *J* = 5.3 Hz, 1H), 4.74–4.67 (m, 1H), 4.01 (t, *J* = 8.2 Hz, 1H), 3.86–3.67 (m, 2H), 3.45–3.32 (m, 1H), 2.08 (dd, *J* = 13.6, 5.2 Hz, 1H), 1.99–1.83 (m, 1H), 1.60–1.47 (m, 2H), 1.38–1.27 (m, 2H), 0.90 (t, *J* = 7.3 Hz, 3H). ^13^C-NMR (CDCl_3_) δ 101.4, 86.2, 83.4, 80.1, 68.1, 67.1, 32.8, 31.4, 19.2, 13.8. HR-ESIMS *m*/*z* [M + H]^+^: calcd for C_10_H_18_NO_6_: 248.1056; found: 248.1056.

In a similar manner, **2MNS**-**2** (198 mg, 81% yield) was obtained as a colorless oil from **7b** (200 mg, 0.99 mmol). [α]_D_^25^ = +37.10 (c = 0.11 in CHCl_3_); IR (neat, cm^−1^): 3441, 3132, 2962, 1648, 1616, 1454, 1400, 1302, 1270, 1120, 1091, 1071, 1030, 953, 843, 618, 537, 516. ^1^H-NMR (CDCl_3_) δ 5.21 (s, 1H), 5.05 (s, 1H), 5.02–4.89 (m, 1H), 4.52 (d, *J* = 5.1 Hz, 1H), 4.16–4.03 (m, 1H), 3.94 (td, *J* = 8.2, 2.5 Hz, 1H), 3.77–3.66 (m, 1H), 3.49–3.37 (m, 1H), 2.16–1.95 (m, 2H), 1.63–1.51 (m, 2H), 1.42–1.31 (m, 2H), 0.92 (t, *J* = 7.3 Hz, 3H). ^13^C-NMR (CDCl_3_) δ 105.5, 89.8, 85.0, 83.9, 68.4, 68.2, 34.2, 31.4, 19.3, 13.8. HR-ESIMS *m*/*z* [M + H]^+^: calcd for C_10_H_18_NO_6_: 248.1056; found: 248.1059.

#### 4.2.7. Synthesis of *1,2-O-Isopropylidene-3,6-anhydro-5-O-allyl-α-d-glucofuranoside* (**8**)

To a mixture of sodium hydride (NaH, 60% in liquid paraffin, 128 mg, 3.2 mmol) and allyl bromide (0.28 mL, 3.2 mmol) in dry DMF (10 mL) was added a solution of **5** (303 mg, 1.5 mmol) in dry DMF at 0 °C. After stirring at 0 °C for 4 h, the reaction was quenched by the addition of ice-water. The resulted solution was extracted with EtOAc. The combined extracts were washed with brine, and condensed under reduced pressure to give a yellow oil, which was then subjected to column chromatography (hexane/EtOAc:25/1), gave compound **8** (345 mg, 95%) as a pale yellow oil. [α]_D_^25^ = +40.05 (c = 0.13 in CHCl_3_); IR (neat, cm^−1^): 3132, 2990, 2386, 2285, 1648, 1399, 1234, 1164, 1070, 1017, 930, 898, 858, 828, 548, 520; ^1^H-NMR (CDCl_3_) δ 6.15–5.78 (m, 2H), 5.43–5.17 (m, 2H), 4.96–4.78 (m, 1H), 4.58 (t, *J* = 3.8 Hz, 1H), 4.49 (t, *J* = 3.7 Hz, 1H), 4.25–4.13 (m, 1H), 4.13–3.89 (m, 3H), 3.72–3.61 (m, 1H), 1.50 (s, 3H), 1.33 (s, 3H). ^13^C-NMR (CDCl_3_) δ 134.3, 118.0, 112.3, 107.2, 85.6, 85.1, 80.7, 78.9, 71.6, 69.5, 27.3, 26.7. HR-ESIMS *m*/*z* [M + H]^+^: calcd for C_12_H_19_O_5_: 243.1154; found: 243.1150.

#### 4.2.8. Synthesis of *Butyl 3,6-anhydro-5-O-allyl-α-d-glucofuranoside* (**9a**) and *Butyl 3,6-anhydro-5-O-allyl-β-d-glucofuranoside* (**9b**)

To a solution of **8** (300 mg, 1.16 mmol) in *n*-BuOH (5 mL) was added *p*-TSA·H_2_O (0.6 g) at room temperature. After being stirred for 48 h at room temperature, the reaction mixture was concentrated under reduced pressure to give a colorless residue, which was then subjected to column chromatography (hexane/EtOAc:8/1→hexane/EtOAc:5/1) to give compounds **9a** (54 mg, 18%) and **9b** (180 mg, 60%) as colorless oils. **9a**: [α]_D_^25^ = +90.43 (c = 0.16 in CHCl_3_); IR (neat, cm^−1^): 3133, 2959, 2873, 2360, 2342, 1647, 1458, 1401, 1269, 1142, 1064, 1020, 953, 860, 669, 549; ^1^H-NMR (CDCl_3_) δ 6.03–5.85 (m, 1H), 5.31 (dd, *J* = 17.0, 1.9 Hz, 1H), 5.26–5.13(m, 2H), 4.63 (t, *J* = 4.7 Hz, 1H), 4.50–4.41 (m, 1H), 4.22–4.02 (m, 3H), 4.01–3.90 (m, 2H), 3.89–3.78 (m, 1H), 3.63–3.49 (m, 2H), 3.04 (d, *J* = 7.4 Hz, 1H), 1.74–1.53 (m, 2H), 1.38 (q, *J* = 7.5 Hz, 2H), 0.93 (t, *J* = 7.3 Hz, 3H). ^13^C-NMR (CDCl_3_) δ 134.1, 117.6, 103.1, 88.0, 77.9, 77.7, 77.2, 71.4, 68.1, 67.9, 31.2, 18.9, 13.5. HR-ESIMS *m*/*z* [M + H]^+^: calcd for C_13_H_23_O_5_: 259.1467; found: 259.1465. **9b**: [α]_D_^25^ = +8.19 (c = 0.12 in CHCl_3_); IR (neat, cm^−1^): 3449, 3130, 2960, 2936, 2872, 2382, 1648, 1459, 1400, 1315, 1268, 1116, 1085, 1018, 942, 888, 843, 787, 651, 595, 553, 450; ^1^H-NMR (CDCl_3_) δ 6.03–5.85 (m, 1H), 5.44–5.26 (m, 1H), 5.25–5.16 (m, 1H), 5.02 (s, 1H), 4.83 (t, *J* = 4.7 Hz, 1H), 4.43 (d, *J* = 4.7 Hz, 1H), 4.30–4.11 (m, 2H), 4.08–3.90 (m, 3H), 3.90–3.76 (m, 2H), 3.67 (d, *J* = 3.7 Hz, 1H), 3.44–3.31 (m, 1H), 1.64–1.51 (m, 2H), 1.41–1.30 (m, 2H), 0.91 (t, *J* = 7.3 Hz, 3H). ^13^C-NMR (CDCl_3_) δ 134.4, 117.4, 109.8, 87.6, 81.3, 80.4, 78.5, 71.1, 69.1, 67.8, 31.3, 19.3, 13.8. HR-ESIMS *m*/*z* [M + H]^+^: calcd for C_13_H_23_O_5_: 259.1467; found: 259.1469.

#### 4.2.9. Synthesis of *Butyl 3,6-anhydro-2-O-nitro-5-O-allyl-α-d-glucofuranoside* (**2MNS**-**3**) and *Butyl 3,6-anhydro-2-O-nitro-5-O-allyl-β-d-glucofuranoside* (**2MNS**-**4**)

Fuming nitric acid (0.25 mL) was added to acetic anhydride (1 mL) at 0 °C. After stirring for 10 min, the solution of **9a** (60 mg, 0.23 mmol) in Ac_2_O (1 mL) was added into the acetic anhydride solution of fuming nitric acid. The mixture was stirred at 0 °C for 15 min and then poured into ice-water and extracted with EtOAc. The organic layer was washed with brine, dried with Na_2_SO_4_ and then concentrated under reduced pressure to give a crude product, which on column chromatography (hexane/EtOAc:20:1) gave **2MNS**-**3** (56 mg, 80%) as a colorless oil. [α]_D_^25^ = +129.68 (c = 0.11 in CHCl_3_); IR (neat, cm^−1^): 3441, 3335, 3131, 1660, 1640, 1612, 1454, 1400, 1282, 1138, 1121, 1070, 997, 955, 863, 619, 563, 538, 517; ^1^H-NMR (CDCl_3_) δ 6.04–5.87 (m, 1H), 5.46 (d, *J* = 4.2 Hz, 1H), 5.40–5.18 (m, 2H), 5.07–4.99 (m, 1H), 4.77–4.70 (m, 1H), 4.68–4.55 (m, 1H), 4.23–4.07 (m, 2H), 4.07–3.95 (m, 2H), 3.83–3.73 (m, 1H), 3.72–3.59 (m, 1H), 3.49–3.38 (m, 1H), 1.68–1.47 (m, 2H), 1.46–1.16 (m, 2H), 0.90 (t, *J* = 7.3 Hz, 3H). ^13^C-NMR (CDCl_3_) δ 134.1, 118.2, 102.0, 86.3, 82.9, 77.6, 76.7, 71.9, 68.4, 68.3, 31.2, 19.0, 13.6. HR-ESIMS *m*/*z* [M + H]^+^: calcd for C_13_H_22_NO_7_: 304.1318; found: 304.1321.

In a similar manner, **2MNS**-**4** (188 mg, 80% yield) was obtained as a colorless oil from **9b** (200 mg, 0.77 mmol). [α]_D_^25^ = +24.87 (c = 0.13 in CHCl_3_); IR (neat, cm^−1^): 3441, 3132, 1654, 1613, 1454, 1400, 1302, 1270, 1138, 1119, 1071, 994, 953, 845, 618, 564, 537, 517; ^1^H-NMR (CDCl_3_) δ 6.02–5.85 (m, 1H), 5.34 (q, *J* = 1.6 Hz, 1H), 5.26–5.18 (m, 2H), 5.15 (s, 1H), 4.81 (t, *J* = 4.8 Hz, 1H), 4.62 (d, *J* = 5.1 Hz, 1H), 4.25–4.13 (m, 1H), 4.08–3.96 (m, 3H), 3.95–3.84 (m, 2H), 3.47–3.37 (m, 1H), 1.65–1.53 (m, 2H), 1.43–1.33 (m, 2H), 0.92 (t, *J* = 7.3 Hz, 3H). ^13^C-NMR (CDCl_3_) δ 134.4, 117.6, 105.7, 90.0, 83.7, 81.7, 78.2, 71.3, 69.5, 68.2, 31.2, 19.3, 13.9. HR-ESIMS *m*/*z* [M + H]^+^: calcd for C_13_H_22_NO_7_: 304.1318; found: 304.1320.

#### 4.2.10. Synthesis of *Butyl 3,6-anhydro-2-O-nitro-α-d-glucofuranoside* (**2MNS**-**5**) and *Butyl 3,6-anhydro-2-O-nitro-β-d-glucofuranoside* (**2MNS**-**6**)

To a solution of **2MNS**-**3** (56 mg, 0.19 mmol) in methanol (5 mL) was added 10% Pd-C (18 mg) at room temperature. After being stirred for 5 h, The catalyst was filtered off and washed with methanol. The combined filtrate and the washings were condensed under reduced pressure to give a pale yellow oil, which was then subjected to column chromatography (hexane/EtOAc:20/1 → hexane/EtOAc:5/1), to provide **2MNS**-**5** (29 mg, 60%) as a colorless oil. [α]_D_^25^ = +120.83 (c = 0.11 in CHCl_3_); IR (neat, cm^−1^): 3440, 3132, 2454, 1660, 1638, 1616, 1454, 1400, 1283, 1259, 1138, 1120, 1069, 999, 954, 864, 817, 716, 619, 564, 537, 517; ^1^H-NMR (CDCl_3_) δ 5.46 (d, *J* = 4.1 Hz, 1H), 5.04 (t, *J* = 3.9 Hz, 1H), 4.74 (dd, *J* = 5.8, 3.6 Hz, 1H), 4.63 (t, *J* = 5.6 Hz, 1H), 4.30–4.19 (m, 1H), 3.99 (dd, *J* = 9.4, 5.8 Hz, 1H), 3.81–3.71 (m, 1H), 3.63 (dd, *J* = 9.4, 7.0 Hz, 1H), 3.49–3.38 (m, 1H), 2.41 (d, *J* = 8.0 Hz, 1H), 1.61–1.47 (m, 2H), 1.41–1.28 (m, 2H), 0.90 (t, *J* = 7.3 Hz, 3H). ^13^C-NMR (CDCl_3_) δ 102.6, 85.9, 82.6, 78.2, 72.0, 70.9, 68.7, 31.3, 19.1, 13.7. HR-ESIMS *m*/*z* [M + H]^+^: calcd for C_10_H_18_NO_7_: 264.1005; found: 264.1010.

In a similar manner, **2MNS**-**6** (78 mg, 60%) was obtained as a colorless oil from **2MNS**-**4** (150 mg, 0.49 mmol). [α]_D_^25^ = −4.10 (c = 0.12 in CHCl_3_); IR (neat, cm^−1^): 3440, 3132, 1655, 1616, 1454, 1400, 1305, 1271, 1137, 1120, 1069, 995, 954, 860, 618, 564, 537, 517, 447; ^1^H-NMR (CDCl_3_) δ 5.26 (s, 1H), 5.20 (s, 1H), 4.85 (t, *J* = 5.8 Hz, 1H), 4.56 (d, *J* = 5.6 Hz, 1H), 4.31–4.18 (m, 1H), 3.95–3.87 (m, 1H), 3.87–3.76 (m, 2H), 3.64–3.50 (m, 1H), 2.72 (d, *J* = 10.3 Hz, 1H), 1.80–1.50 (m, 2H), 1.45–1.35 (m, 2H), 0.94 (t, *J* = 7.3 Hz, 3H). ^13^C-NMR (CDCl_3_) δ 107.1, 89.6, 84.4, 83.4, 73.5, 71.2, 69.8, 31.4, 19.2, 13.8. HR-ESIMS *m*/*z* [M + H]^+^: calcd for C_10_H_18_NO_7_: 264.1005; found: 264.1008.

#### 4.2.11. Synthesis of *Butyl 3,6-anhydro-5-O-(3-nitro-4-methoxybenzyl)-2-O-nitro-α-d-glucofuranoside* (**2MNS**-**7**) and *Butyl 3,6-anhydro-5-O-(4-methoxybenzyl)-2-O-nitro-β-d-glucofuranoside* (**2MNS**-**8**)

Fuming nitric acid (0.5 mL) was added to acetic anhydride (2 mL) at 0 °C. After stirring for 10 min, the solution of **11a** (200 mg, 0.59 mmol) in Ac_2_O (2 mL) was added into the acetic anhydride solution of fuming nitric acid. The mixture was stirred at 0 °C for 15 min and then poured into ice-water and extracted with EtOAc. The organic layer was washed with brine, dried with Na_2_SO_4_ and then concentrated under reduced pressure to give a crude product, which on column chromatography (hexane/EtOAc:20:1) gave **2MNS**-**7** (205 mg, 81%) as a colorless oil. [α]_D_^25^ = +51.67 (c = 0.13 in CHCl_3_); IR (neat, cm^−1^):3440, 3132, 1640, 1616, 1534, 1454, 1400, 1282, 1138, 1070, 1017, 955, 863, 824, 623, 564, 537, 517. ^1^H-NMR (CDCl_3_) δ 7.89 (d, *J* = 2.2 Hz, 1H), 7.56 (dd, *J* = 8.6, 2.2 Hz, 1H), 7.09 (d, *J* = 8.6 Hz, 1H), 5.46 (d, *J* = 4.2 Hz, 1H), 5.04 (t, *J* = 3.7 Hz, 1H), 4.78–4.72 (m, 1H), 4.72–4.62 (m, 2H), 4.57 (d, *J* = 11.8 Hz, 1H), 4.09–3.92 (m, 5H), 3.82–3.62 (m, 2H), 3.51–3.37 (m, 1H), 1.60–1.46 (m, 2H), 1.40–1.26 (m, 2H), 0.90 (t, *J* = 7.3 Hz, 3H). ^13^C-NMR (CDCl_3_) δ 134.0, 125.7, 114.0, 102.7, 86.5, 83.5, 78.4, 77.1, 71.6, 69.0, 69.0, 57.0, 31.7, 19.5, 14.1. HR-ESIMS *m*/*z* [M + H]^+^: calcd for C_18_H_24_N_2_O_10_: 429.1431; found: 429.1435.

In a similar manner, **2MNS**-**8** (205 mg, 81%) was obtained as a colorless oil from **11b** (200 mg, 0.59 mmol). [α]_D_^25^ = +20.89 (c = 0.14 in CHCl_3_); IR (neat, cm^−1^):3442, 3132, 1743, 1649, 1621, 1534, 1461, 1400, 1302, 1269, 1138, 1119, 1071, 954, 861, 615, 564, 537, 517. ^1^H-NMR (CDCl_3_) δ 7.89 (d, *J* = 2.2 Hz, 1H), 7.54 (dd, *J* = 8.6, 2.3 Hz, 1H), 7.08 (d, *J* = 8.6 Hz, 1H), 5.24 (s, 1H), 5.17 (s, 1H), 4.85 (t, *J* = 5.0 Hz, 1H), 4.72 (d, *J* = 11.4 Hz, 1H), 4.64 (d, *J* = 5.1 Hz, 1H), 4.52 (d, *J* = 11.4 Hz, 1H), 4.14–3.88 (m, 6H), 3.89–3.79 (m, 1H), 3.49–3.37 (m, 1H), 1.65–1.48 (m, 2H), 1.40–1.28 (m, 2H), 0.88 (t, *J* = 7.3 Hz, 3H). ^13^C-NMR (CDCl_3_) δ 133.4, 130.2, 125.2, 113.5, 105.9, 90.00, 83.8, 81.5, 78.8, 70.6, 69.5, 68.4, 56.7, 31.2, 19.3, 13.8. HR-ESIMS *m*/*z* [M + H]^+^: calcd for C_18_H_24_N_2_O_10_: 429.1431; found: 429.1428.

#### 4.2.12. Synthesis of *Butyl 3,6-anhydro-2,5-di-O-nitro-α-d-arabinohexofuranoside* (**DNS**-**1**) and *Butyl 3,6-anhydro -2,5-di-O-nitro-β-d-arabinohexofuranoside* (**DNS**-**2**)

Fuming nitric acid (0.5 mL) was added to acetic anhydride (2 mL) at 0 °C. After stirring for 10 min, the solution of **12a** (100 mg, 0.46 mmol) in Ac_2_O (2 mL) was added into the acetic anhydride solution of fuming nitric acid. The mixture was stirred at 0 °C for 15 min and then poured into ice-water and extracted with EtOAc. The organic layer was washed with brine, dried with Na_2_SO_4_ and then concentrated under reduced pressure to give a crude product, which on column chromatography (hexane/EtOAc:20:1) gave **DNS**-**1** (113 mg, 80%) as a colorless oil. [α]_D_^25^ = +140.45 (c = 0.18 in CHCl_3_); IR (neat, cm^−1^): 3455, 3160, 1626, 1400, 1304, 1286, 1152, 1085, 1005, 985, 869, 844, 531; ^1^H-NMR (CDCl_3_) δ 5.44 (d, *J* = 4.0 Hz, 1H), 5.28 (q, *J* = 5.6 Hz, 1H), 5.08 (t, *J* = 3.9 Hz, 1H), 4.93 (t, *J* = 5.6 Hz, 1H), 4.81 (dd, *J* = 5.9, 3.7 Hz, 1H), 4.14 (dd, *J* = 10.4, 5.9 Hz, 1H), 3.98 (dd, *J* = 10.4, 5.6 Hz, 1H), 3.80–3.68 (m, 1H), 3.47–3.36 (m, 1H), 1.60–1.47 (m, 2H), 1.39–1.29 (m, 2H), 0.90 (t, *J* = 7.3 Hz, 3H). ^13^C-NMR (CDCl_3_) δ 103.2, 84.7, 83.2, 79.1, 77.5, 68.9, 68.2, 31.3, 19.1, 13.7. HR-ESIMS *m*/*z* [M + H]^+^: calcd for C_10_H_17_N_2_O_9_: 309.0858; found: 309.0856.

In a similar manner, **DNS**-**2** (107 mg, 76%) was obtained as a colorless oil from **12b** (100 mg, 0.46 mmol). [α]_D_^25^ = +57.22 (c = 0.12 in CHCl_3_); IR (neat, cm^−1^): 3448, 3133, 1648, 1457, 1400, 1303, 1283, 1121, 1071, 998, 952, 860, 564, 537, 516; ^1^H-NMR (CDCl_3_) δ 5.29–5.17 (m, 2H), 5.17–4.98 (m, 2H), 4.72 (d, *J* = 5.6 Hz, 1H), 4.21–4.06 (m, 2H), 3.92–3.81 (m, 1H), 3.50–3.38 (m, 1H), 1.67–1.53 (m, 2H), 1.45–1.34 (m, 2H), 0.94 (t, *J* = 7.3 Hz, 3H). ^13^C-NMR (CDCl_3_) δ 106.3, 89.6, 84.5, 81.3, 79.5, 68.9, 67.5, 31.2, 19.3, 13.9. HR-ESIMS *m*/*z* [M + H]^+^: calcd for C_10_H_17_N_2_O_9_: 309.0856; found: 309.0856.

### 4.3. Biology Evaluation

#### 4.3.1. Nitric Oxide Releasing Assay

NO has a short half-life, therefore, the quantification of NO metabolites like nitrite and nitrate is a useful method to quantify this molecule in the medium. The amount of NO released was indirectly detected by Griess reaction through the measurement of nitrites in the medium [33,34,35]. The solution of each nitrate derivative was prepared to 0.01 M. 0.1 mL of the solution of nitrate derivative was added into the incubator, and volume to 10 mL with 5 mM l-cysteine in phosphate buffer (pH 7.4). 150 μL of mixture was pipetted into reaction tube after 0 h, 4 h, 8 h, 16 h, 20 h, 24 h, 28 h, 32 h of incubation at 37 °C. Then 50 μL of the Griess reagent (4 g of sulfanilamide, 0.2 g of *N*-naphthyl-ethylenediamine dihydrochloride, 85% phosphoric acid (10 mL) in distilled water (final volume, 100 mL)) was added into above reaction tube. After 10 min at room temperature, the absorbance was measured at 540 nm. Standard sodium nitrite solutions (0.15 to 1.5 mg/L) were used to construct the calibration curve. No production of nitrite was observed in the absence of L-cysteine. The yields of nitrite are expressed as NO_2_^−^ (μM). The experiments were performed in sextuplicate in three independent experiments. Data are expressed as the mean ± SD.

#### 4.3.2. Vasodilatory Potential on Isolated Rat Mesenteric Arterial Rings

Adult male Sprague-Dawley rats (200–300 g, 2 months of age) were obtained from Experimental Animal Center of Shanghai Institute of Materia Medica (Shanghai, China). Male rats that had typical 4-day estrous cycles were used in the present study. Experimental animals, which were housed in a group of 4 per wire cage, were kept under standard laboratory conditions (12 h of light, 12 h of dark; 30 °C) for a week to acclimatize to laboratory conditions. During acclimatization, animals were provided food and water ad libitum. Vaginal smears were checked twice-daily (9 a.m. and 8 p.m.) to characterize the estrous cycle.

After rats were euthanized by cervical dislocation, the gastrointestinal tract with the mesenteric arcade attached was excised rapidly and kept in ice-cold physiological salt solution (PSS) with the following composition (mmol/L):NaCl 130, KCl 4.7, MgSO_4_ 1.17, KH_2_PO_4_ 1.18, NaHCO_3_ 14.9, glucose 5.5, and ethylene diamine tetraacetic acid 0.026. Second-order mesenteric small arteries (<400 μM internal diameter) were dissected and cleaned of adjoining fat and connective tissues [49].

The isolated mesenteric arteries of male Sprague-Dawley rats (200–300 g) were cut into ring segments of 3–4 mm in length. Each segment was mounted in a Powerlab isolated tissue perfusion system (AD Instruments, Dunedin, New Zealand) and the contractile responses were determined. The organ chamber was filled with K-H solution, which was constantly bubbled with 95% O_2_/5% CO_2_ and maintained at 37 °C. After an equilibration period of 60 min, the endothelial integrity was confirmed by acetylcholine (Ach, 1 μM)-induced relaxation of phenylephrine (1 μM) precontracted tissues. A relaxation rate >70% of the phenylephrine-induced contraction was considered representative of an acceptable presence of the endothelial layer, while the organs, showing a relaxation rate <70%, were not used in the experimental procedures [50]. 30 to 40 min after the confirmation of the endothelial integrity, all rings were used in next experiences. The vascular preparation was normalized to the optimal initial tension at 0.5 g in accordance with the system instructions. All rings were allowed to stabilize at this baseline tone for 60 min before the start of each experiment.

The vasodilatory effect was reflected by the change of tension of isolated rat mesenteric arterial ring. The contraction that induced by 1 μM phenylephrine or 60 mM KCl was indentified as standard contraction. The relaxation rate of each nitrate derivative was used as the effect that nitrate derivative on isolated rat mesenteric arterial ring. Relaxation rate (%) = (1 − average minimum tension/average initial tension) × 100%. The higher relaxation rate, the more potent vasodilatory effect. All the experiments were performed in triplicate in three independent experiments. Data are expressed as the mean ± SD.

Mesenteric artery rings pre-contracted with 1 μM phenylephrine. After a sustained contraction was obtained, nitrate derivative was added to the bathing solution at final concentration of 30 μM to obtain preliminary vasdilatory effect.

Mesenteric artery rings pre-contracted with 1 μM phenylephrine. After a sustained contraction was obtained, nitrate derivative was added to the bathing solution in a cumulative manner (0, 1, 3, 5, 10, 15, 20, 25, 30, 40, 50, 60, 70 μM) to obtain concentration-response curves. Each concentration was applied for 5 min before addition of the next concentration. DMSO was used as a control to ensure a genuine relaxant effect of nitrate derivative. Dose response curve was plotted using the GraphPad Prism 5.0 software (GraphPad Software, Inc., San Diego, CA, USA).

Mesenteric artery rings pre-contracted with 60 mM KCl. After a sustained contraction was obtained, nitrate derivative was added to the bathing solution at final concentration of 30 μM to obtain preliminary vasdilatory effect.

Mesenteric artery rings pre-contracted with 60 mM KCl. After a sustained contraction was obtained, nitrate derivative was added to the bathing solution in a cumulative manner (0, 1, 3, 5, 10, 15, 20, 25, 30, 40, 50, 60, 70 μM) to obtain concentration-response curves. Each concentration was applied for 5 min before addition of the next concentration. DMSO was used as a control to ensure a genuine relaxant effect of nitrate derivative. Dose response curve was plotted using the GraphPad software.

#### 4.3.3. Effects of ODQ and PITO on Vasodilatory Effects of DNS-2

Mesenteric artery rings were contracted by 1 μM phenylephrine. After a sustained contraction was obtained, three groups of contracted rings were treated with PTIO (100 μM), ODQ (10 μM) and **DNS**-**2**, respectively. Other two groups of contracted rings were pretreated PTIO (100 μM) or ODQ (10 μM) for 15 min, and then treated with **DNS**-**2** (final concentration of 30 μM). ISDN instead of **DNS**-**2** was in presence of positive control experience. Relaxation rate (%) = (1 − average minimum tension/average initial tension) × 100%. The experiments were performed in triplicate in three independent experiments. Data are expressed as the mean ± SD.

### 4.4. Statistical Analysis

All the data were represented as mean ± SD from triplicate experiments performed in a parallel manner unless otherwise indicated. Statistical analysis of comparisons between two groups was analyzed using an unpaired, two-tailed Student’s *t*-test. Difference at the *p* < 0.05 level was considered statistically significant.

## 5. Conclusions

In summary, a group of nitrate derivatives of naturally occurring sauropunol A and B were designed and synthesized. Biological evaluation of these compounds revealed that most of synthesized mononitrate derivatives demonstrated superior NO releasing capacity and vasodilatory effect than ISMN. Dinitrate **DNS**-**2** presenting higher NO releasing capacity and vasodilatory effect than ISDN was found as the most ideal sauropunol-derived nitrate. High levels of NO and sGC may be essential for the potent vasodilatory effect of **DNS**-**2**. **DNS**-**2** may display vasodilatory effects through cellular signal transduction of NO-sGC-cGMP. The above findings are believed to be not only important for the extension of potential pharmaceutical application of naturally occurring sauropunols, but also for identification of new NO-releasing agents with structurally diversified backbones. Based on these results, further SAR studies to develop new sauropunol-type vasodilatory agents with higher efficacy and safety profile is underway in our laboratory.

## Figures and Tables

**Table 1:** Inhibitory effects of nitrate derivatives (30 μM) on the contractions induced by phenylephrine or KCl in mesenteric artery rings.

Compounds	Relaxation ^a^ (%)	Relaxation ^b^ (%)
ISMN	8.01 ± 3.59	3.05 ± 1.69
ISDN	30.21 ± 2.55	20.13 ± 3.45
5MNS-1	58.13 ± 3.01 **^##^	24.21 ± 2.79 **
5MNS-2	30.21 ± 2.90 **	15.15 ± 3.01 **
5MNS-3	45.38 ± 3.51 **^##^	16.09 ± 2.01 **
5MNS-4	20.11 ± 4.73 *^#^	11.86 ± 3.56 *^#^
5MNS-5	4.10 ± 2.01 ^##^	2.01 ± 1.00 ^##^
2MNS-1	15.22 ± 3.02 ^##^	7.08 ± 2.00 ^##^
2MNS-2	9.89 ± 2.71 ^##^	4.96 ± 3.54 ^##^
2MNS-3	31.03 ± 2.66 **	5.06 ± 1.79 ^##^
2MNS-4	51.25 ± 5.02 **^##^	22.89 ± 2.53 **
2MNS-5	11.00 ± 2.65 ^##^	2.02 ± 1.03 ^##^
2MNS-6	8.96 ± 2.06 ^##^	52.11 ± 3.66 **^##^
2MNS-7	22.1 ± 4.43 *	86.27 ± 2.37 **^##^
2MNS-8	67.56 ± 3.86 **^##^	85.17 ± 4.54 **^##^
DNS-1	29.21 ± 3.66 **	80.87 ± 5.31 **^##^
DNS-2	81.98 ± 6.10 **^##^	85.22 ± 6.01 **^##^

^a^ Inhibitory effects of nitrate derivatives (30 μM) on the contractions induced by phenylephrine (1 μM) in mesenteric artery rings. ^b^ Inhibitory effects of nitrate derivatives (30 μM) on the contractions induced by of KCl (60 mM) in mesenteric artery rings. Data are expressed as the mean ± SD (*n* = 3). * *p* < 0.05, ** *p* < 0.01 vs. ISMN, ^#^ *p* < 0.05, ^##^ *p* < 0.01 vs. ISDN.

**Table 2:** IC_50_ of nitrate derivatives on the contractions induced by phenylephrine or KCl in mesenteric artery rings.

Compounds	IC_50_^a^ (μM)	Compounds	IC_50_^b^ (μM)
ISMN	42.30 ± 3.52	ISMN	35.34 ± 2.52
ISDN	13.86 ± 0.56	ISDN	16.93 ± 0.98
5MNS-1	24.25 ± 0.50 **^##^	2MNS-6	32.08 ± 6.40 ^#^
5MNS-3	36.16 ± 1.37 *^##^	2MNS-7	5.94 ± 0.42 **^##^
2MNS-3	12.08 ± 0.65 **^#^	2MNS-8	5.52 ± 0.47 **^##^
2MNS-4	13.75 ± 1.00 **	DNS-1	11.14 ± 1.29 **^##^
2MNS-8	6.94 ± 0.72 **^##^	DNS-2	10.03 ± 0.72 **^##^
DNS-2	6.02 ± 0.40 **^##^		

^a^ IC_50_ of nitrate derivatives on the contractions induced by phenylephrine in mesenteric artery rings. ^b^ IC_50_ of nitrate derivatives on the contractions induced by KCl in mesenteric artery rings. Data are expressed as the mean ± SD (*n* = 3). * *p* < 0.05, ** *p* < 0.01 vs. ISMN, ^#^ *p* < 0.05, ^##^ *p* < 0.01 vs. ISDN.

## Supplementary Materials

The following are available online, ^1^H- and ^13^C-NMR spectra for all tatgeted compounds and 7a, 7b, 8, 9a, 9b. IR spectra for all tatgeted compounds and 8, 9a, 9b. Figure S1: The inhibitory effect of nitrates derivatives on the contraction induced by phenylephrine in mesenteric artery rings. Figure S2: The inhibitory effect of nitrates derivatives on the contraction induced by KCl in mesenteric artery rings.
