# Supplementary material for: Design, Synthesis and Biological Evaluation of Nitrate Derivatives of Sauropunol A and B as Potent Vasodilatory Agents
Source: Molecules. 2019 Feb 6;24(3):583. doi: 10.3390/molecules24030583 (PMC6384914; doi:10.3390/molecules24030583)
Supplement: Supplementary file 1 [file molecules-24-00583-s001.pdf]

Supplementary Material

Design, synthesis and biological evaluation of nitrate derivatives of sauropunol A and B as potent vasodilatory agents

Lu Lu [1], Xuemin Rao [1], Rigang Cong [2], Chenxi Zhang [1], Zhimei Wang [1], Jinyi Xu [1], Genzoh Tanabe [3], Osamu Muraoka [3], Xiaoming Wu [1]* and Weijia Xie [1]*

[1] State Key Laboratory of Natural Medicines and Department of Medicinal Chemistry, China Pharmaceutical University, 24 Tong Jia Xiang, Nanjing 210009, PR China;

[2] National-certified Enterprise Technology Center; Disha Pharmaceutical Group Co., Ltd;

[3] Faculty of Pharmacy, Kinki University, 3-4-1 Kowakae, Higashi-Osaka, Osaka 577-8502, Japan;

*Corresponding authors:

*E-Mail: xmwu@cpu.edu.cn (Xiaoming Wu),

*E-mail: weijiaxie@cpu.edu.cn (Weijia Xie); Tel.: +86-25-8327-1414 (Weijia Xie).

………

Contents

1. Copies of ^{1}H NMR and ^{13}C NMR……………………..……………………..………..2
2. Copies of IR……..………………………..……………………………..………….....21
3. The inhibitory effect of nitrates derivatives on the contraction induced by phenylephrine in mesenteric artery rings……………………...31
4. The inhibitory effect of nitrates derivatives on the contraction induced by KCl in mesenteric artery rings……………………………..31

1. Copies of ^{1}H NMR and ^{13}C NMR

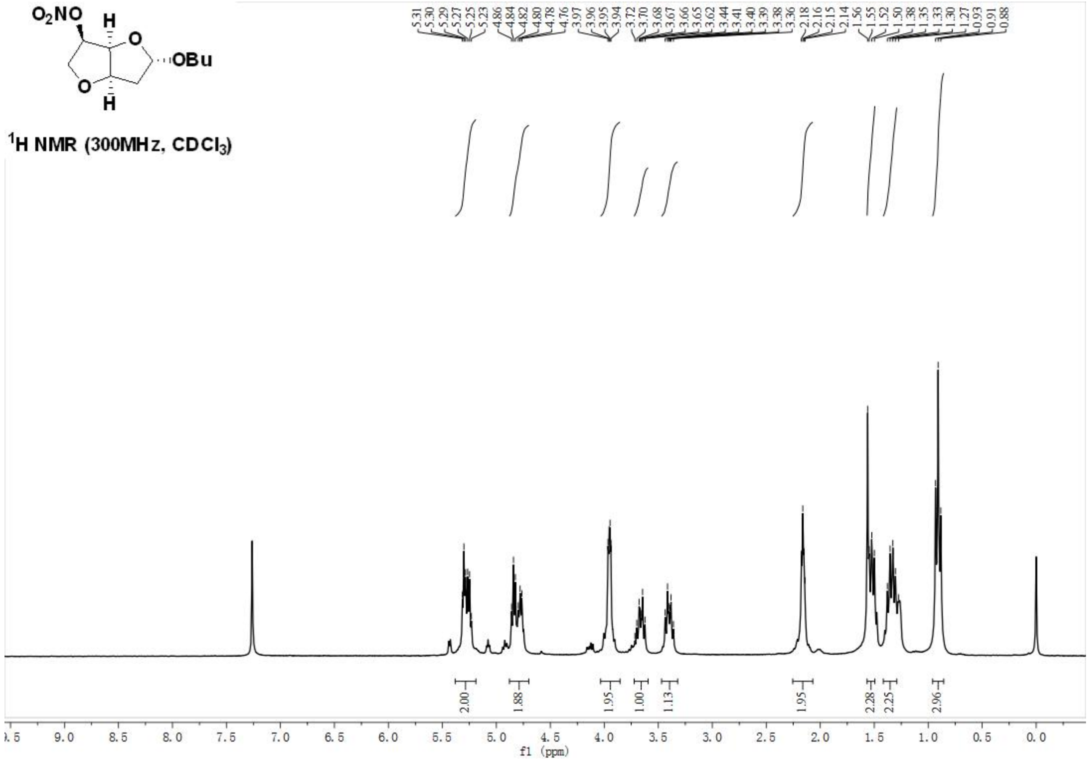

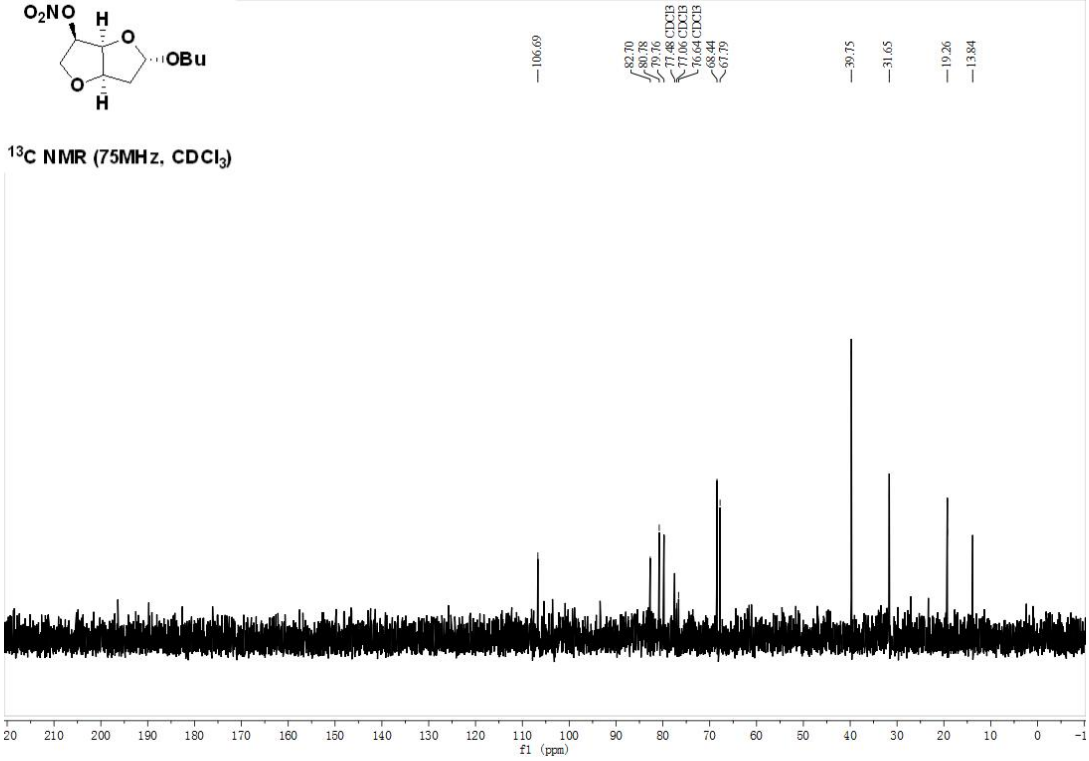

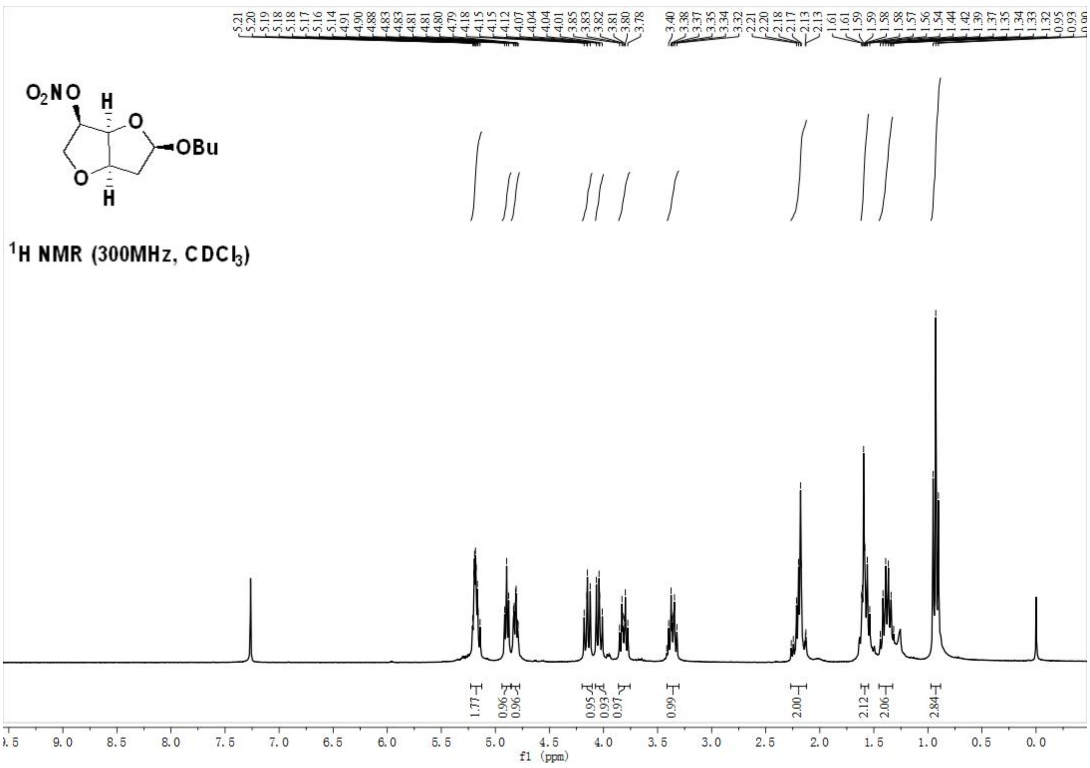
O2NO
H
O
OBu
O
H
1H NMR (300MHz, CDCl3)
1.77
0.96
0.96
0.95
0.93
0.97
0.99
2.00
2.12
2.06
2.84
f1 (ppm)

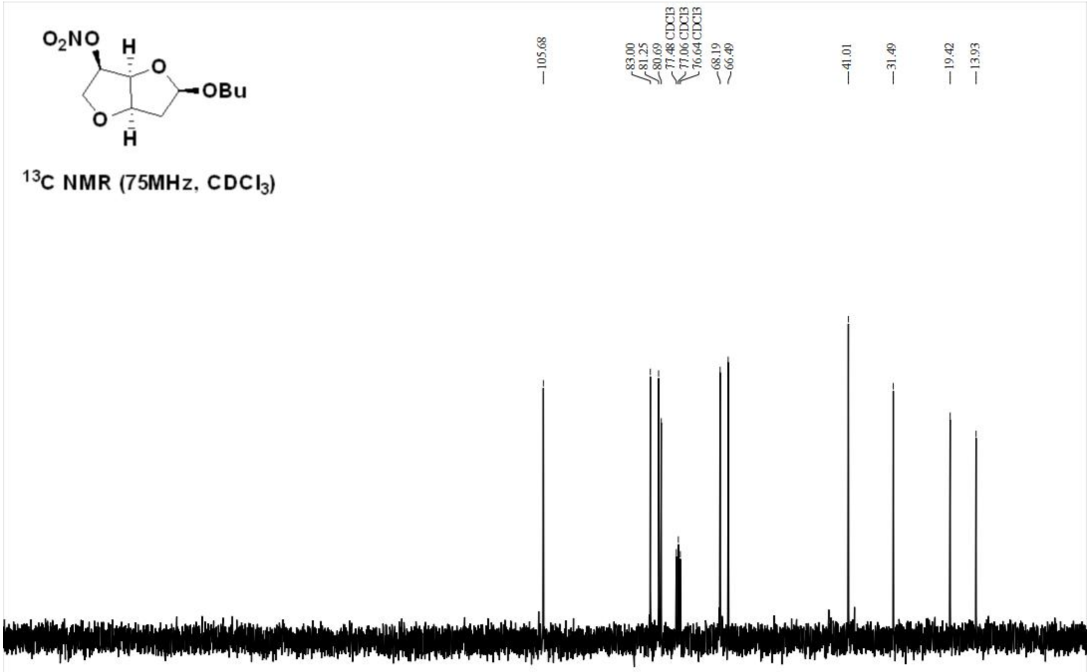
O2NO
H
O
OBu
O
H
13C NMR (75MHz, CDCl3)
105.68
83.00
81.25
80.69
77.48 CDCl3
77.06 CDCl3
76.64 CDCl3
68.19
66.49
41.01
31.49
19.42
13.93
f1 (ppm)

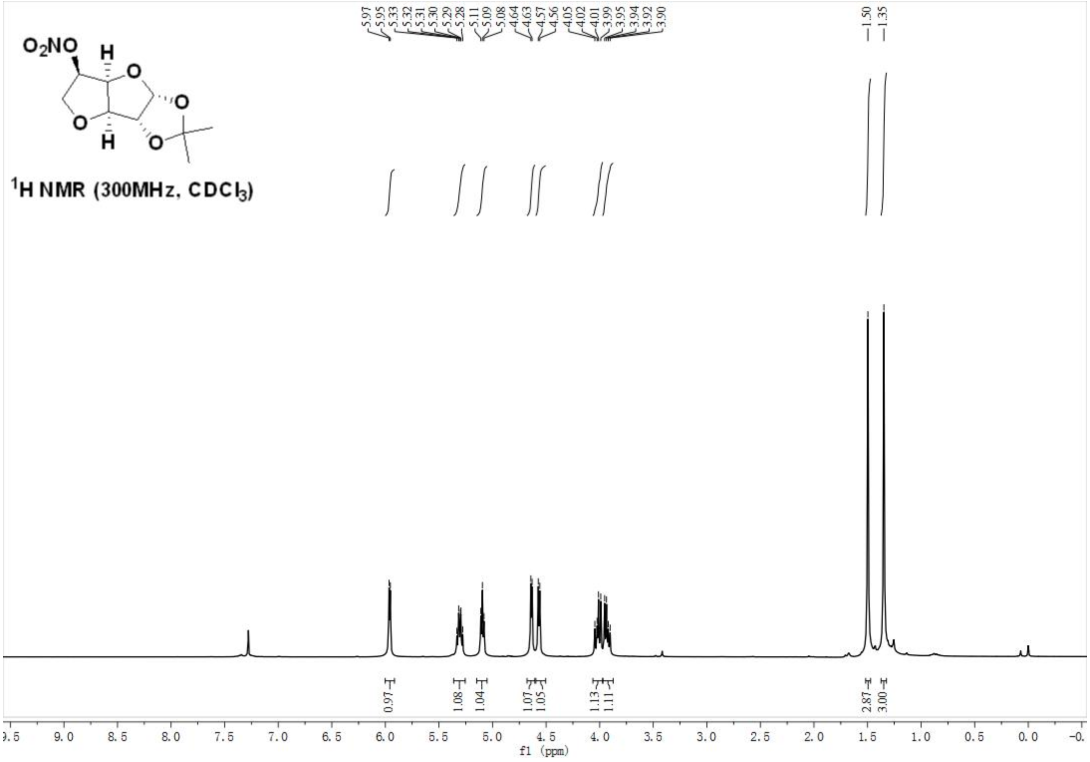
O2NO
H
O
O
O
H
O
1H NMR (300MHz, CDCl3)
5.97
5.95
5.33
5.32
5.31
5.30
5.29
5.28
5.11
5.09
5.08
4.64
4.63
4.57
4.56
4.05
4.02
4.01
3.99
3.95
3.94
3.92
3.90
1.50
1.35
0.97
1.08
1.04
1.07
1.05
1.13
1.11
2.87
3.00
f1 (ppm)

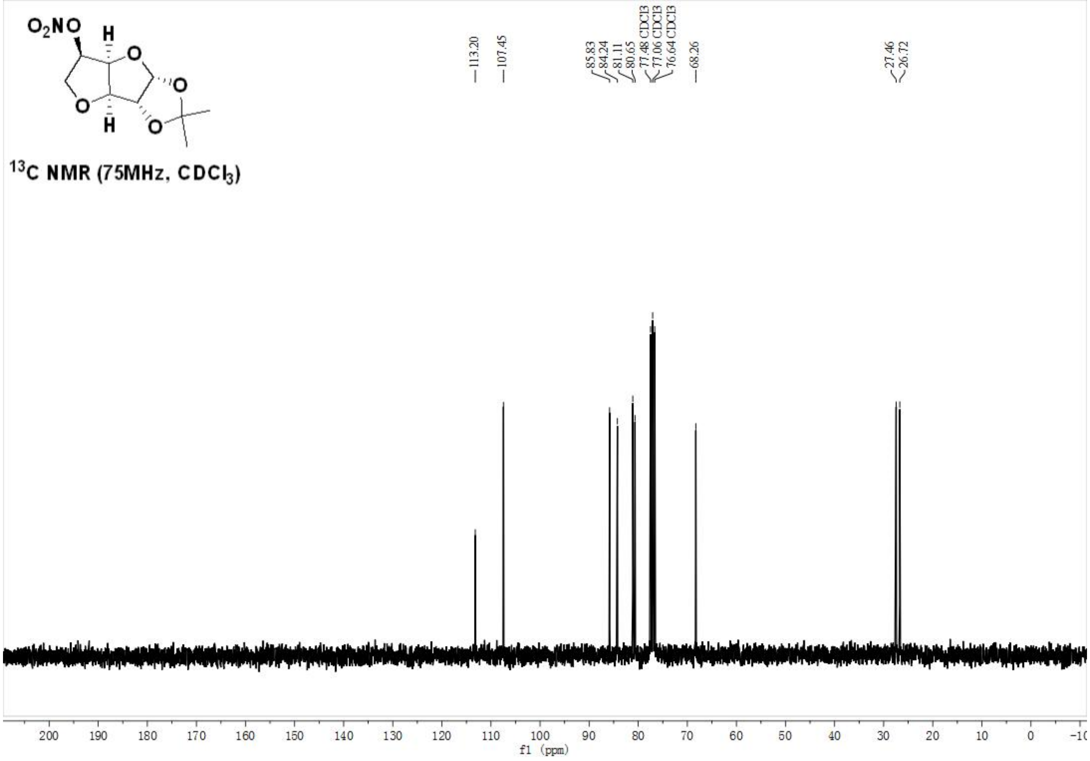
O2NO
H
O
O
O
H
O
13C NMR (75MHz, CDCl3)
113.20
107.45
85.83
84.24
81.11
80.65
77.48 CDCl3
77.06 CDCl3
76.64 CDCl3
68.26
27.46
26.72
f1 (ppm)

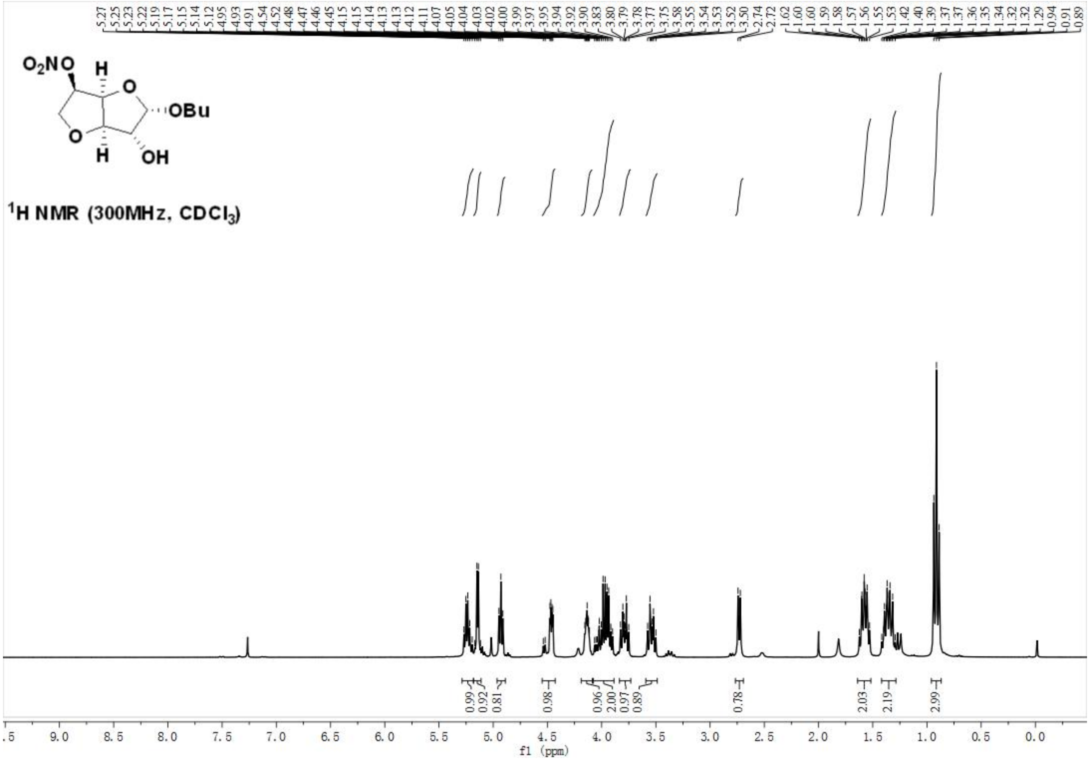
O2NO
H
O
OBu
O
H
OH
1H NMR (300MHz, CDCl3)
f1 (ppm)

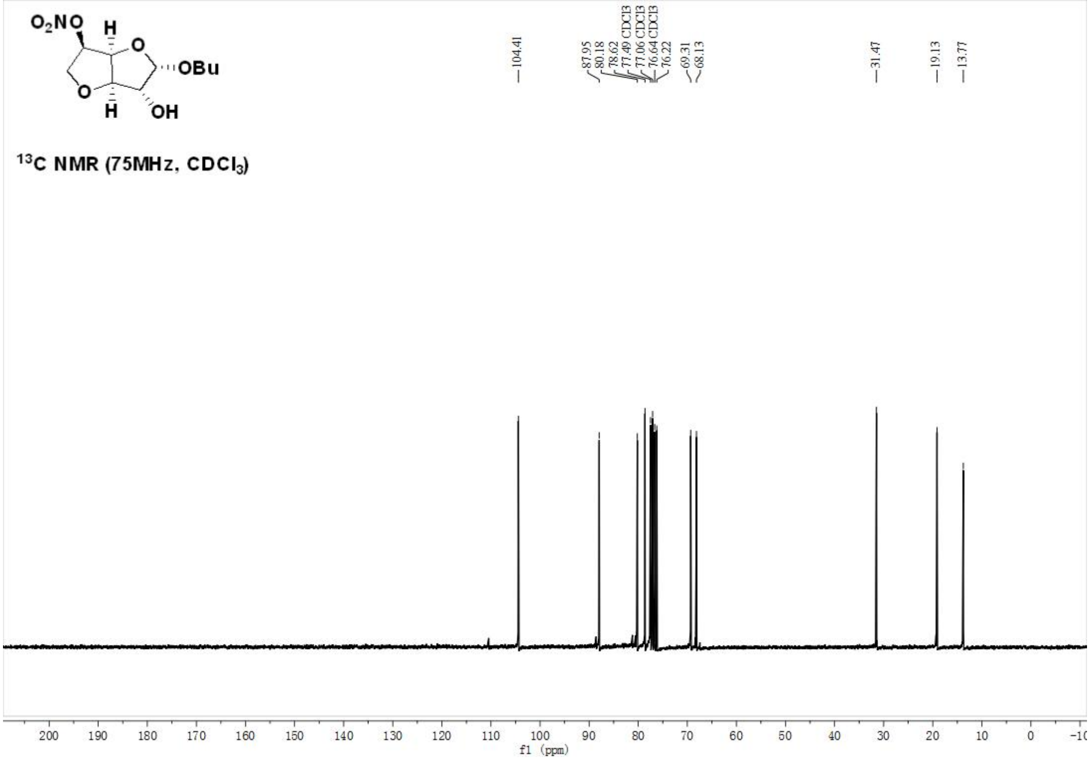
O2NO
H
O
OBu
O
H
OH
13C NMR (75MHz, CDCl3)
104.41
87.95
80.18
78.62
77.49 CDCl3
77.06 CDCl3
76.64 CDCl3
76.22
69.31
68.13
31.47
19.13
13.77
f1 (ppm)

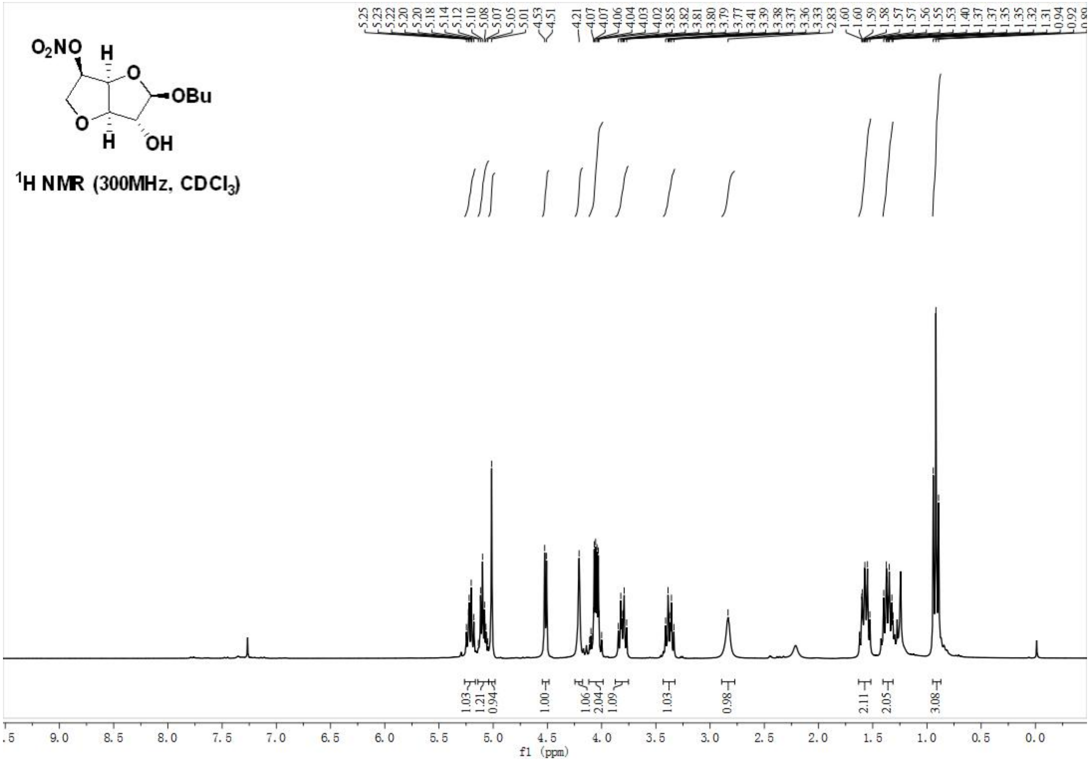
O2NO
OBu
OH
1H NMR (300MHz, CDCl3)
f1 (ppm)

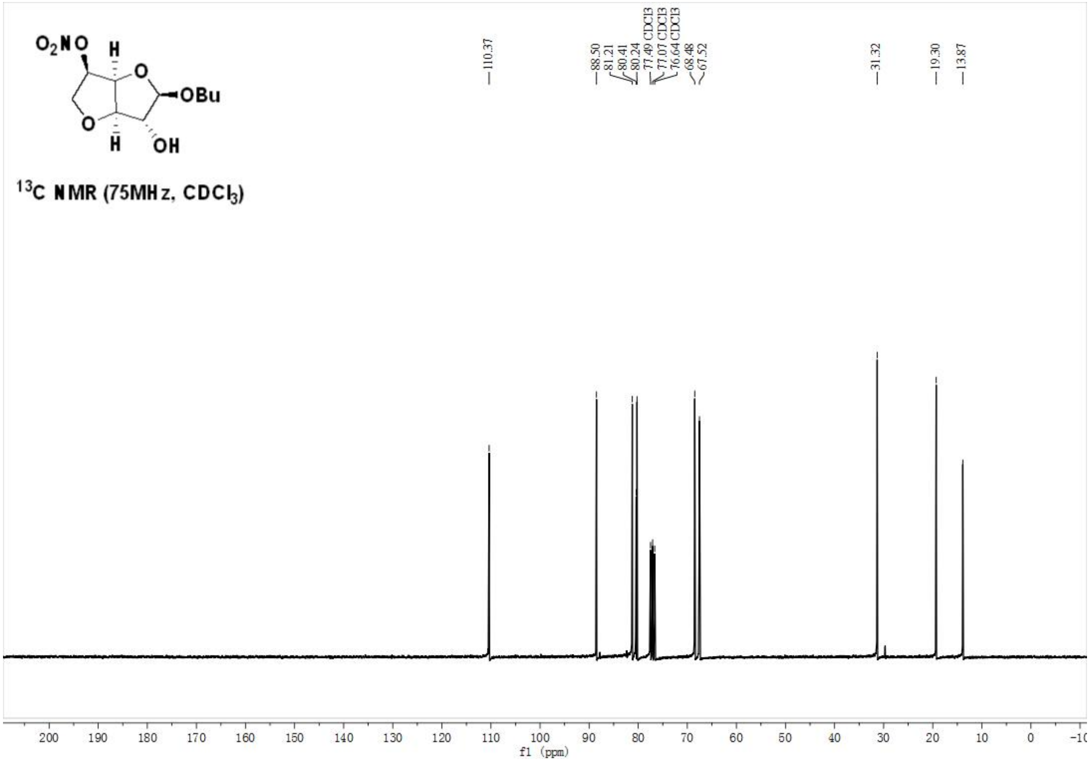
O2NO
OBu
OH
13C NMR (75MHz, CDCl3)
110.37
88.50
81.21
80.41
80.24
77.49 CDCl3
77.07 CDCl3
76.64 CDCl3
68.48
67.52
31.32
19.30
13.87
f1 (ppm)

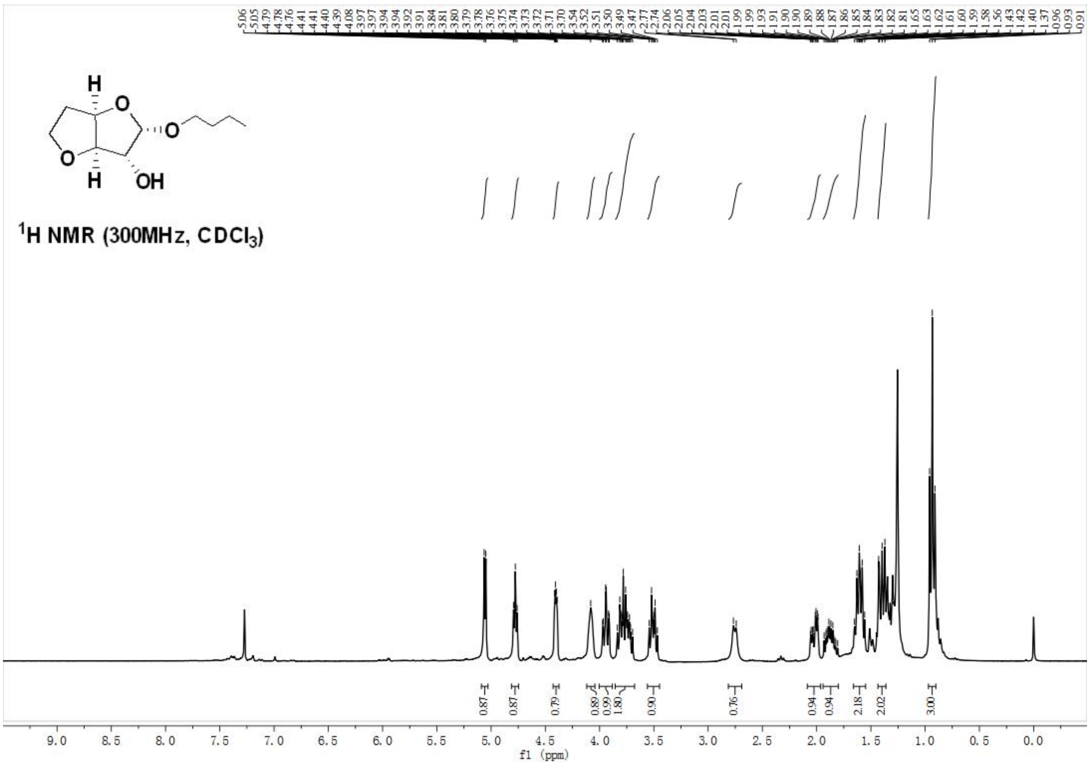

H
O
O
OH
H
^{1}H NMR (300MHz, CDCl$_3$)
0.87
0.87
0.79
0.89
0.99
1.80
0.90
0.76
0.94
0.94
2.18
2.02
3.00
9.0 8.5 8.0 7.5 7.0 6.5 6.0 5.5 5.0 4.5 4.0 3.5 3.0 2.5 2.0 1.5 1.0 0.5 0.0
f1 (ppm)

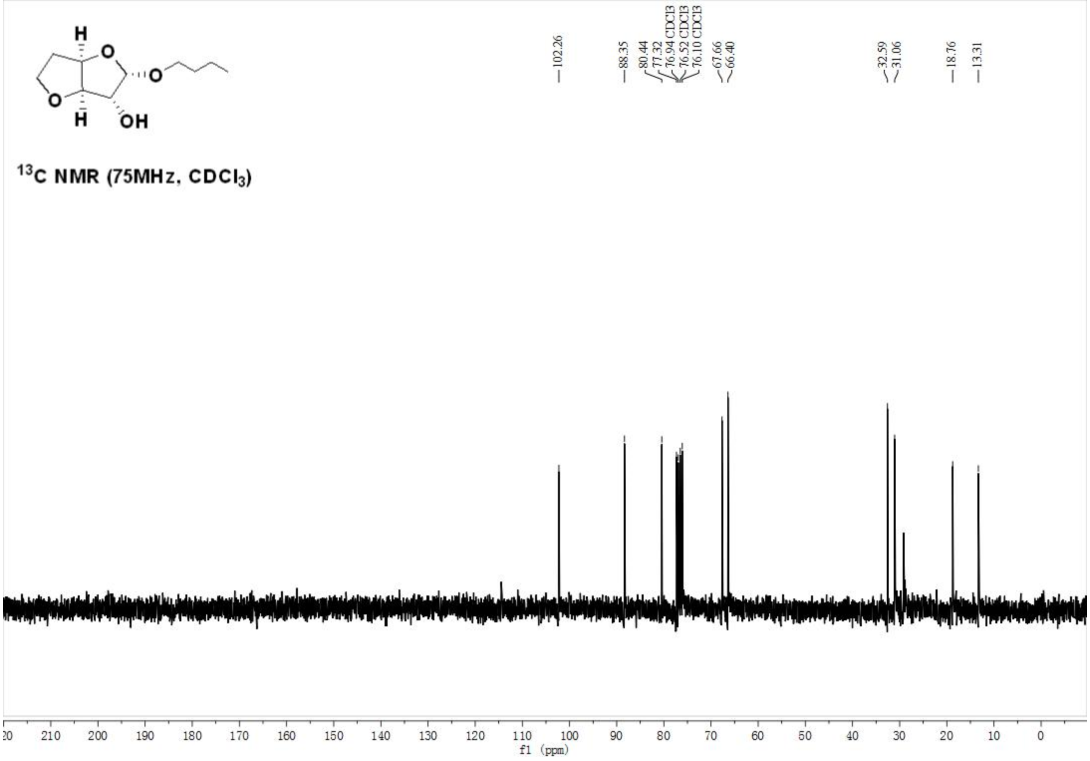

H
O
O
OH
H
^{13}C NMR (75MHz, CDCl$_3$)
102.26
88.35
80.44
77.32 CDCl3
76.94 CDCl3
76.52 CDCl3
76.10 CDCl3
67.66
66.40
32.59
31.06
18.76
13.31
210 200 190 180 170 160 150 140 130 120 110 100 90 80 70 60 50 40 30 20 10 0
f1 (ppm)

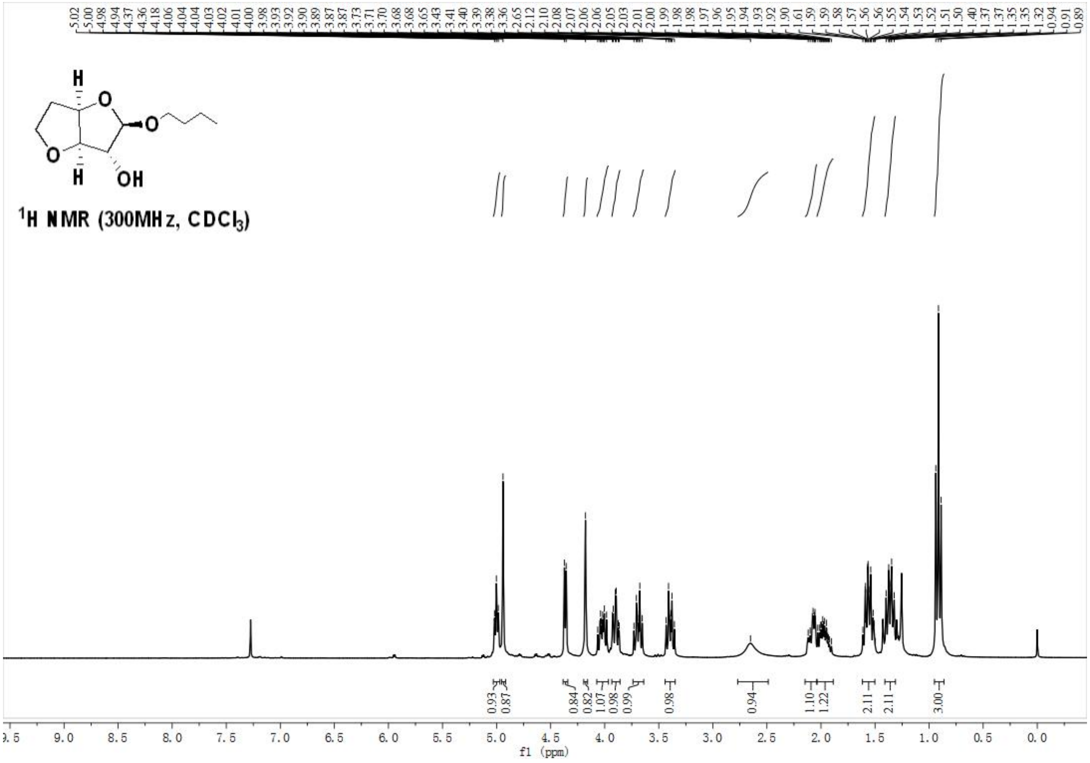
^{1}H NMR (300MHz, CDCl$_3$)

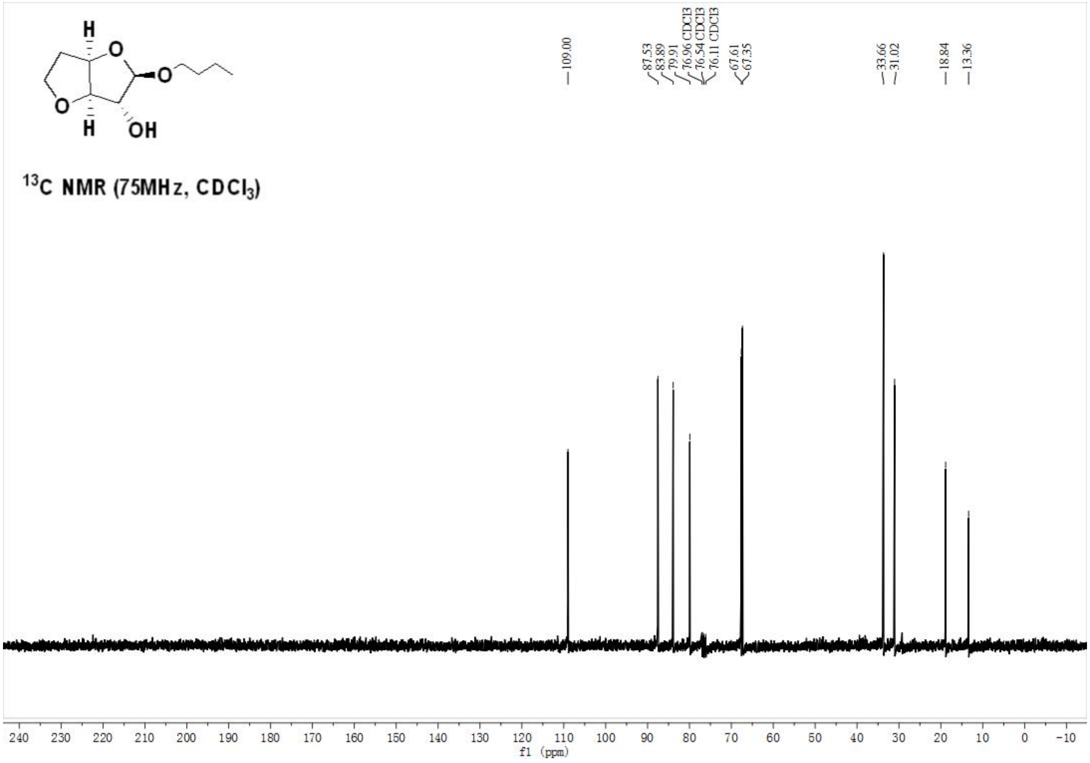
^{13}C NMR (75MHz, CDCl$_3$)

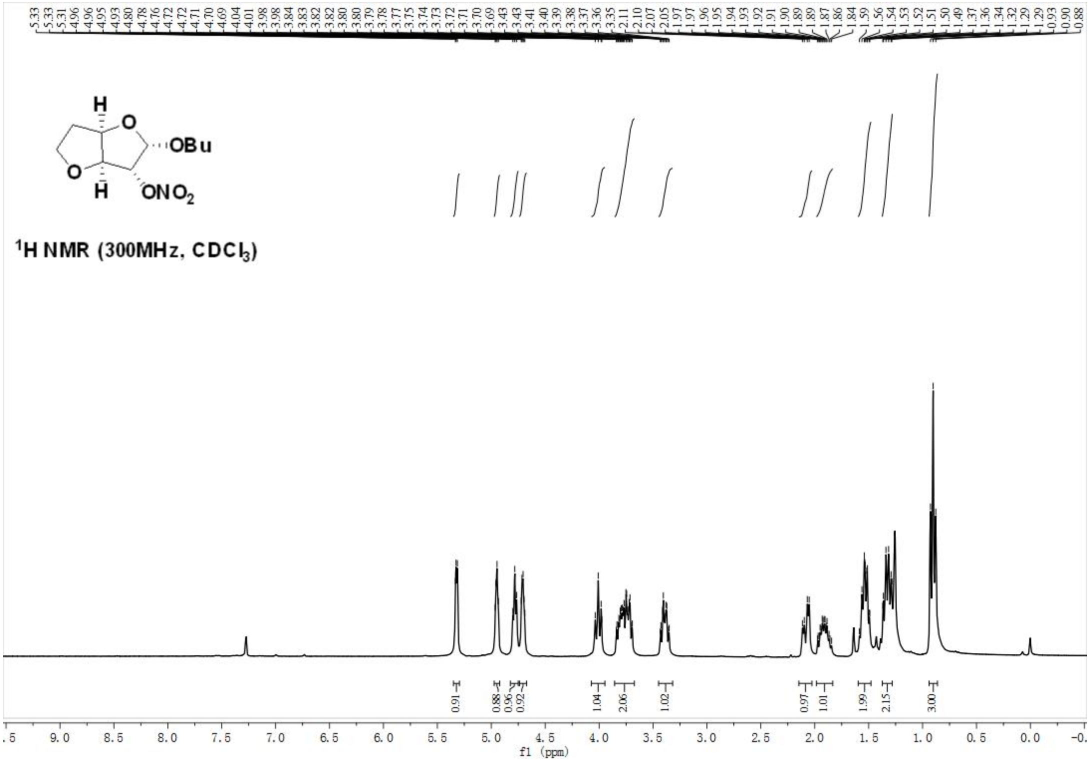
OBu
ONO_2
^{1}H NMR (300MHz, $CDCl_3$)
f1 (ppm)

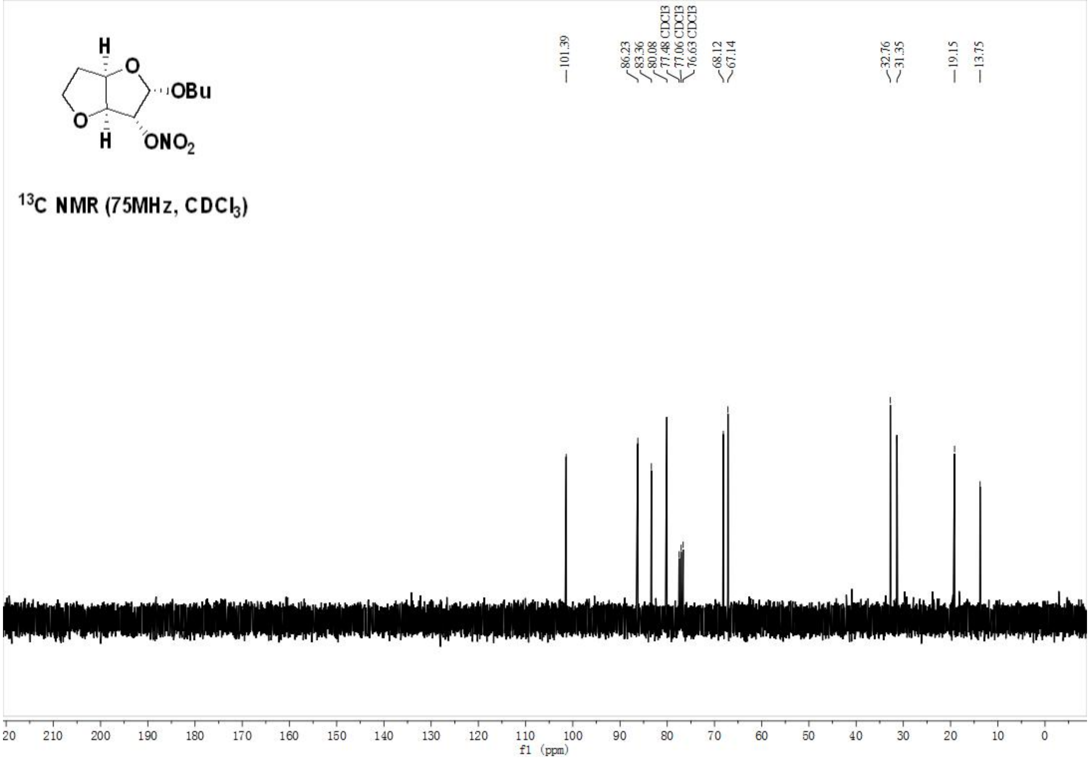
OBu
ONO_2
^{13}C NMR (75MHz, $CDCl_3$)
101.39
86.23
83.36
80.08
77.48 CDCl3
77.06 CDCl3
76.63 CDCl3
68.12
67.14
32.76
31.35
19.15
13.75
f1 (ppm)

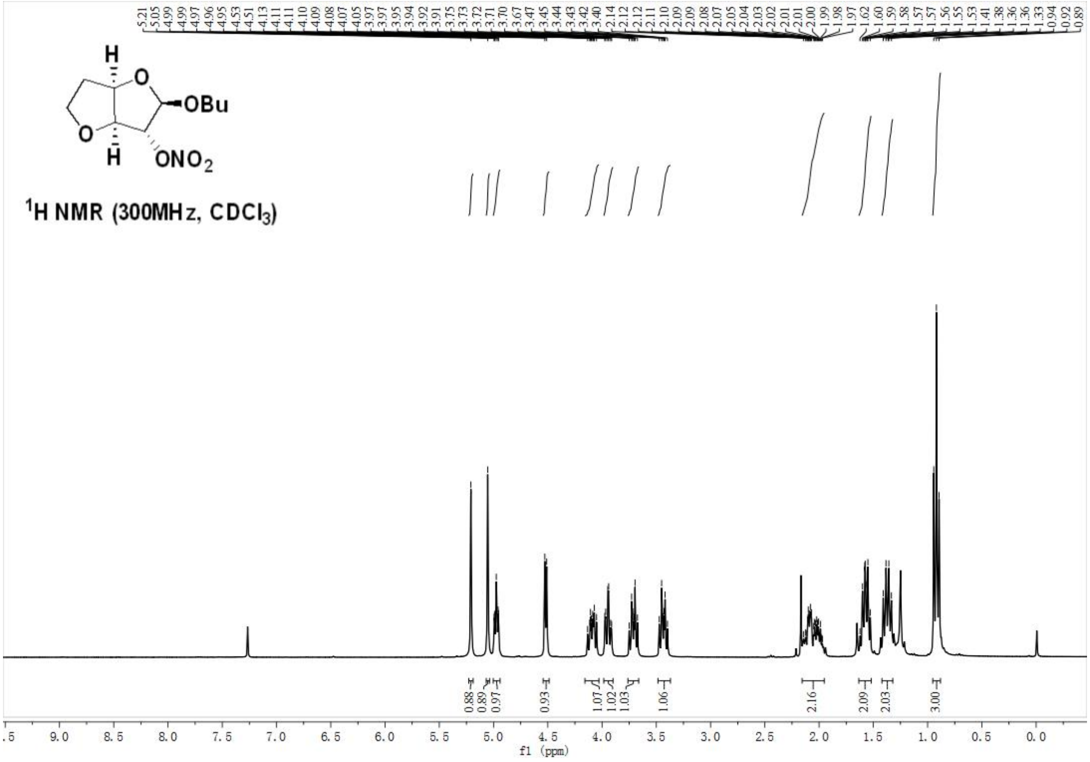
H
O
OBu
O
H
ONO2
1H NMR (300MHz, CDCl3)
f1 (ppm)

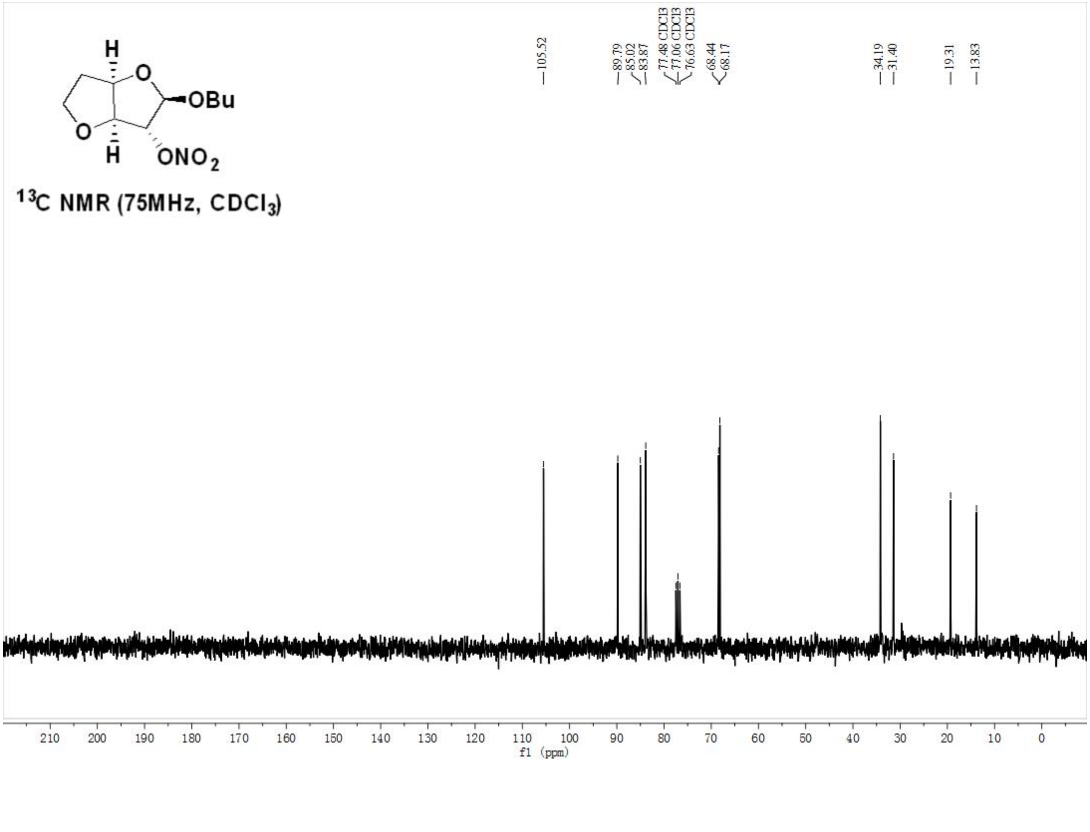
105.52
89.79
85.02
83.87
77.48 CDCl3
77.06 CDCl3
76.63 CDCl3
68.44
68.17
34.19
31.40
19.31
13.83
H
O
OBu
O
H
ONO2
13C NMR (75MHz, CDCl3)
f1 (ppm)

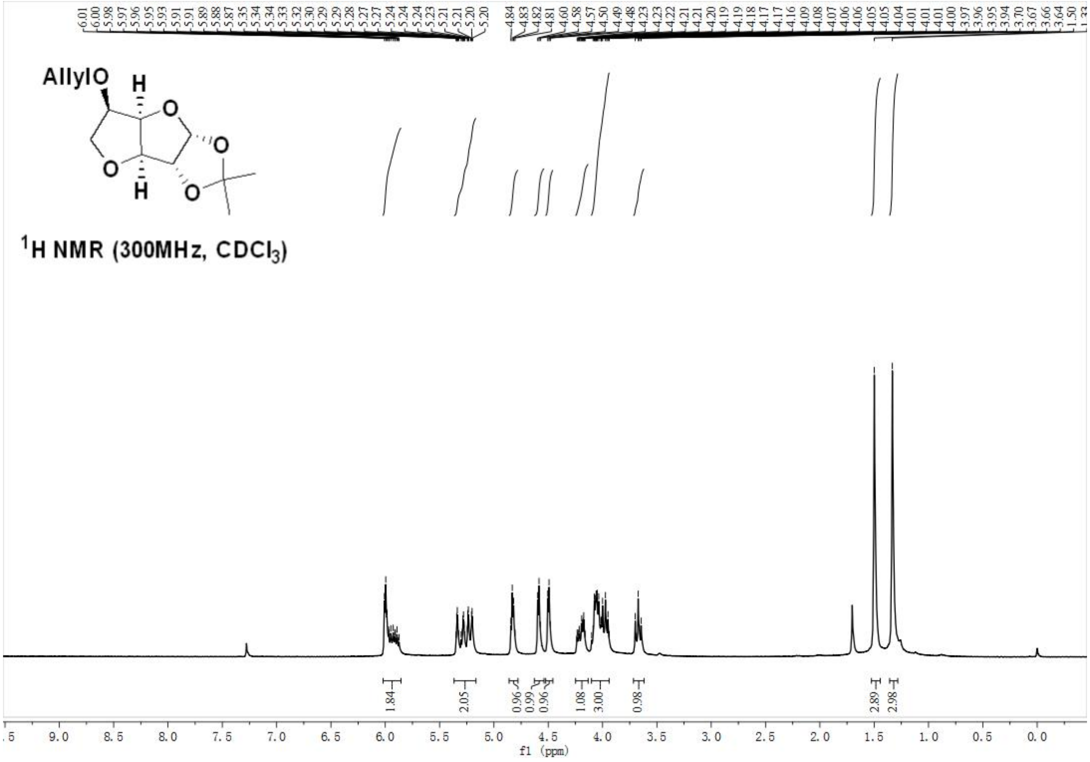
AllylO
1H NMR (300MHz, CDCl3)
1.84
2.05
0.96
0.99
0.96
1.08
3.00
0.98
2.89
2.98
f1 (ppm)

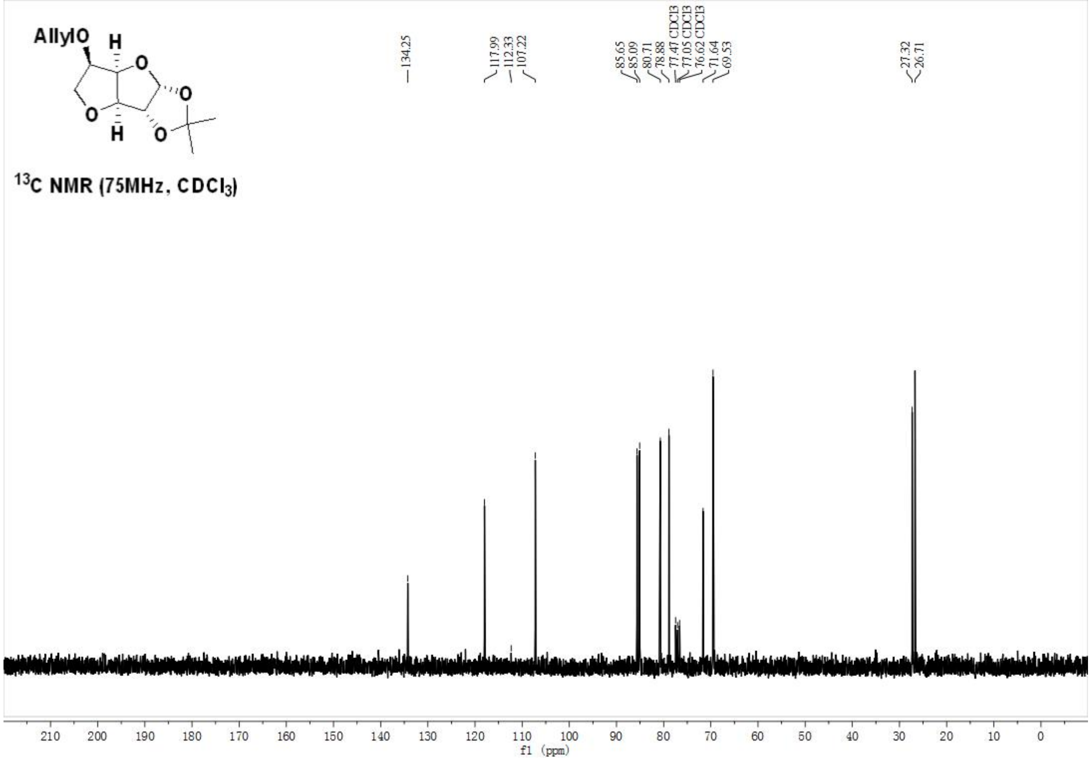
AllylO
13C NMR (75MHz, CDCl3)
134.25
117.99
112.33
107.22
85.65
85.09
80.71
78.88
77.47 CDCl3
77.05 CDCl3
76.62 CDCl3
71.64
69.53
27.32
26.71
f1 (ppm)

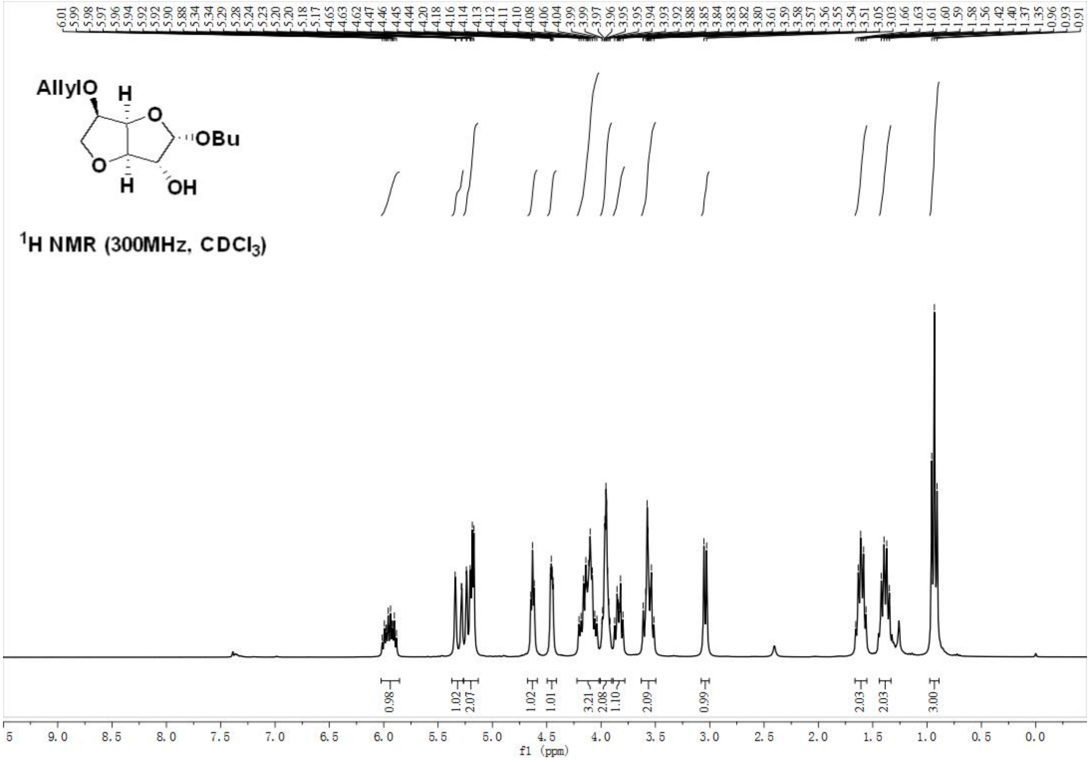
AllylO
H
O
OBu
O
H
OH
1H NMR (300MHz, CDCl3)
f1 (ppm)

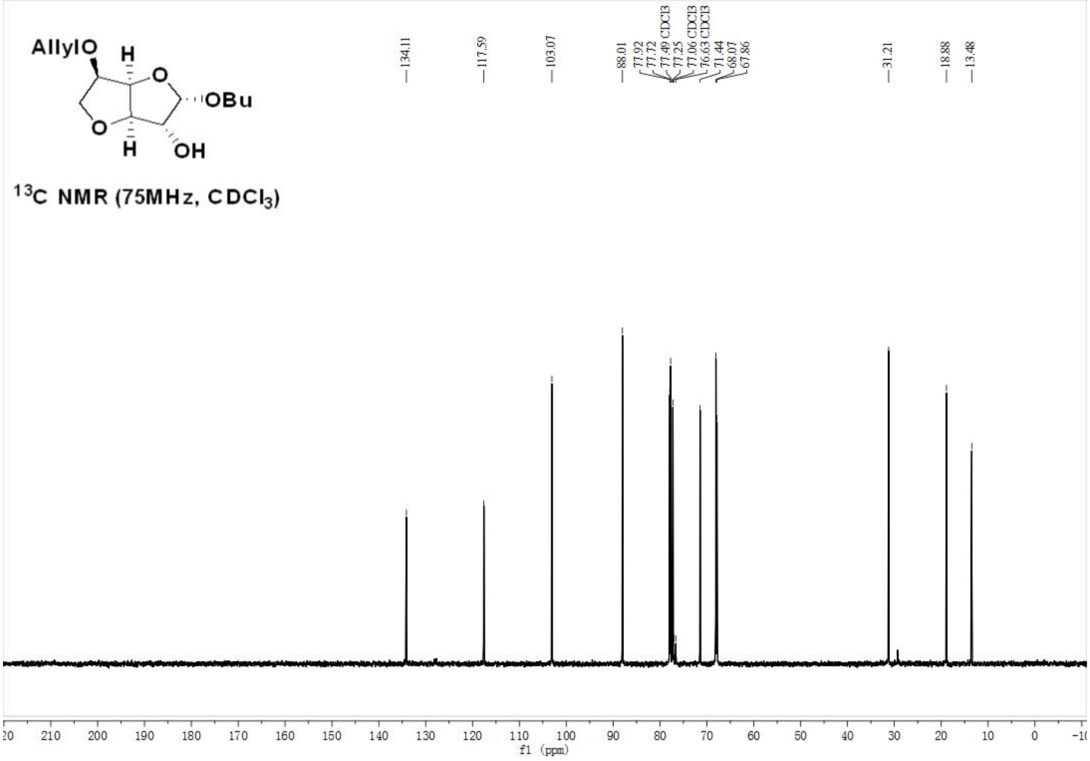
AllylO
H
O
OBu
O
H
OH
13C NMR (75MHz, CDCl3)
134.11
117.59
103.07
88.01
77.92
77.72
77.49 CDCl3
77.25
77.06 CDCl3
76.63 CDCl3
71.44
68.07
67.86
31.21
18.88
13.48
f1 (ppm)

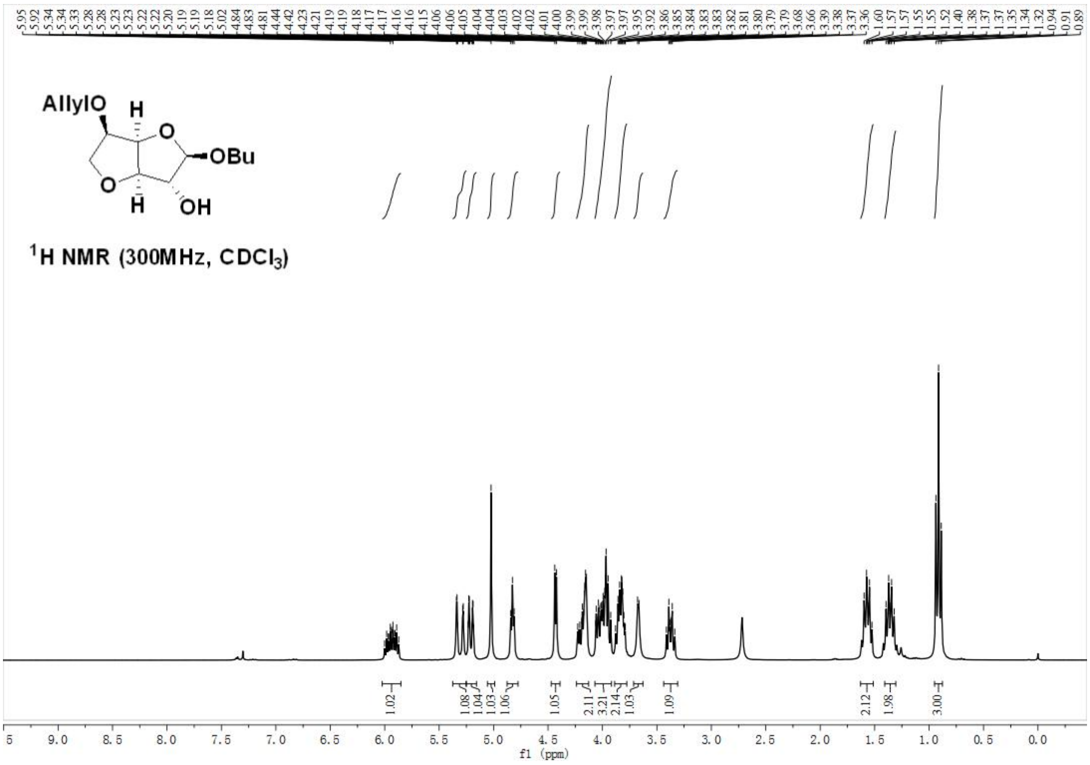
AllylO
H
O
OBu
O
H
OH
1H NMR (300MHz, CDCl3)
f1 (ppm)

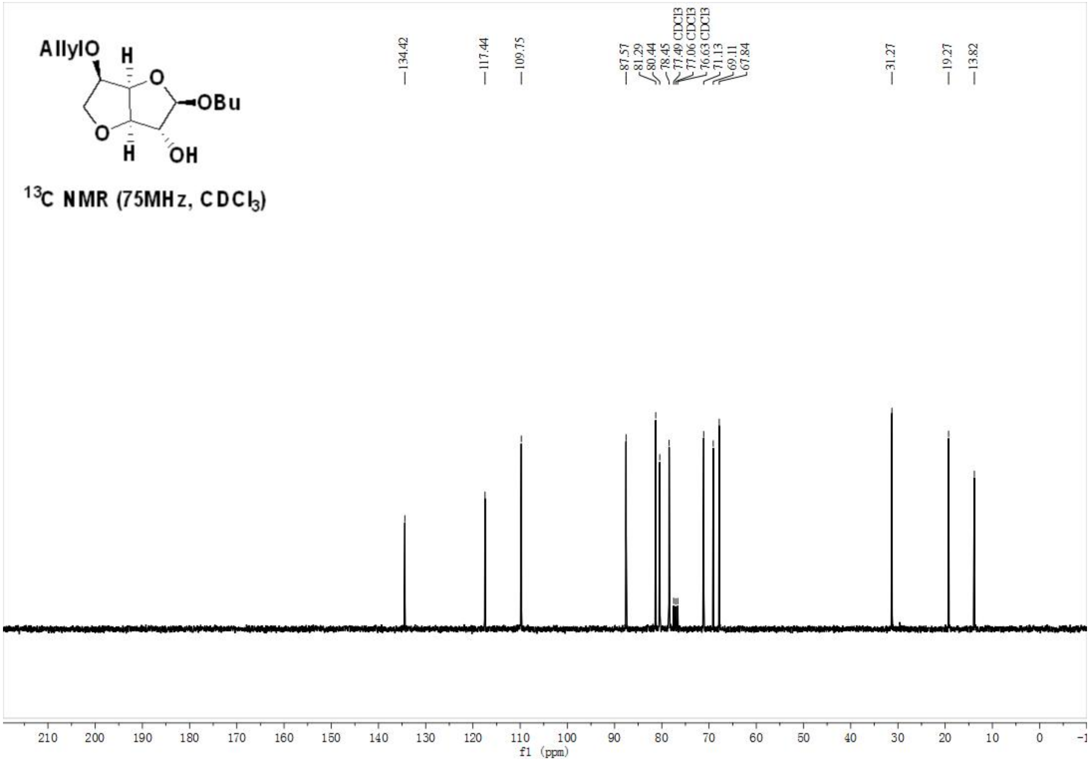
134.42
117.44
109.75
87.57
81.29
80.44
78.45
77.49 CDCl3
77.06 CDCl3
76.63 CDCl3
71.13
69.11
67.84
31.27
19.27
13.82
AllylO
H
O
OBu
O
H
OH
13C NMR (75MHz, CDCl3)
f1 (ppm)

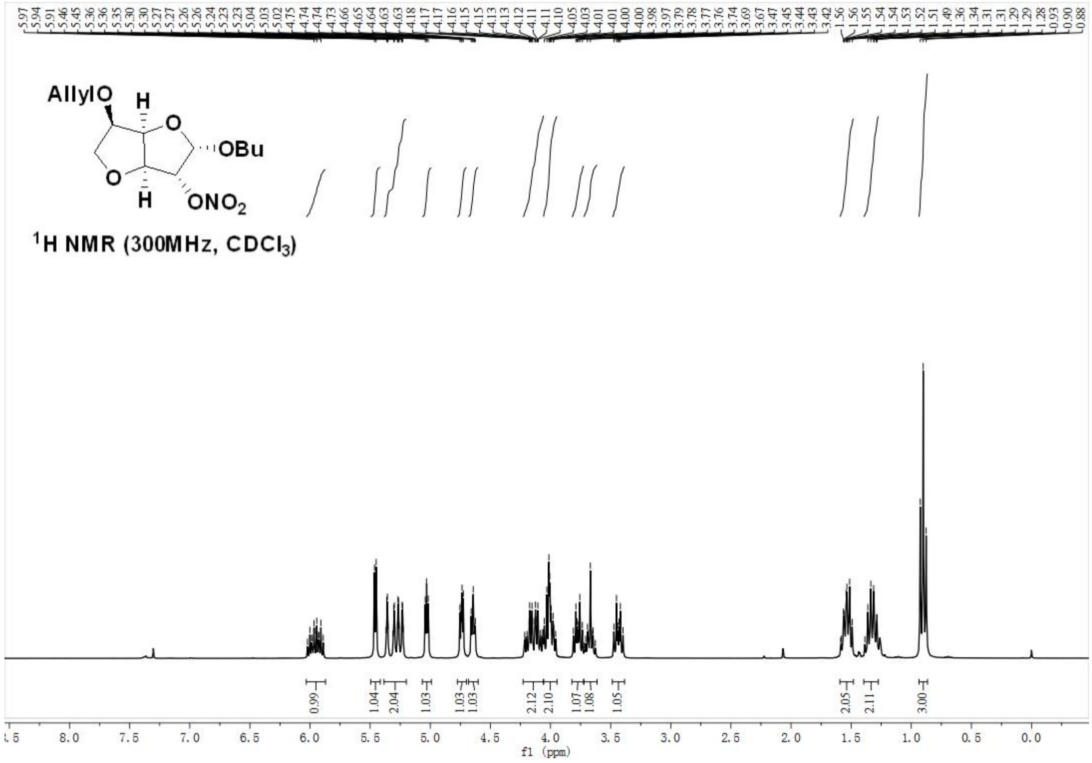
AllylO
H
O
OBu
O
H
ONO2
1H NMR (300MHz, CDCl3)
f1 (ppm)

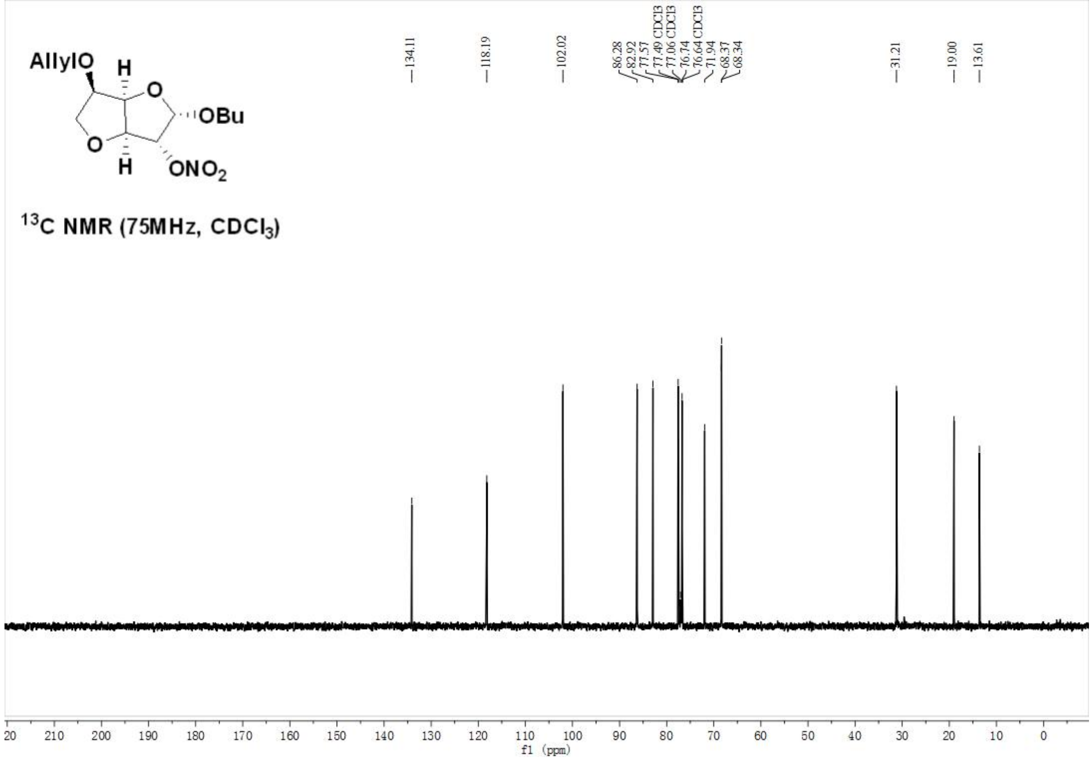
AllylO
H
O
OBu
O
H
ONO2
13C NMR (75MHz, CDCl3)
134.11
118.19
102.02
86.28
82.92
77.57
77.49 CDCl3
77.06 CDCl3
76.74
76.64 CDCl3
71.94
68.37
68.34
31.21
19.00
13.61
f1 (ppm)

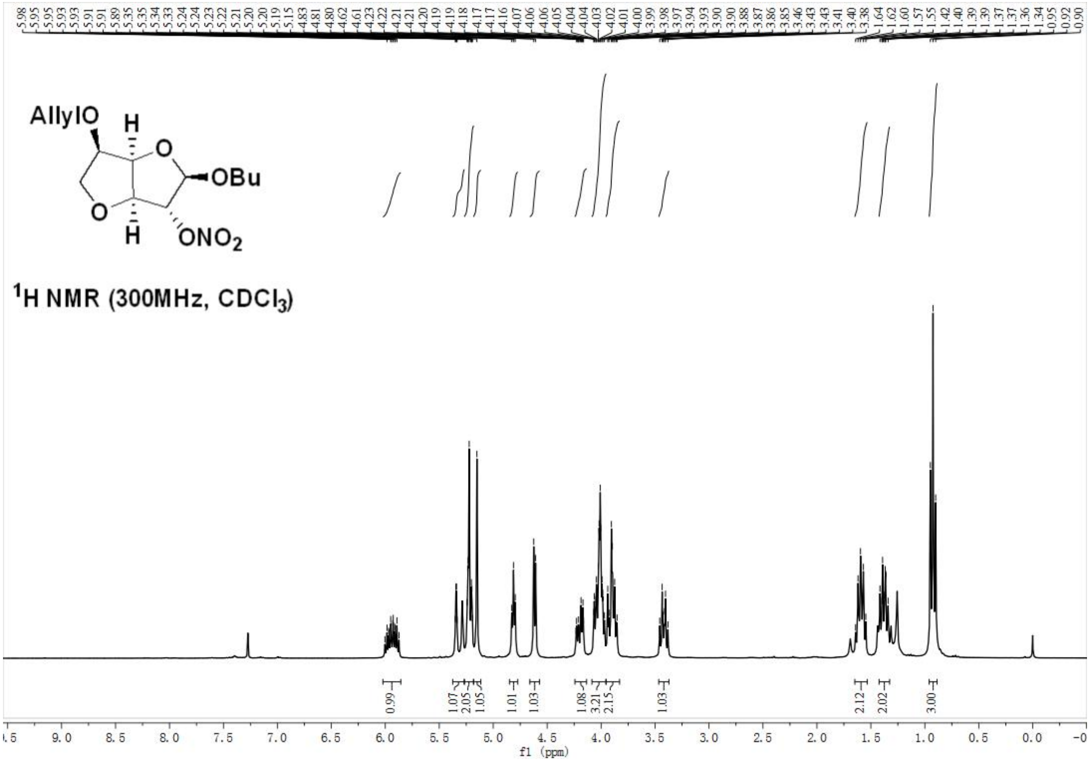
AllylO
H
O
OBu
O
H
ONO2
1H NMR (300MHz, CDCl3)
f1 (ppm)

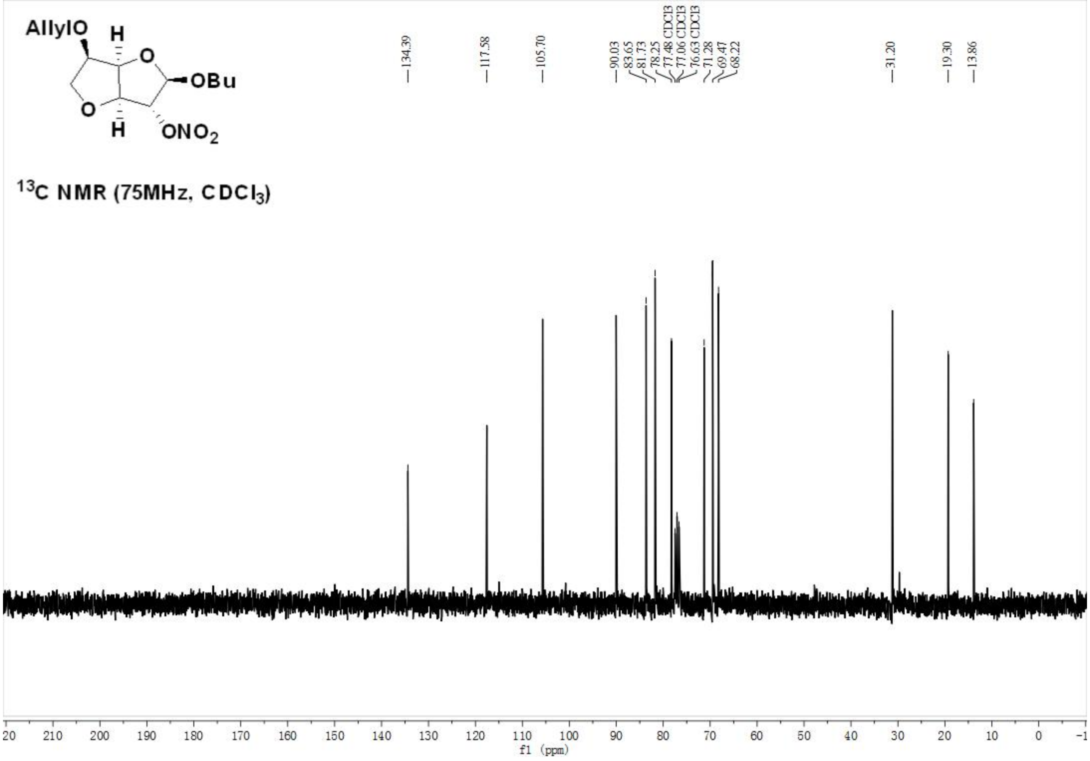
AllylO
H
O
OBu
O
H
ONO2
13C NMR (75MHz, CDCl3)
134.39
117.58
105.70
90.03
83.65
81.73
78.25
77.48 CDCl3
77.06 CDCl3
76.63 CDCl3
71.28
69.47
68.22
31.20
19.30
13.86
f1 (ppm)

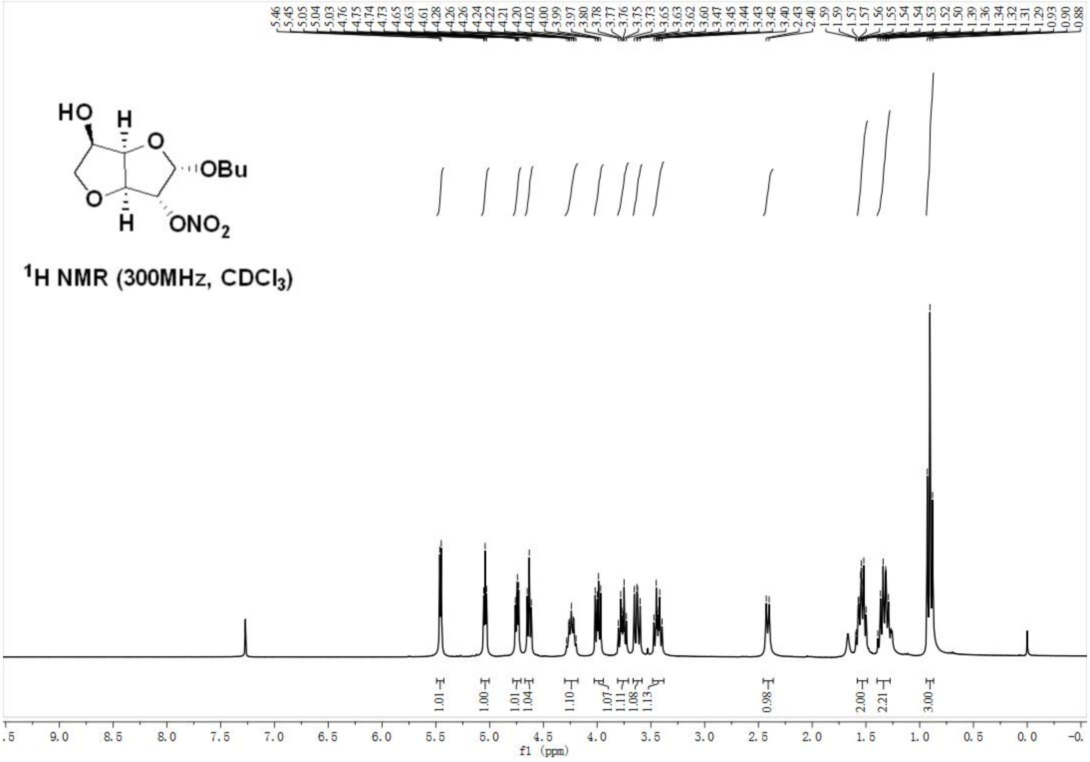
HO
H
O
OBu
O
H
ONO2
1H NMR (300MHz, CDCl3)
1.01
1.00
1.01
1.04
1.10
1.07
1.11
1.08
1.13
0.98
2.00
2.21
3.00
f1 (ppm)

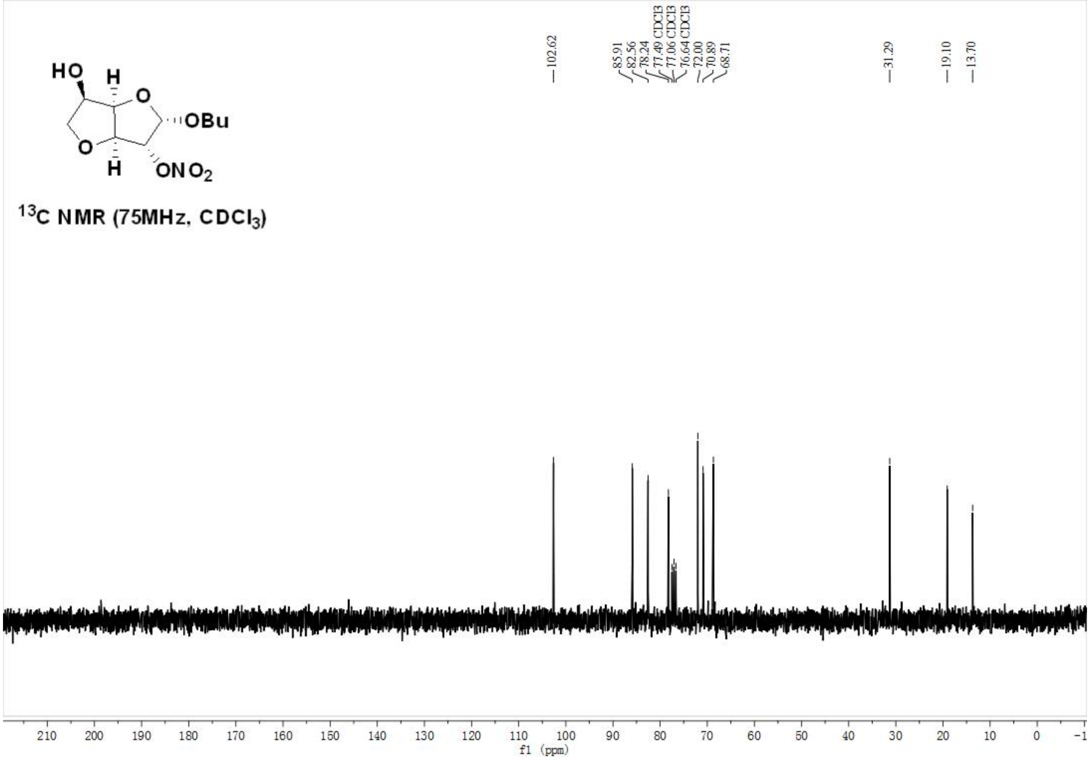
102.62
85.91
82.56
78.24
77.49 CDCl3
77.06 CDCl3
76.64 CDCl3
72.00
70.89
68.71
31.29
19.10
13.70
HO
H
O
OBu
O
H
ONO2
13C NMR (75MHz, CDCl3)
f1 (ppm)

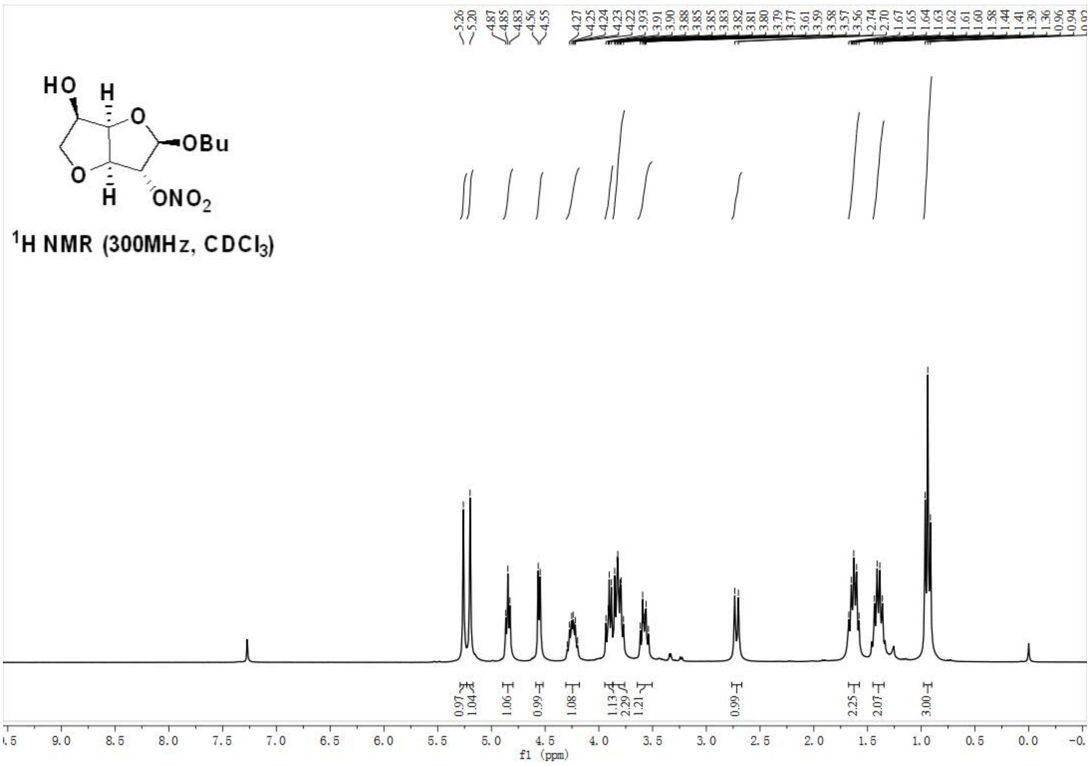
HO
H
O
OBu
O
H
ONO2
1H NMR (300MHz, CDCl3)
f1 (ppm)

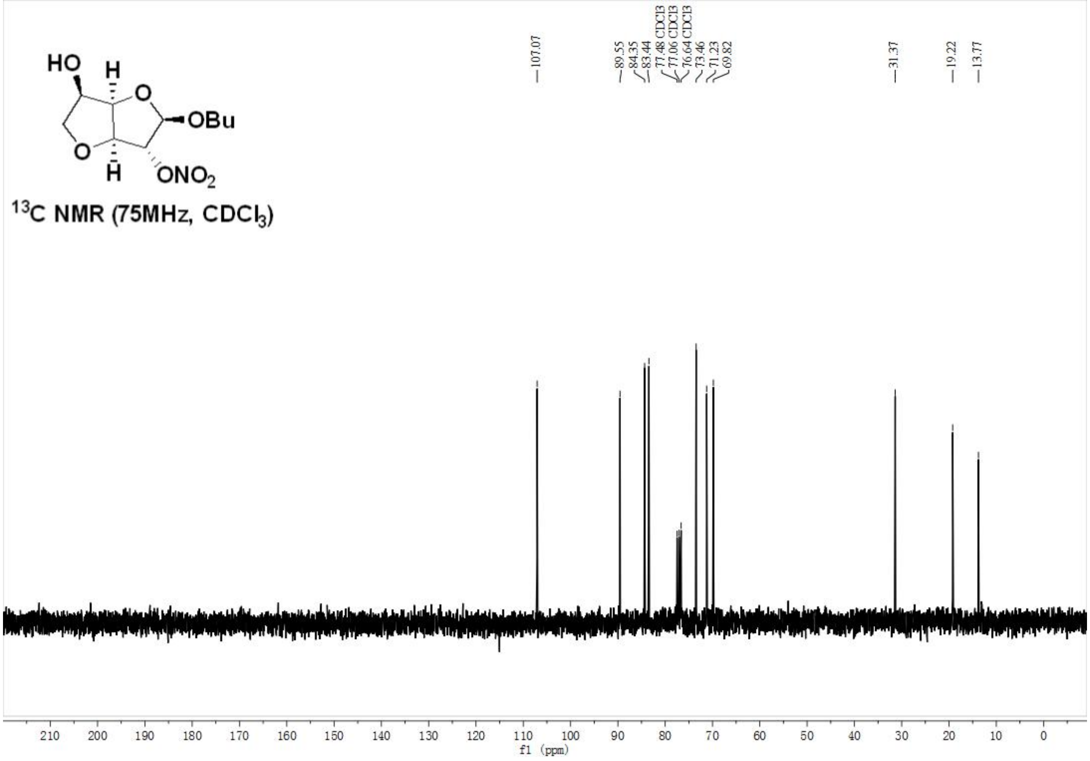
HO
H
O
OBu
O
H
ONO2
13C NMR (75MHz, CDCl3)
f1 (ppm)

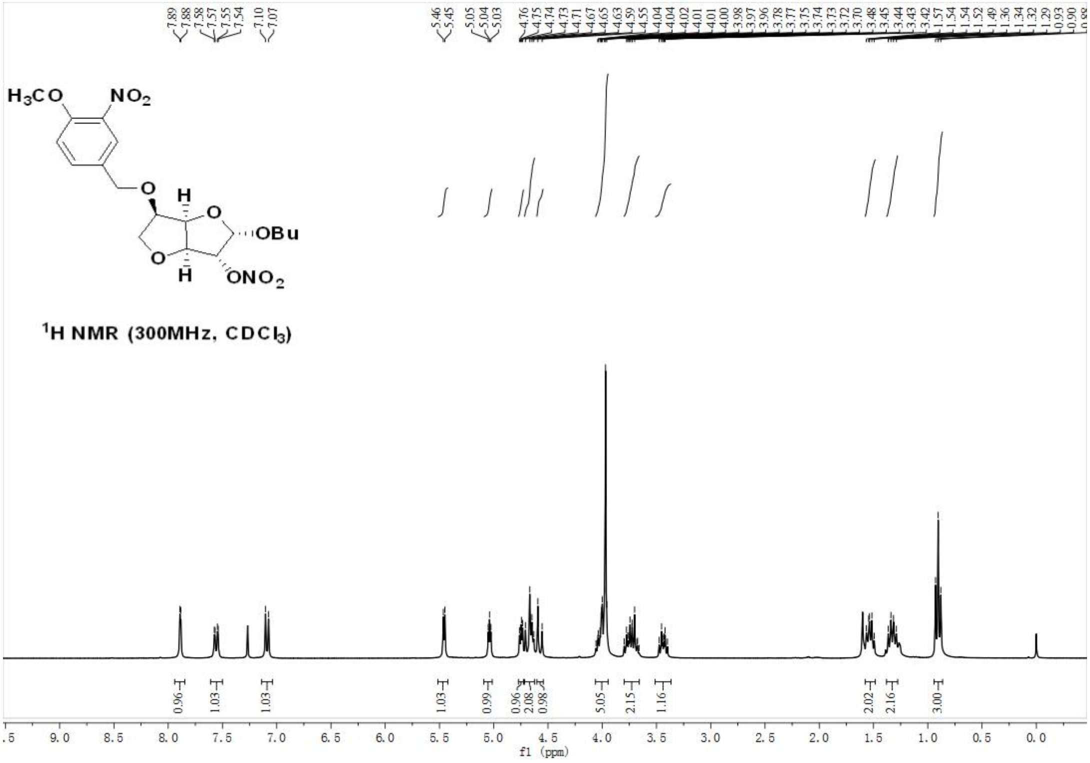

H3CO
NO2
OBu
ONO2
H
H
O
1H NMR (300MHz, CDCl3)
f1 (ppm)

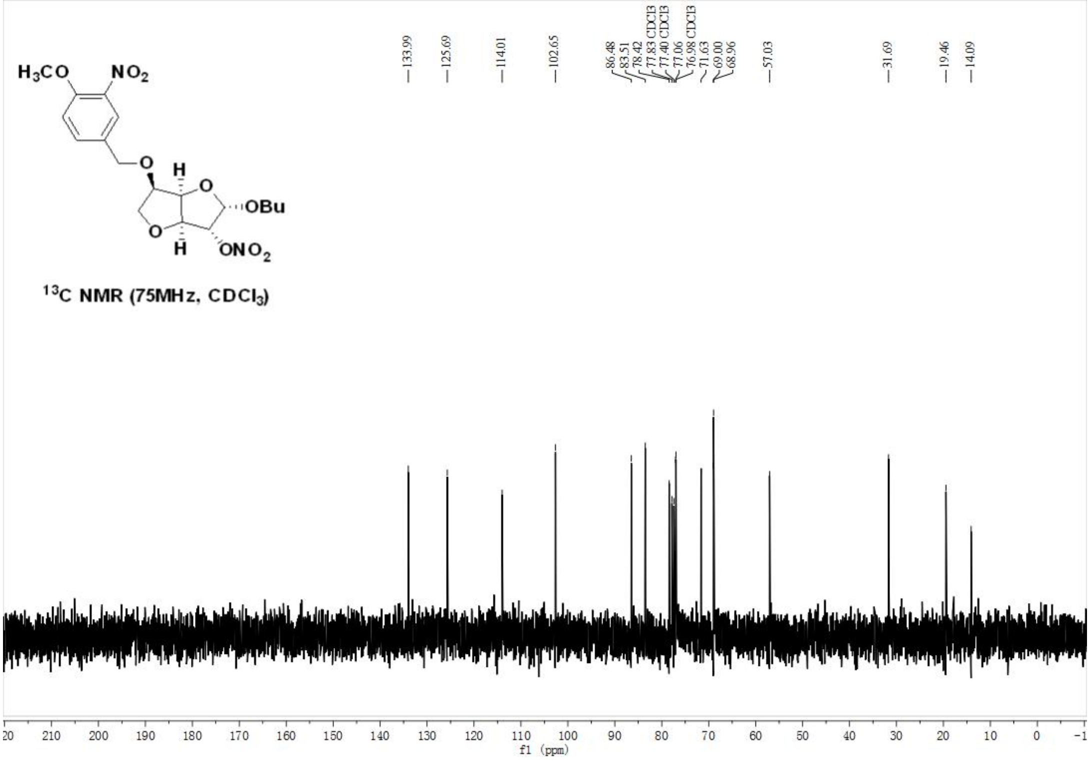

H3CO
NO2
OBu
ONO2
H
H
O
13C NMR (75MHz, CDCl3)
133.99
125.69
114.01
102.65
86.48
83.51
78.42
77.83 CDCl3
77.40 CDCl3
77.06
76.98 CDCl3
71.63
69.00
68.96
57.03
31.69
19.46
14.09
f1 (ppm)

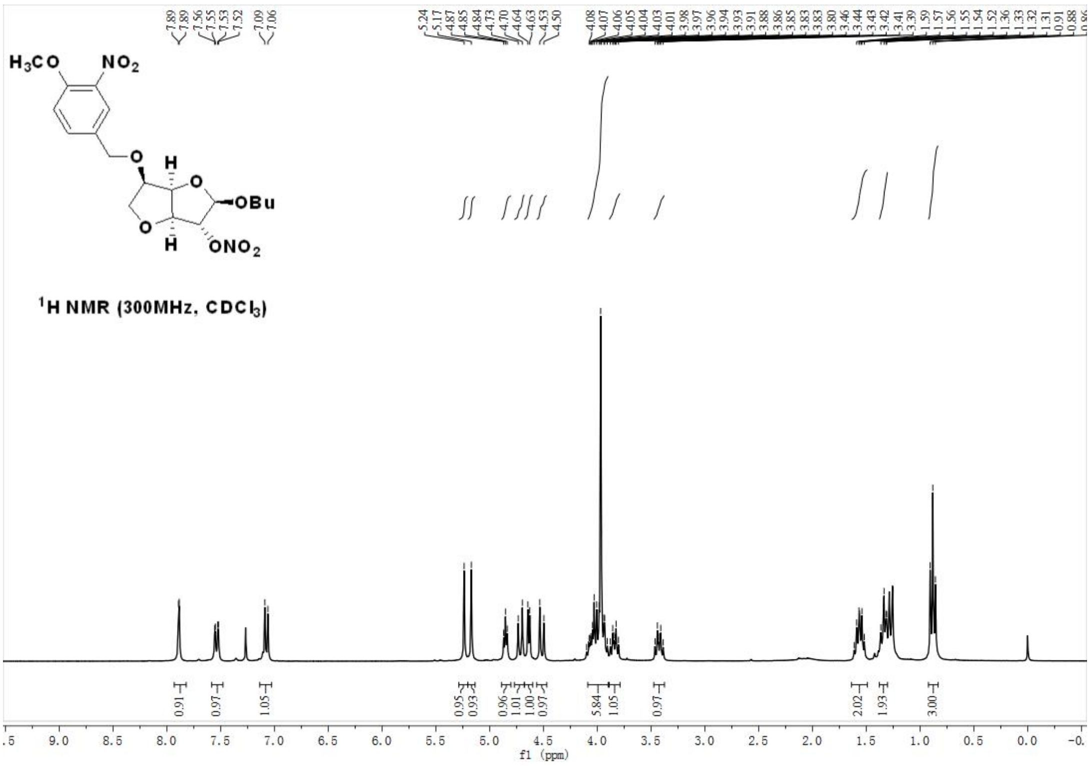
H3CO
NO2
H
O
OBu
O
H
ONO2
1H NMR (300MHz, CDCl3)
0.91
0.97
1.05
0.95
0.93
0.96
1.01
1.00
0.97
5.84
1.05
0.97
2.02
1.93
3.00
f1 (ppm)

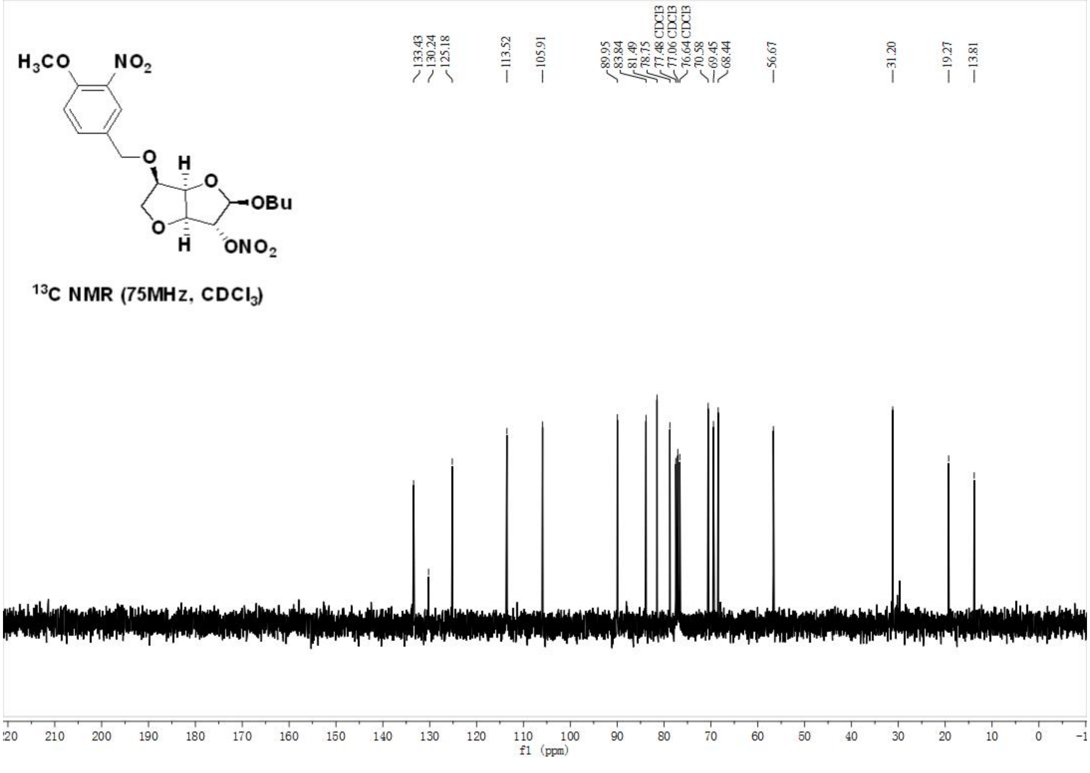
H3CO
NO2
H
O
OBu
O
H
ONO2
13C NMR (75MHz, CDCl3)
133.43
130.24
125.18
113.52
105.91
89.95
83.84
81.49
78.75
77.48 CDCl3
77.06 CDCl3
76.64 CDCl3
70.58
69.45
68.44
56.67
31.20
19.27
13.81
f1 (ppm)

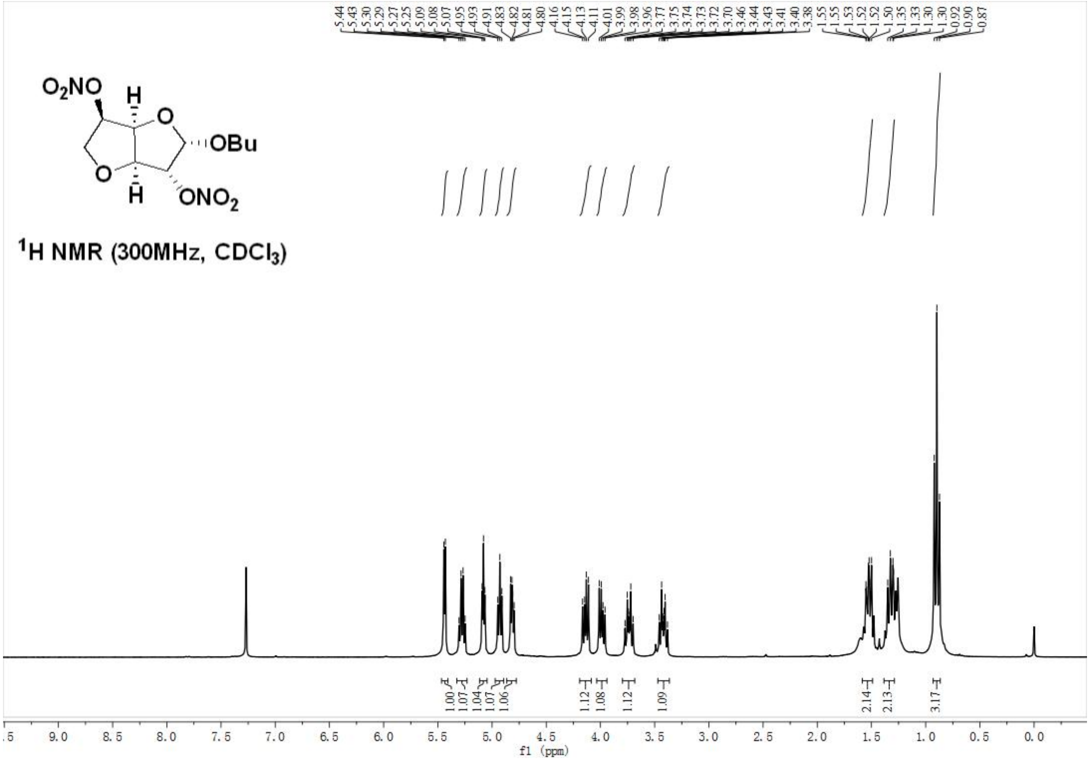
1H NMR (300MHz, CDCl3)

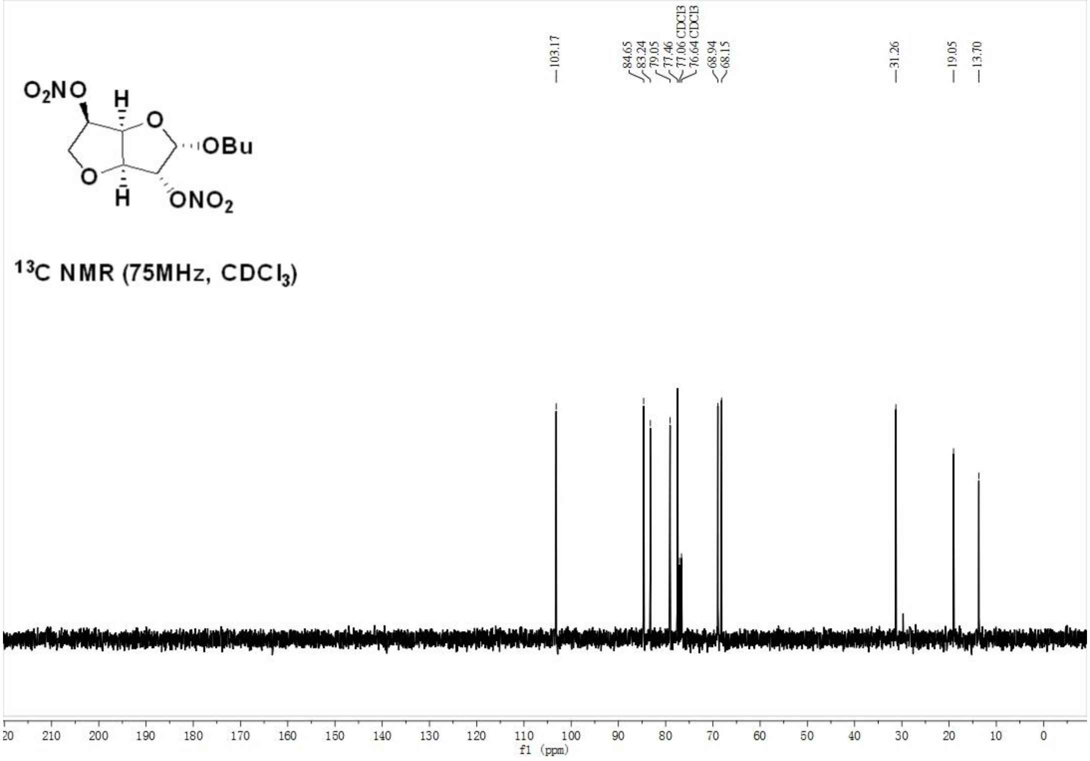
13C NMR (75MHz, CDCl3)

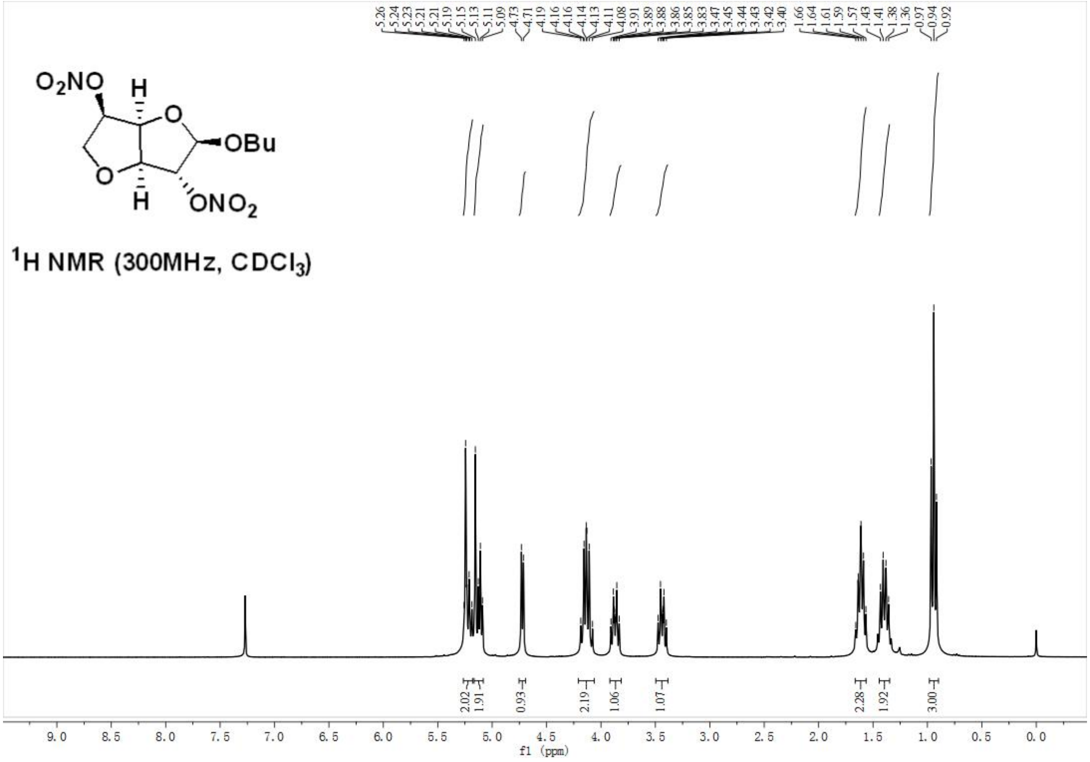

O2NO
H
O
OBu
O
H
ONO2
1H NMR (300MHz, CDCl3)

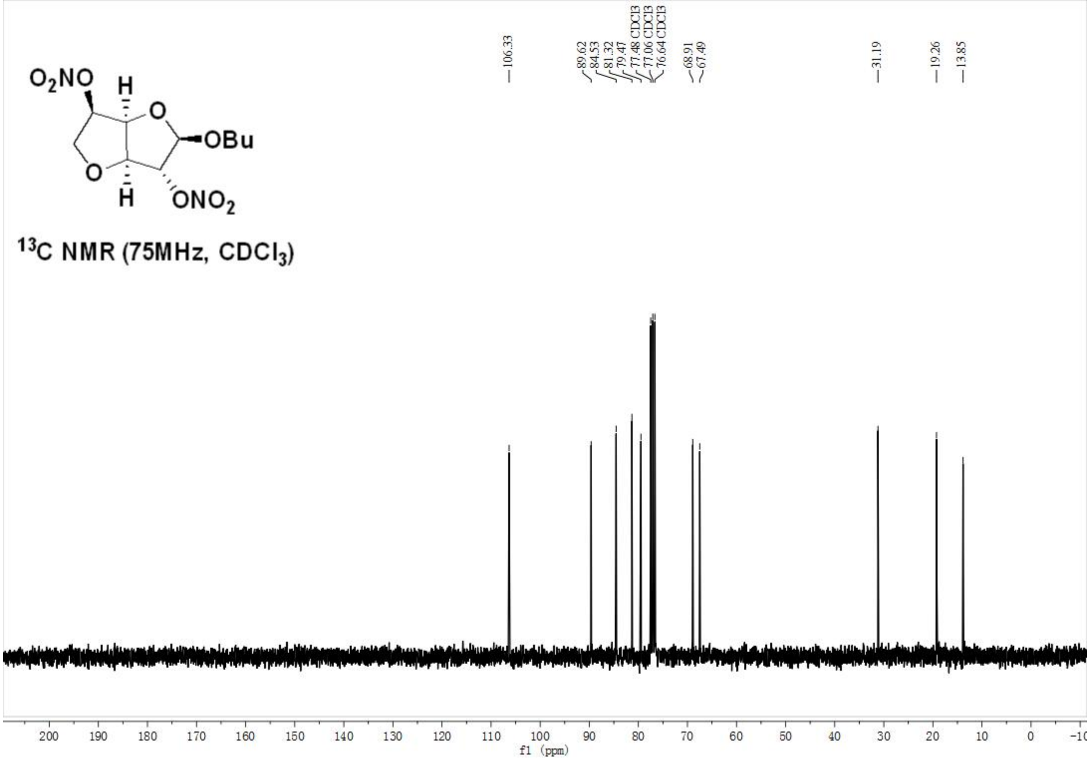

O2NO
H
O
OBu
O
H
ONO2
13C NMR (75MHz, CDCl3)

2. Copies of IR

Copies of IR of **5MNS-1**

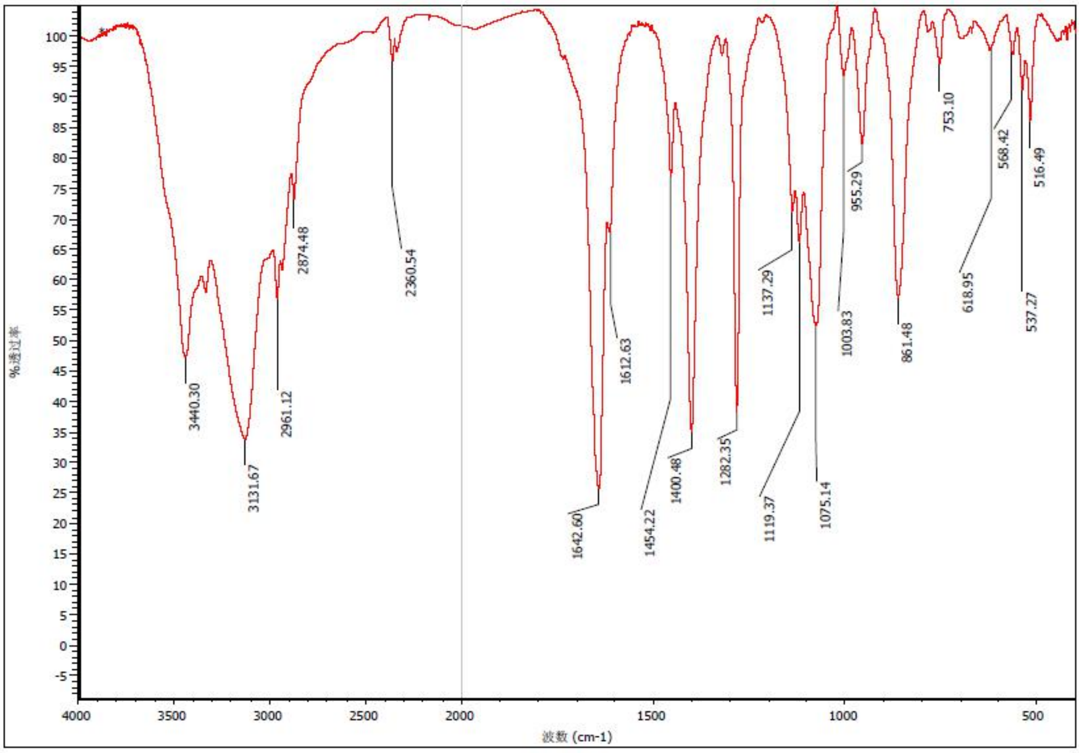

Copies of IR of **5MNS-2**

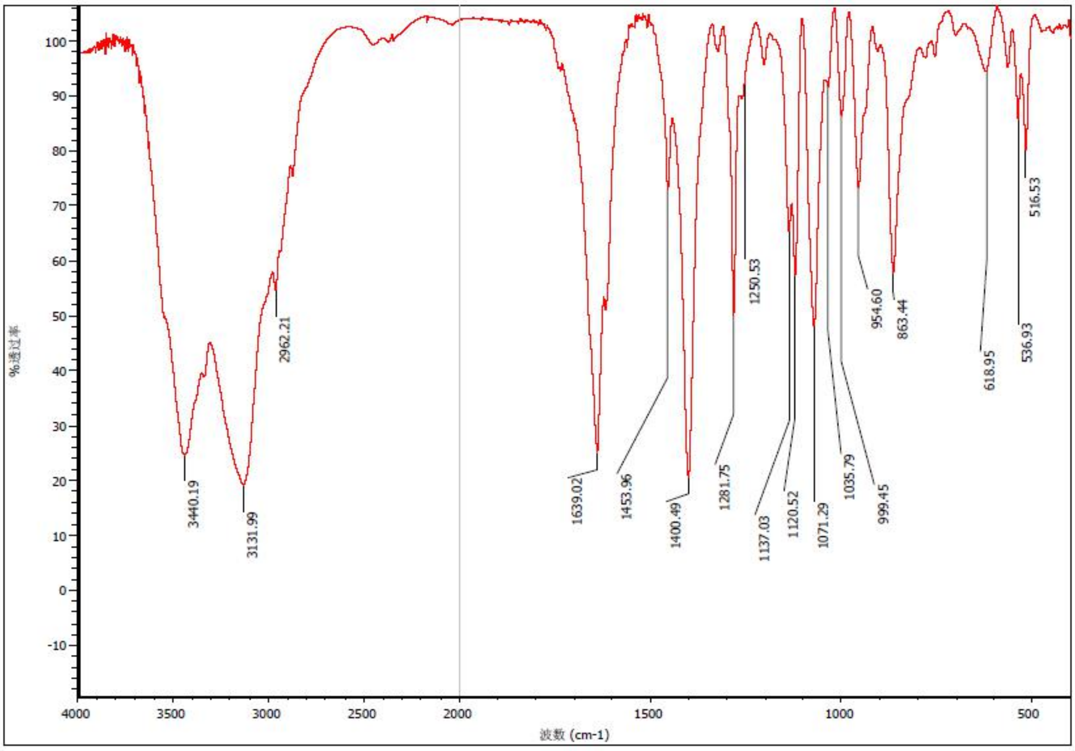

Copies of IR of **5MNS-3**

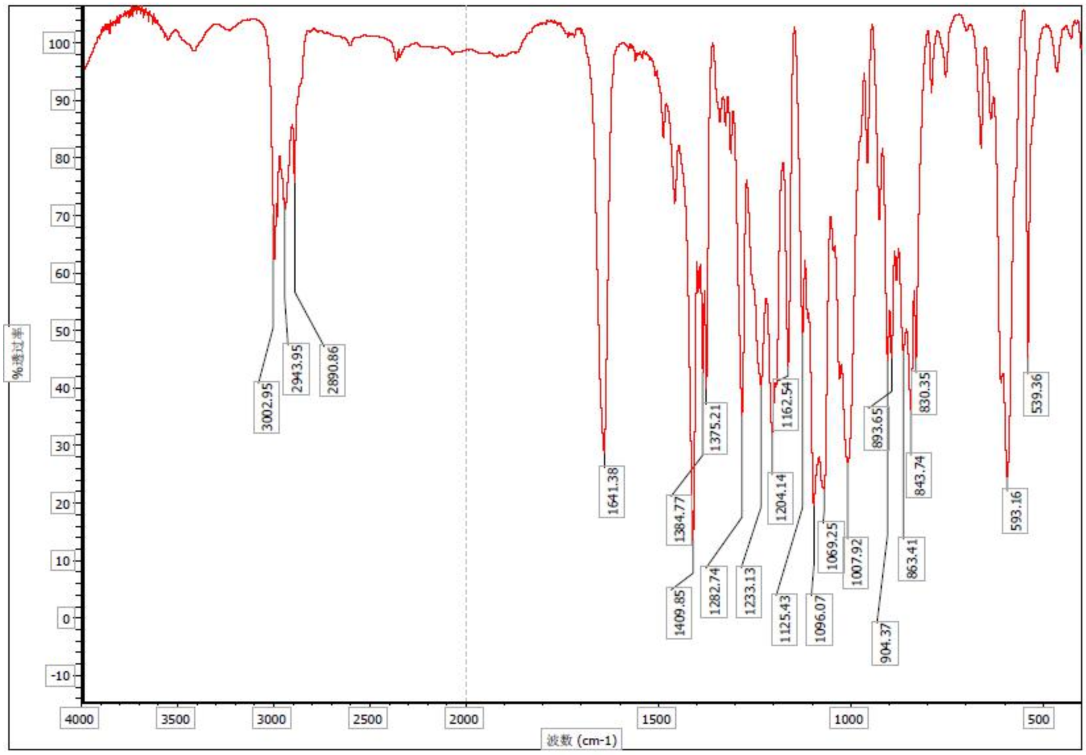

Copies of IR of **5MNS-4**

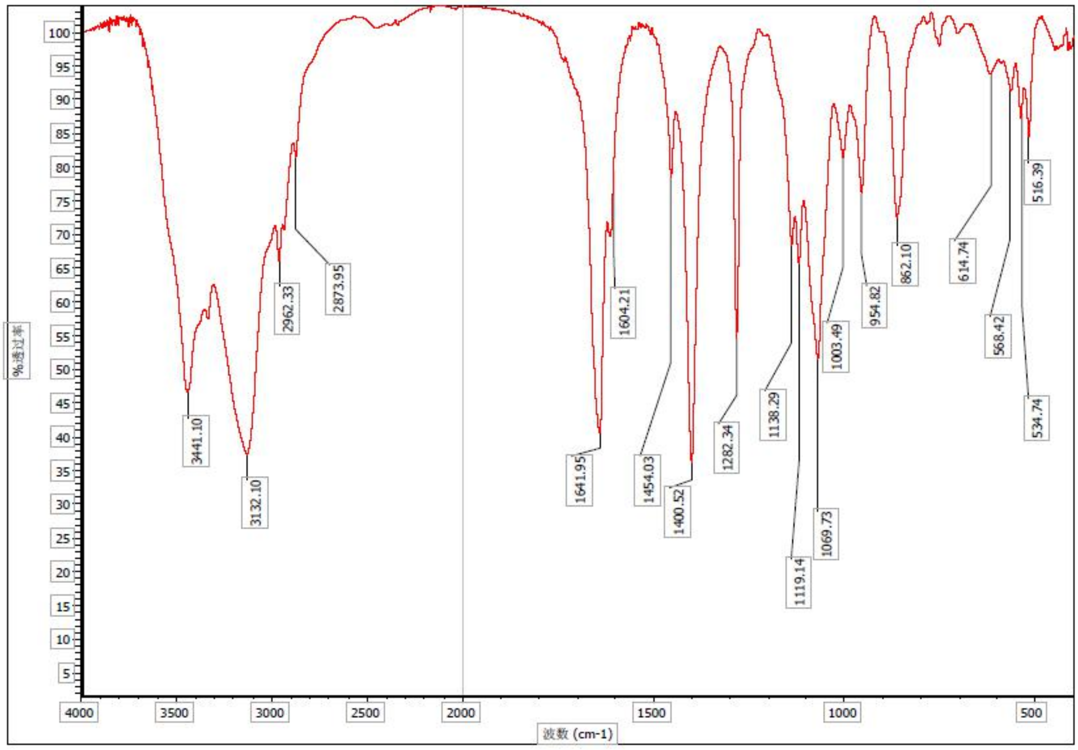

Copies of IR of **5MNS-5**

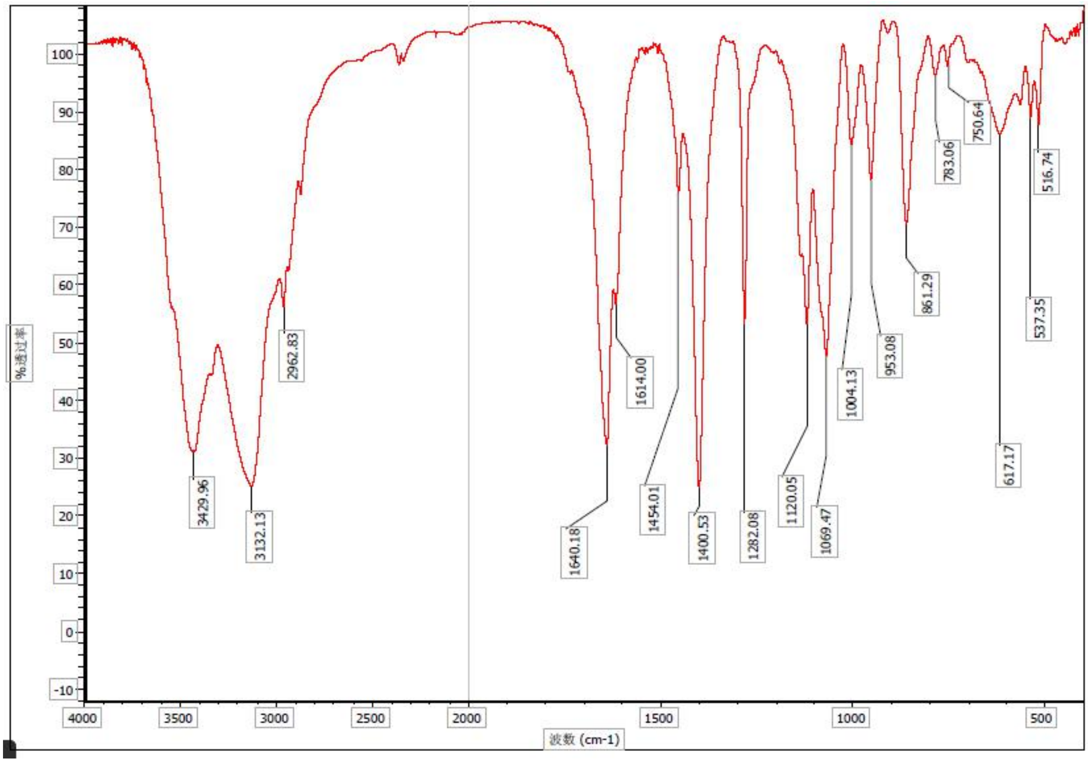

Copies of IR of **2MNS-1**

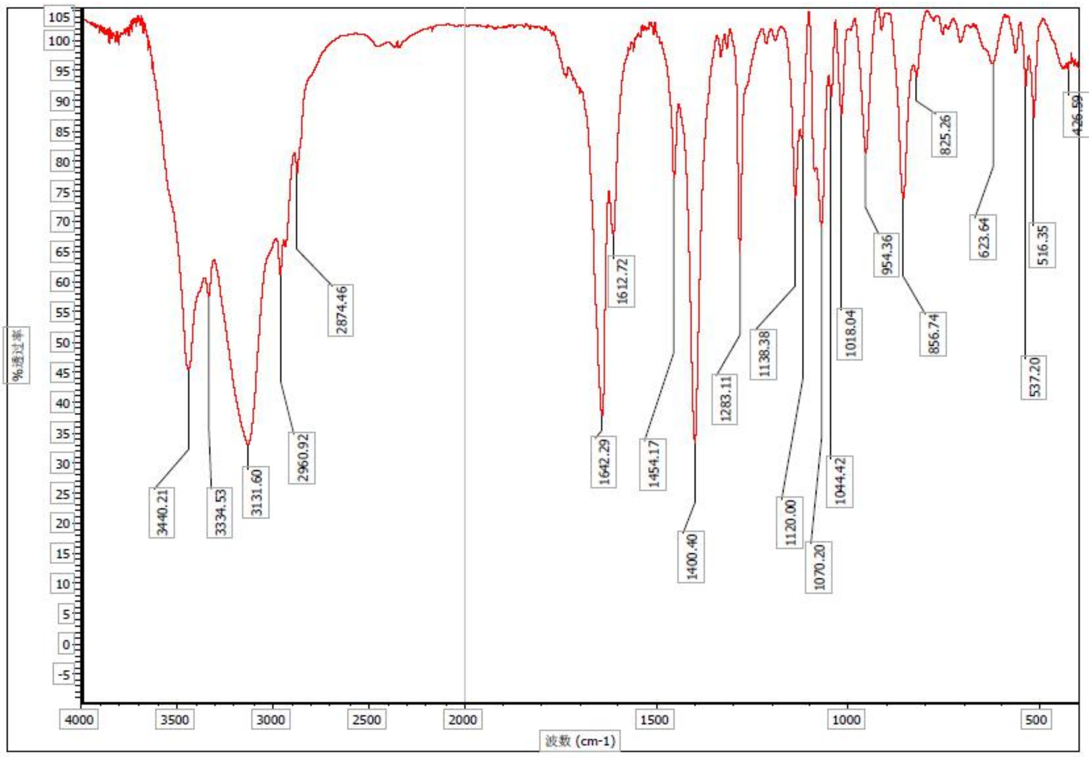

Copies of IR of **2MNS-2**

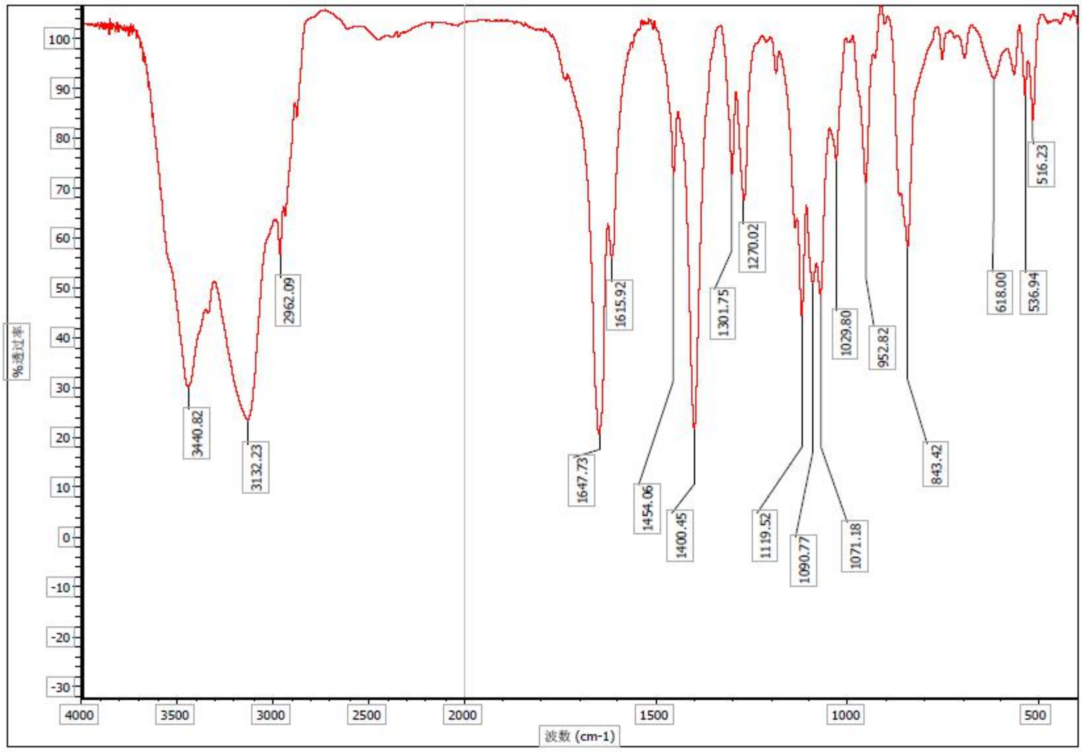

Copies of IR of **8**

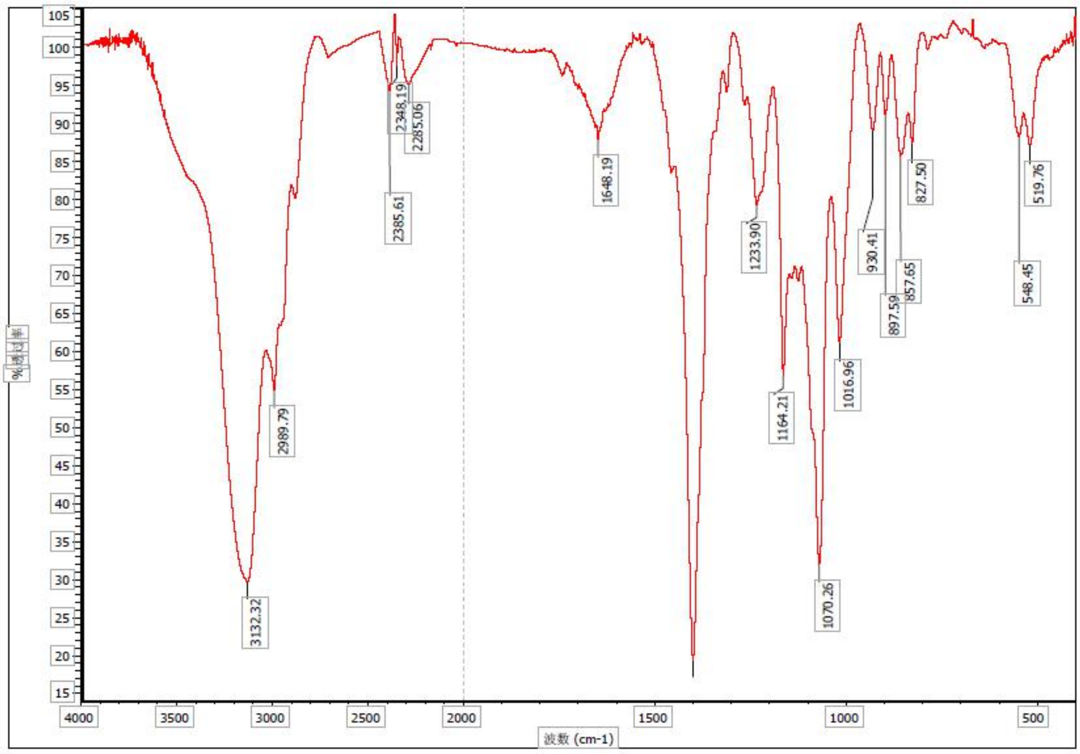

Copies of IR of **9a**

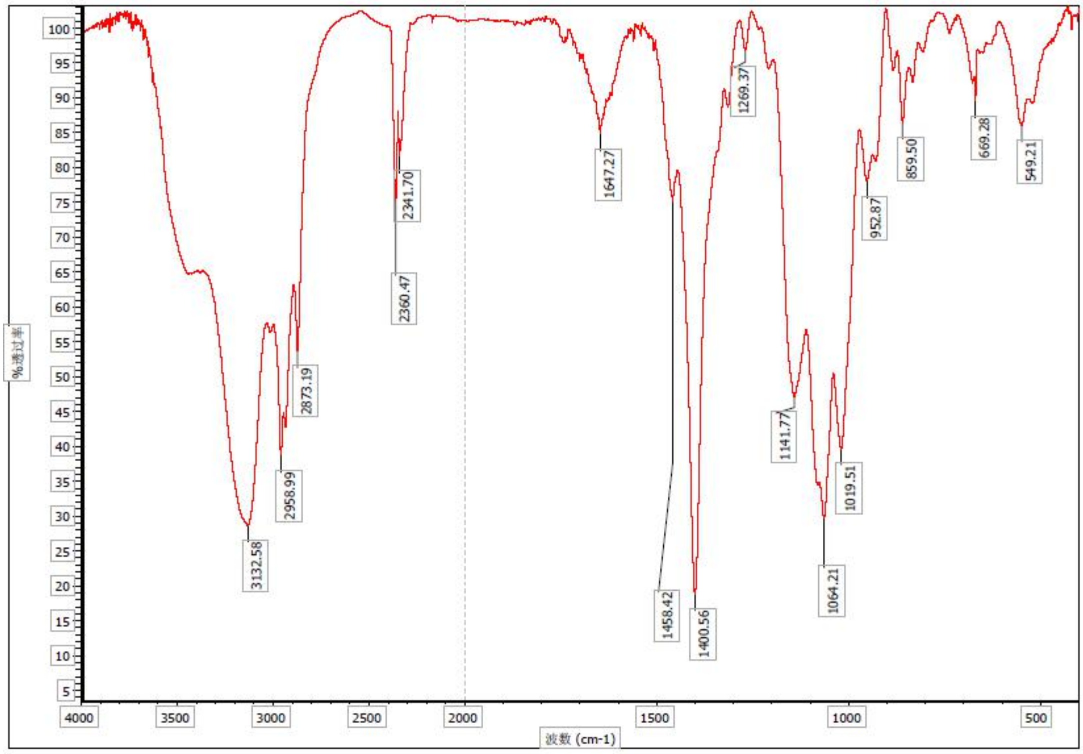

Copies of IR of **9b**

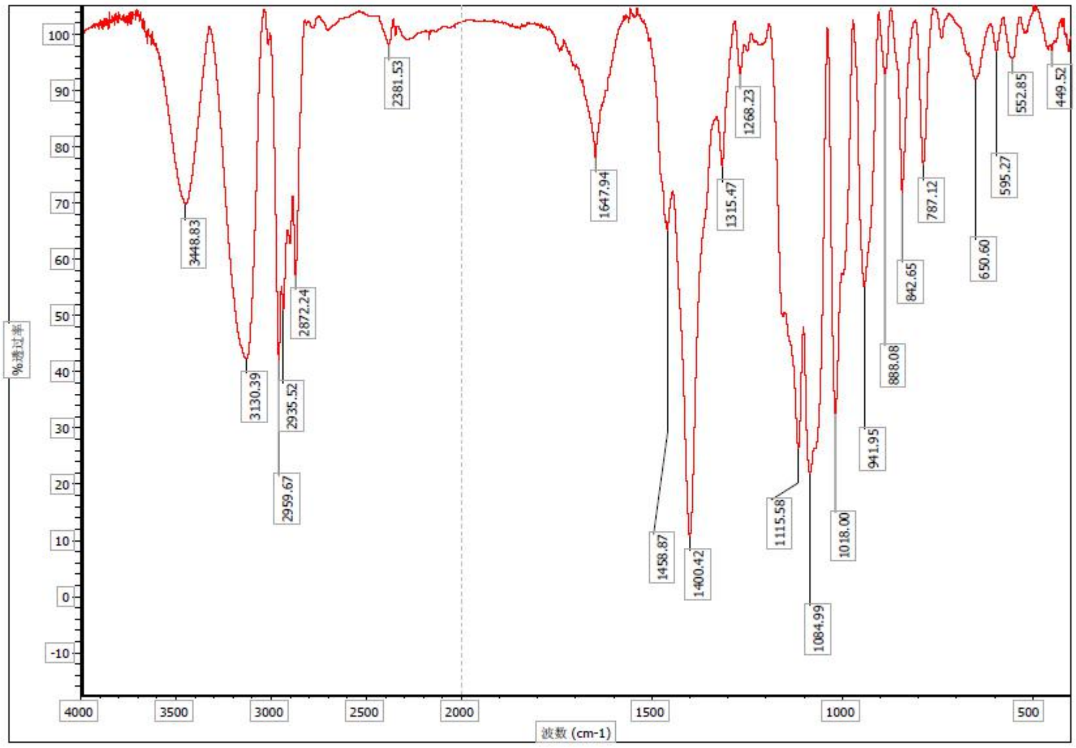

Copies of IR of **2MNS-3**

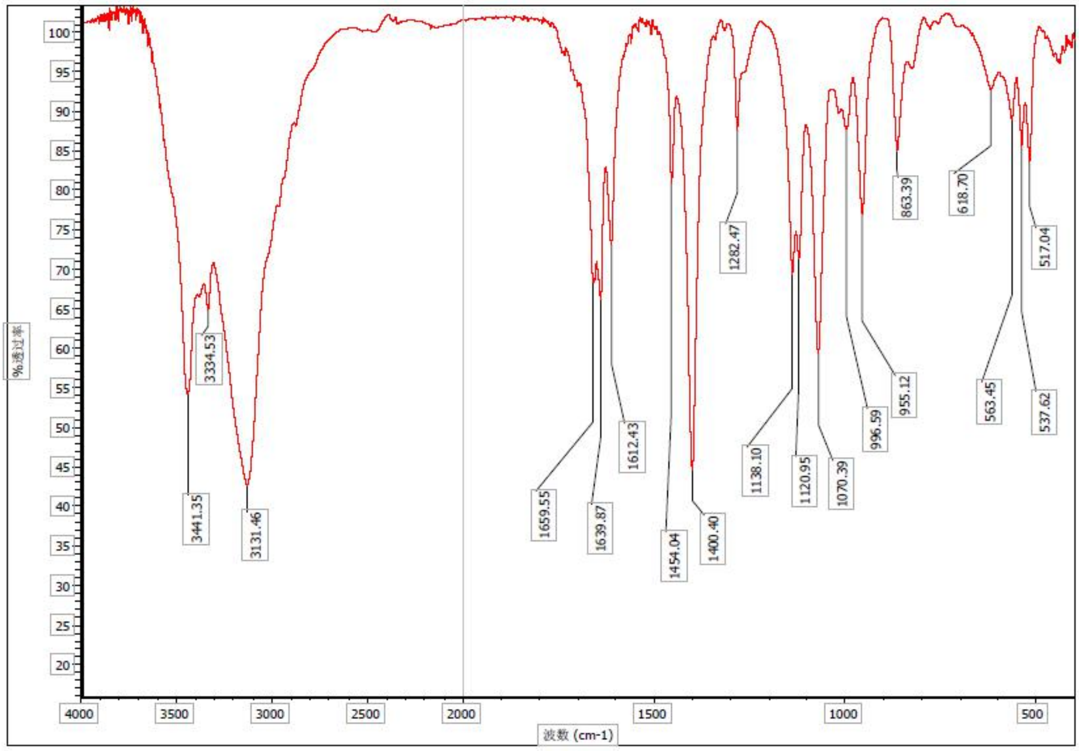

Copies of IR of **2MNS-4**

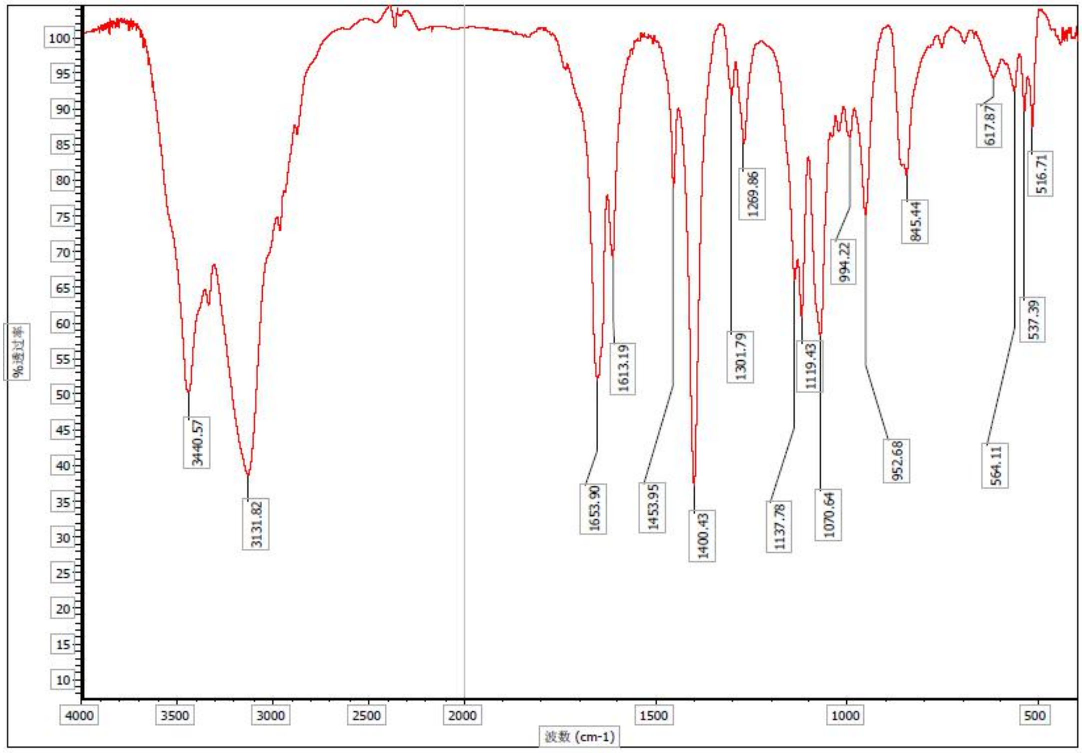

Copies of IR of **2MNS-5**

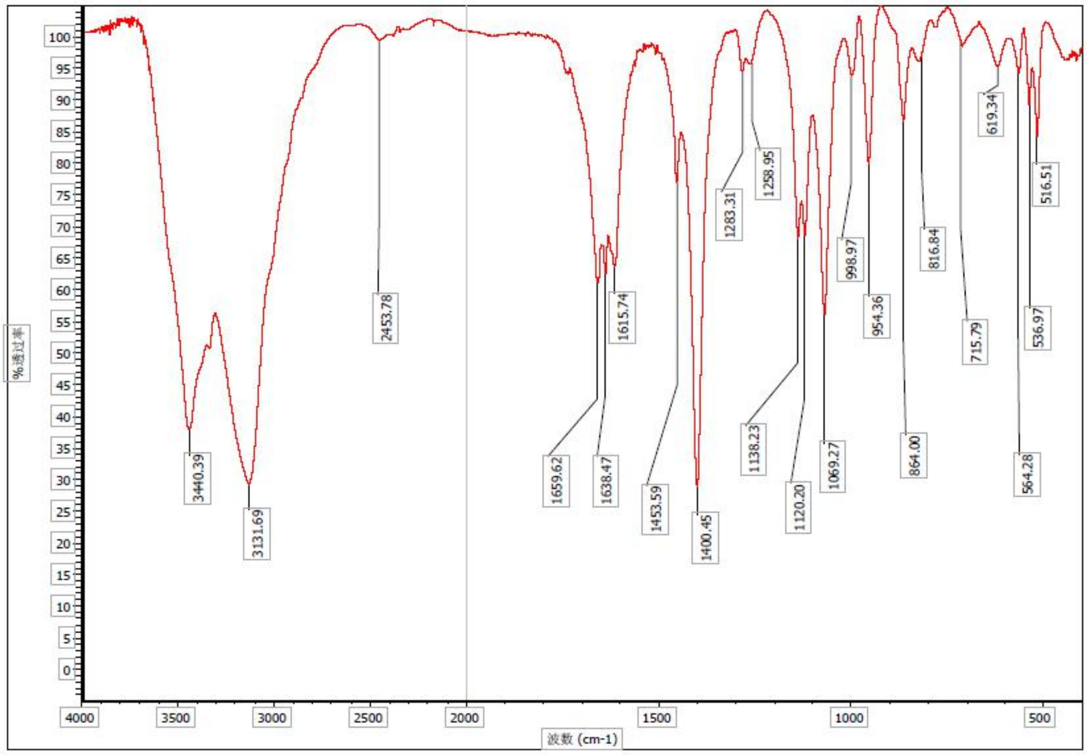

Copies of IR of **2MNS-6**

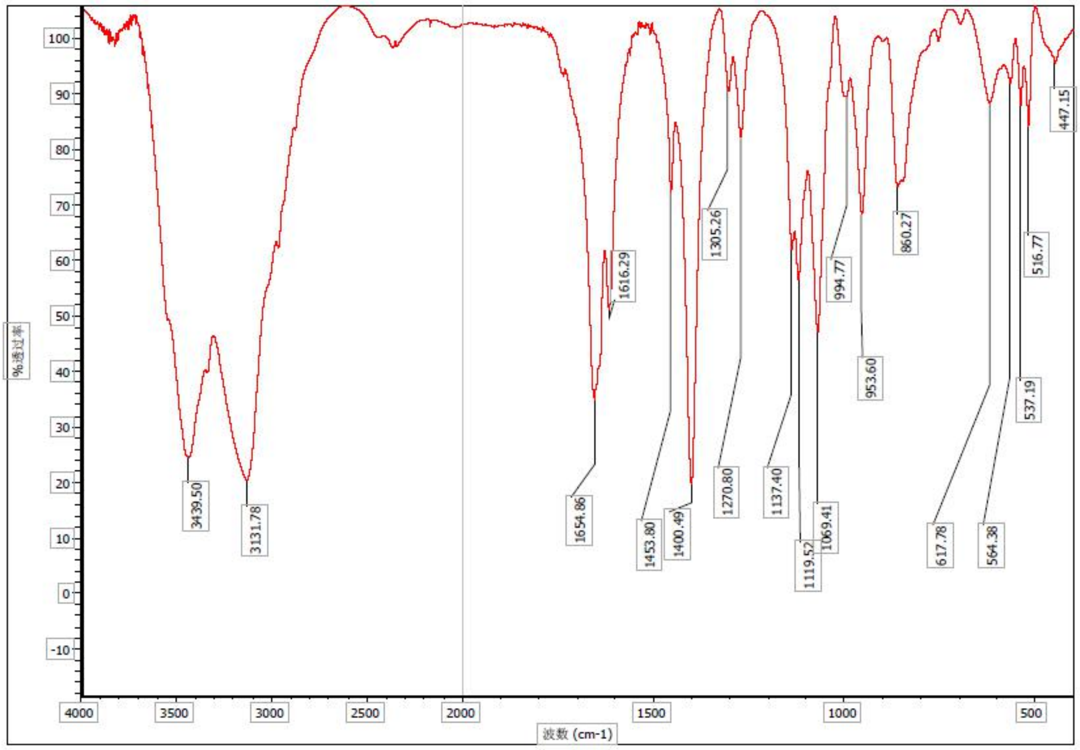

Copies of IR of **2MNS-7**

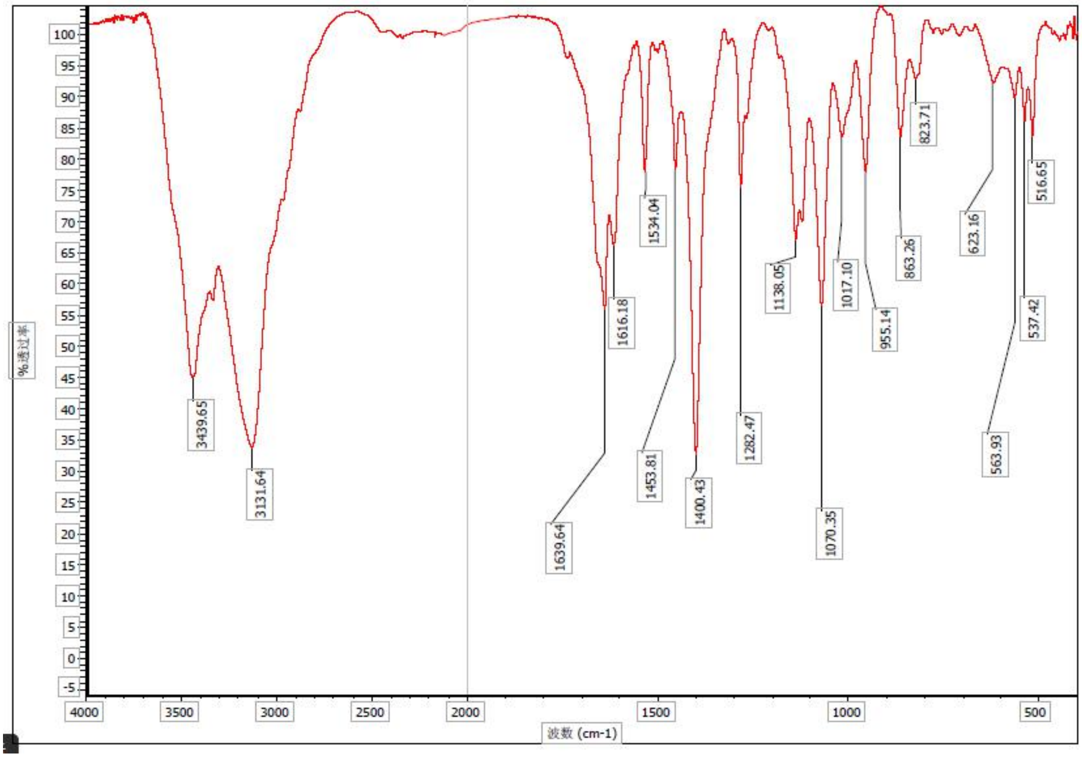

Copies of IR of **2MNS**-8

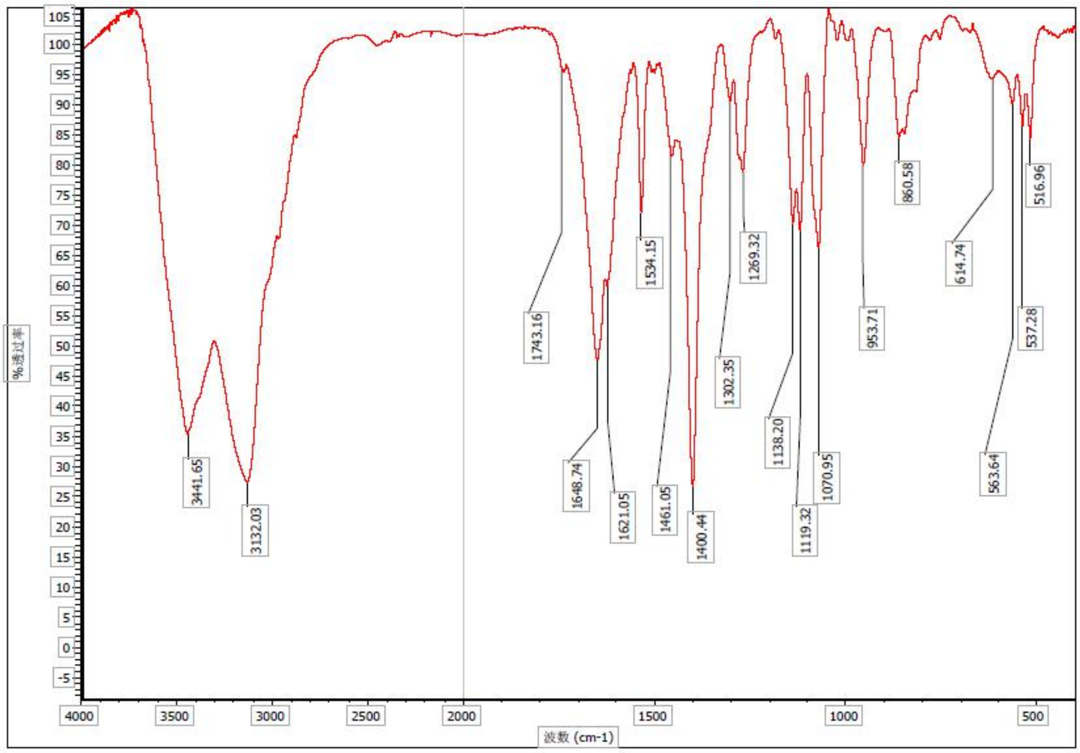

Copies of IR of **DNS-1**

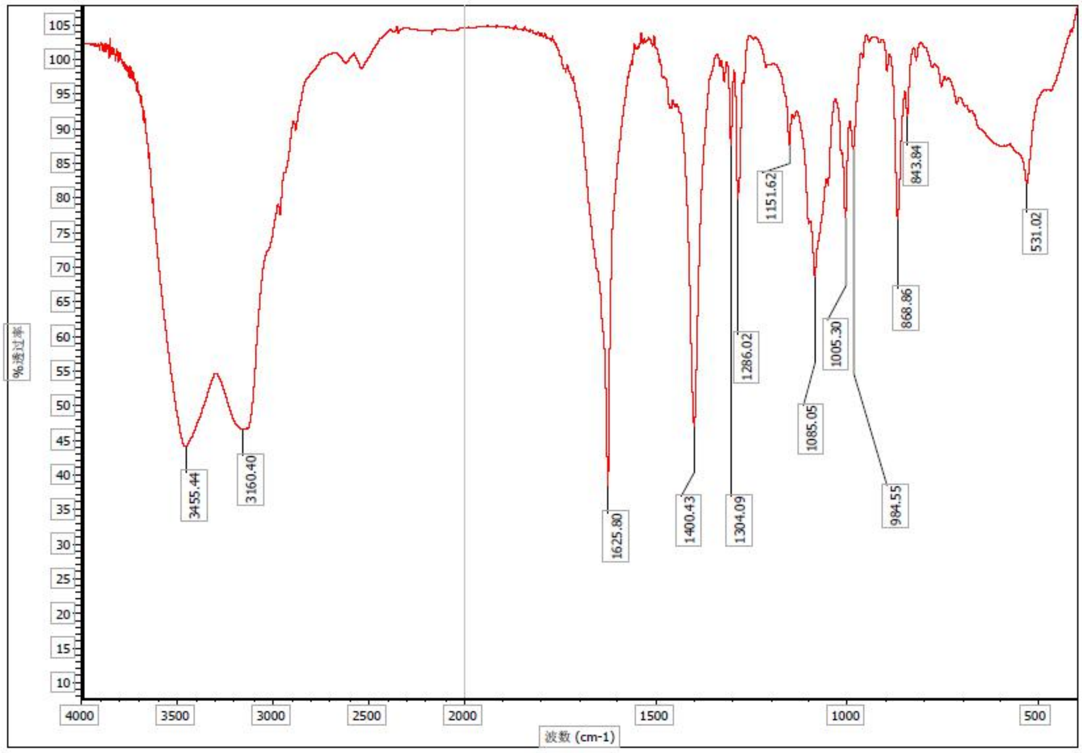

Copies of IR of **DNS-2**

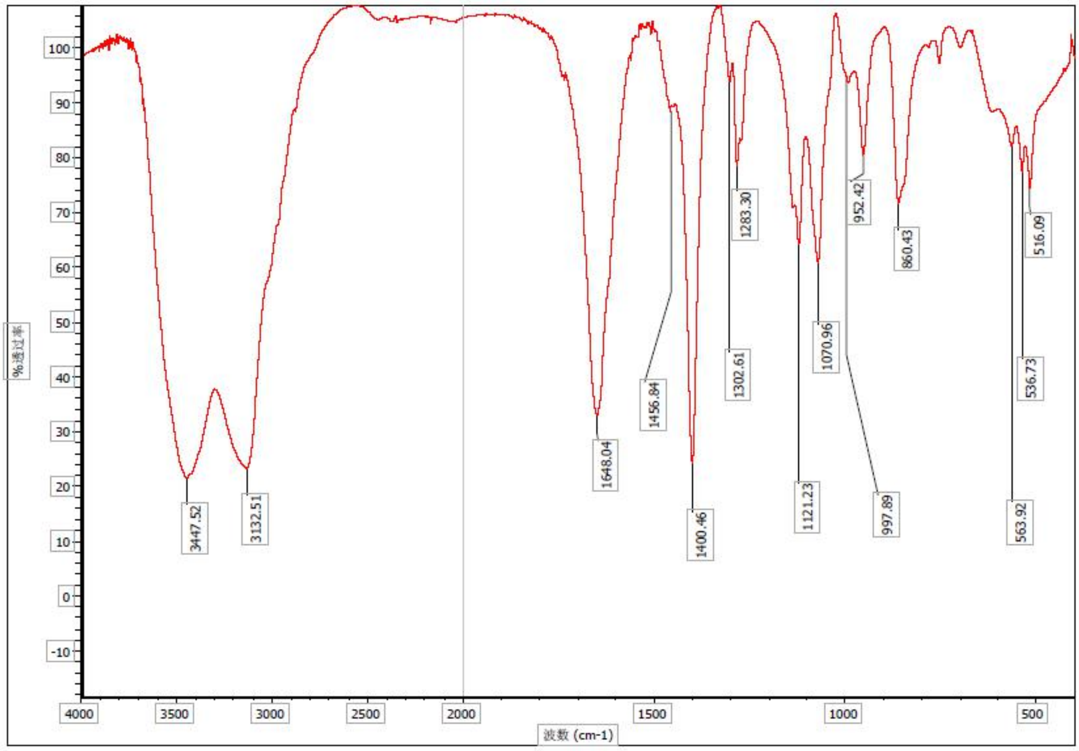

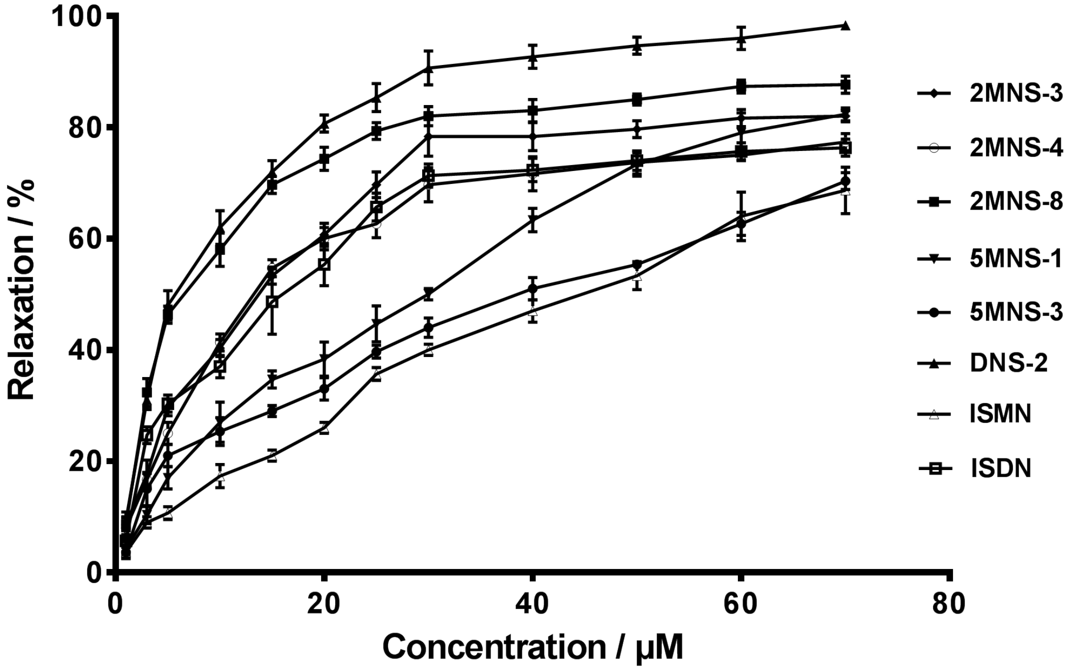

Figure.1S. The inhibitory effect of nitrates derivatives on the contraction induced by phenylephrine in mesenteric artery rings.

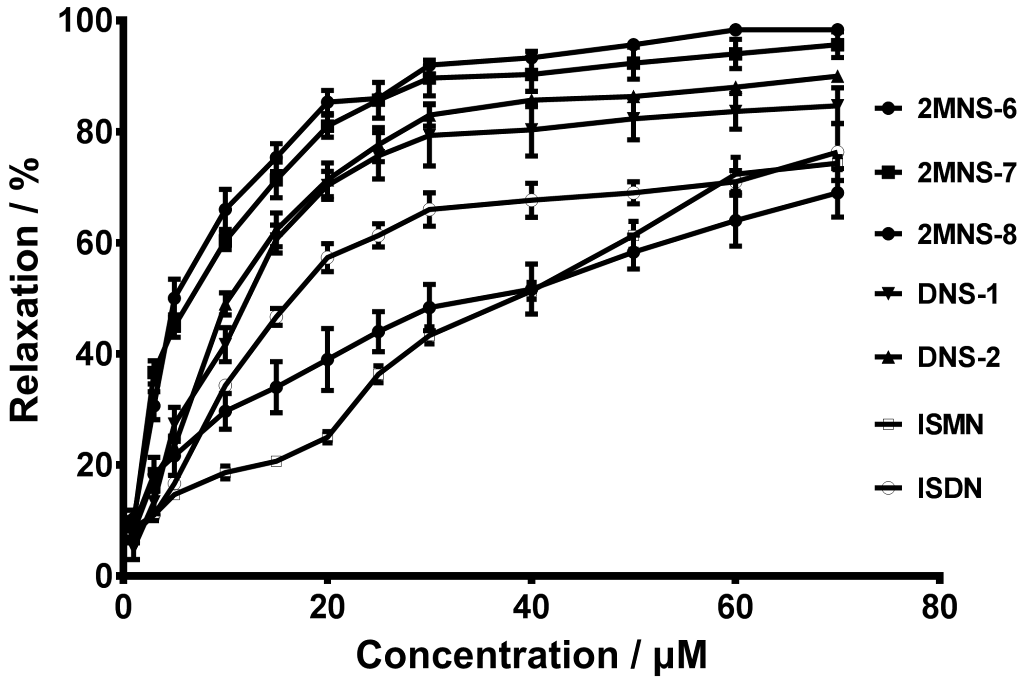

Figure.2S. The inhibitory effect of nitrates derivatives on the contraction induced by KCl in mesenteric artery rings.
